# Eukaryotic transcription factors can track and control their target genes using DNA antennas

**DOI:** 10.1038/s41467-019-14217-8

**Published:** 2020-01-28

**Authors:** Milagros Castellanos, Nivin Mothi, Victor Muñoz

**Affiliations:** 1 Instituto Madrileño de Estudios Avanzados en Nanociencia (IMDEA Nanociencia), Faraday 9, Campus de Cantoblanco, Madrid, 28049 Spain; 2 Centro Nacional de Biotecnología, Consejo Superior de Investigaciones Científicas (CSIC), Darwin 3, Campus de Cantoblanco, Madrid, 28049 Spain; 3 Department of Bioengineering, School of Engineering, University of California, 95343 Merced, CA USA

**Keywords:** Biophysical chemistry, DNA, Molecular conformation, Thermodynamics, Single-molecule biophysics

## Abstract

Eukaryotic transcription factors (TF) function by binding to short 6-10 bp DNA recognition sites located near their target genes, which are scattered through vast genomes. Such process surmounts enormous specificity, efficiency and celerity challenges using a molecular mechanism that remains poorly understood. Combining biophysical experiments, theory and bioinformatics, we dissect the interplay between the DNA-binding domain of Engrailed, a *Drosophila* TF, and the regulatory regions of its target genes. We find that Engrailed binding affinity is strongly amplified by the DNA regions flanking the recognition site, which contain long tracts of degenerate recognition-site repeats. Such DNA organization operates as an antenna that attracts TF molecules in a promiscuous exchange among myriads of intermediate affinity binding sites. The antenna ensures a local TF supply, enables gene tracking and fine control of the target site’s basal occupancy. This mechanism illuminates puzzling gene expression data and suggests novel engineering strategies to control gene expression.

## Abstract (web summary)

To carry out their function, transcription factors must efficiently recognize specific DNA sequence targets, a complex problem in the context of eukaryotic chromatin. Here the authors use single-molecule biophysical experiments, statistical mechanical theory and bioinformatics analyses to conclude that interactions with non-target sequences near promoters serve to increase overall affinity and targeting efficiency.

## Introduction

Transcription factors (TF) control gene expression by binding to their target DNA site to recruit, or block, the transcription machinery onto the promoter region of the gene of interest. Their function relies on the ability to find their target site quickly and selectively^1^. In living cells TFs are present in nM concentrations and bind the target site with comparable affinity^2^, but they also bind any DNA sequence (nonspecific binding)^3^, resulting in millions of low affinity (i.e., >10^−6^ M) competing sites. Nonspecific binding facilitates the search for the target site by allowing the TF to slide along DNA via a relatively slow, but more efficient, one dimensional diffusive motion (D < 10^−8^ cm^2^s^−1^)^4^ that involves rotation about the DNA axis^5^ and covers distances between 300 and 10,000 bp^6^. Another mechanism of facilitated diffusion occurs when the TF is transferred between DNA regions in transient spatial proximity^7^. These various nonspecific binding modes act jointly to speed up the TF recognition of its target site^8^. For instance, in vivo imaging experiments in bacteria indicate that the combination of these molecular elements suffice to explain the homing, selectivity and occupancy of prokaryotic TFs^9^.

Eukaryotic gene expression is much more complex and operates in multiple layers, including dynamic control over the chromatin structure^10,11^ and epigenetic factors^12^. But even at the molecular level, achieving efficient transcription control is much more challenging than in prokaryotes^13^. Eukaryotic genomes are orders of magnitude larger^2^ and their TFs feature much shorter DNA recognition sites (6–10 bp)^13,14^, leading to hundreds of random occurrences for any consensus sequence, and thus to inevitably impaired specificity and site occupancy^15^. Moreover, each eukaryotic TF controls tens to hundreds of genes scattered throughout the genome^16,17^, and expressing each gene needs various TFs simultaneously binding to their sites to form the transcription complex^18^, an extremely rare event in probabilistic terms. As result, the in vivo site occupancy patterns of eukaryotic TFs are more complex than predicted by their in vitro site-specific binding profiles^19,20^ and do not strongly correlate with the actual levels of gene expression^17,21,22^. Intriguingly, single-molecule fluorescence tracking in mammalian cells has shown that the TF Sox2 finds one of its target sites in fewer than 100 binding attempts^23^, suggesting that it only samples a miniscule fraction of the transcriptionally accessible genome (i.e., ~2% of 2.5 Gbp^24^).

An interesting feature highlighted by genome analysis is an accumulation of potential TF binding sites in regions flanking eukaryotic genes^15^. Such clusters of degenerate recognition sites are assumed to be key for transcription control^25^, and thus are generally classified as gene regulatory regions (RR)^26^. The potential roles that repetitive sequence patterns flanking the cognate site may play on how eukaryotic TFs find their target have been recently subject to intense scrutiny. For instance, when surrounding the target site, certain symmetric sequence repeats can affect the TF binding preference by simple statistical (or entropic) factors rather than by specific base recognition^27–29^. Existing DNAse footprint data reveals that clusters of spatially proximal enhancers (or archipelagos^30^) correlate with increased TF occupancy in vivo^31^. Moreover, theoretical modeling indicates that a flanking DNA sequence that is heterogeneous^32^, or contains weakly competing binding sites^33^, could accelerate the TF search for its target site. However, the molecular aspects of the interaction between TF and these flanking DNA regions have not yet been established, nor is there a mechanism available that integrates binding to these regions with canonical specific and nonspecific DNA binding to enable efficient eukaryotic transcription. Here we address this fundamental question investigating the interactions between the DNA-binding domain of a eukaryotic TF and the regulatory regions of genes under its transcriptional control. We utilize biophysical methods to dissect the binding process, statistical mechanical modeling to integrate and rationalize the results, and bioinformatics analysis to further explore the functional implications.

## Results

### A model of eukaryotic transcription factor binding to target gene

**Fig. 1:**
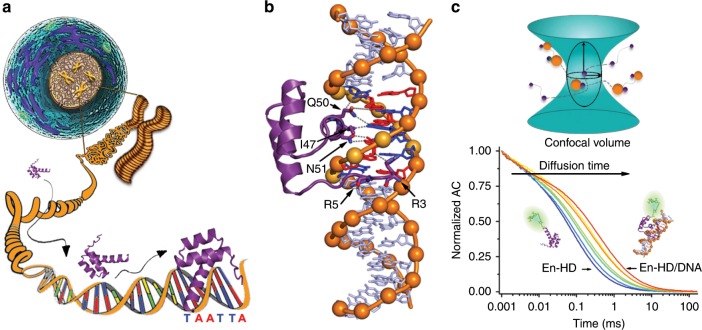
EngHD binding to the target specific site. **a** Pictorial representation of the challenges involved in tracking the target cognate site in the genomic DNA of a eukaryotic cell. **b** 3D Structure of the specific complex between EngHD and DNA (PDB: 1HDD). **c** Schematic diagram of how to determine binding of EngHD to DNA using FCS. (top) The miniscule illumination volume of a confocal microscope is used to monitor fluctuations in the emission of fluorescent-labeled EngHD molecules (purple) while in diffusive transit. When bound to the much larger DNA molecule (orange), EngHD experiences delayed diffusion, staying longer within the illumination volume. The fluorescence autocorrelation decay (bottom) represents the average diffusion, which depends on the fraction of free and bound EngHD molecules. FCS experiments at various concentrations of DNA permit to accurately determine the dissociation constant from the combined autocorrelation decays.

We focus on Engrailed, a TF from *Drosophila melanogaster* involved in defining embryonic parasegmental subdivision^34^ and maintaining parasegmental borders in adult appendages^35^. Engrailed controls the expression of over 200 different genes in *Drosophila*^36^. Its DNA-binding domain (EngHD) folds into a three-helix bundle that recognizes the palindromic sequence TAATTA as its consensus site^37^. EngHD binds to DNA inserting its C-terminal α-helix into the DNA major groove to engage in specific interactions with consensus bases (Fig. 1a, b). Binding is reinforced by electrostatic interactions with the phosphate backbone (Fig. 1b). As example of gene regulatory region (RR), we selected a fragment from the β3-tubulin gene, which is repressed by Engrailed^38^. This fragment (bases 2769–2918 from the transcription start site) falls within the first intron^39^, and contains one specific binding site that slightly diverges from the consensus (TAATTG), but retains relatively high affinity^38^. We employed fluorescence correlation spectroscopy (FCS) as biophysical method to characterize the interactions between EngHD and the β3-tubulin RR in quasi-single particle conditions. In FCS, a miniscule confocal volume (~1 fL) is illuminated so that the diffusive paths of individual fluorescent molecules are detected from correlated fluctuations in fluorescence emission (Fig. 1c). Free and bound molecules are identified based on their different diffusive properties. In our case, we use a fluorescent-labeled version of EngHD at ~1 nM in the presence of varying concentrations of unlabeled dsDNA, and determine the fraction of EngHD bound to DNA from the retarded diffusion of the complex (Fig. 1c) (see Methods). FCS is optimally suited for our purposes because it is sensitive to binding in sub-nM to μM range and measures DNA association directly, whether such association comes from one (specific) or many (nonspecific) binding sites.

### Specific versus nonspecific DNA-binding contributions

**Fig. 2:**
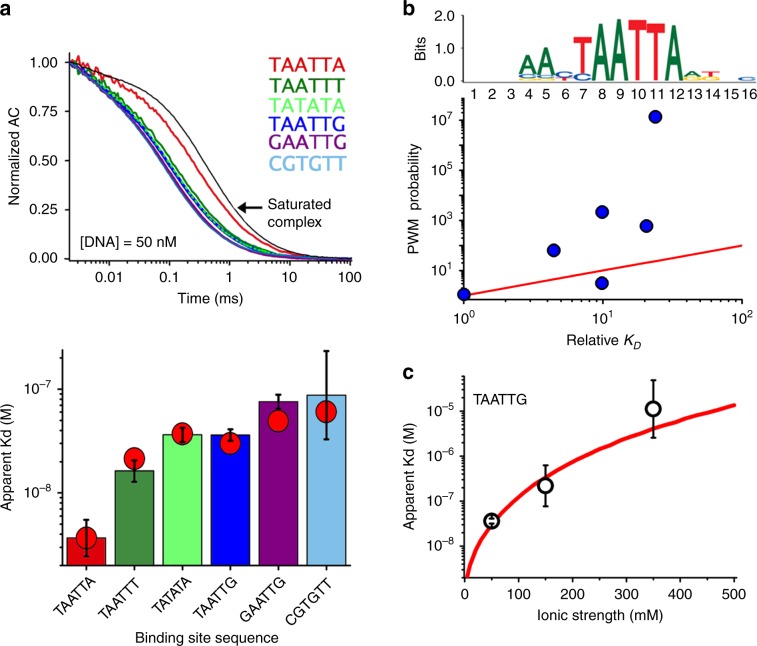
Mapping the energetics of EngHD binding to DNA. **a** (top) Experimental FCS autocorrelation decays of EngHD in the presence of 50 nM of each of the six 75-bp DNA molecules based on the β3 tubulin gene with variations in the SB site. The average diffusion time relative to diffusion of the saturated complex (black curve) reflects the fraction of bound molecules. (bottom) Dissociation constants for the six 75-bp DNAs determined experimentally by FCS from three independent experiments and calculated by the statistical mechanical model (red circles). **b** Experimental changes in binding affinity compared with the changes expected by pure consensus binding. The upper panel shows the consensus binding logo for engrailed obtained from bacterial one-hybrid high throughput assays. The lower panel shows the correlation between the experimental changes in binding affinity, *K*_*D*_(variant) · *K*_*D*_(specific)^−1^, in the abscissa; and the inversed relative probability of binding calculated from the position weight matrix, *p*_*PWM*_ (specific) · *p*_*PWM*_(variant)^−1^, in the ordinate. **c** Ionic strength dependence of EngHD binding to the 75-bp DNA molecule bearing the natural TAATTG high affinity site (dark blue in **a**). Experimental data are shown as black open circles and the statistical mechanical model calculation is shown as a red curve. Bars delimit the 95% confidence interval, see Supplementary Table 2. Source data are provided as a Source Data file.

We analyzed the contributions to EngHD’s binding affinity using a series of DNA molecules based on the 75-bp central segment of the β3-tubulin intron in which we modified the specific binding site (Supplementary Table 1). FCS experiments on the consensus variant (TAATTA) rendered a dissociation constant (*K*_*D*_) of ~4 · 10^−9^ M (Fig. 2a, Supplementary Table 2) that is consistent with previous measurements on a non-natural DNA sequence using gel-shift assays^38,40^. Experiments on the remaining DNAs showed affinity decreases proportional to the divergence from the consensus sequence (Fig. 2a, Supplementary Table 2). TAATTT exhibited a 4-fold decrease in affinity. Permutation of the two central bases (TATATA) and replacement of the last base by G rendered 10-fold decreases. The simultaneous change of the two end bases to G, or a highly divergent binding site (CGTGTT) resulted in 19- and 22-fold affinity drops, respectively. These experiments reveal evident changes in affinity. However, the affinity decrease is small relative to how much the binding site diverges from the consensus, most notably for CGTGTT in which only one consensus base is retained. To investigate this issue, we compared these results with the position weight matrix (PWM) for Engrailed obtained from bacterial one-hybrid assays^41^. The PWM recapitulates the consensus binding site (Fig. 2b) and predicts a decreasing binding probability as the target site diverges from consensus. However, the PWM predicts changes many orders of magnitude larger than what we find experimentally (Fig. 2b). The PWM also predicts huge differences in binding to target sites that exhibit very similar affinity in the context of the 75 bp β3-tubulin intron DNA. The implication is that EngHD DNA binding is more complex than dictated by specific interactions with the consensus motif. The most likely explanation is that EngHD binds promiscuously to the flanking DNA sequence, thereby buffering the degradation or even elimination of the consensus site.

An obvious factor driving promiscuity are electrostatic interactions, which contribute to the stabilization of the specific binding site^42^, but also promote nonspecific binding to any other site along a given DNA molecule^3^. The 3D structure of the EngHD-DNA complex^40,43^ highlights attractive electrostatic interactions formed between positively charged side-chains in EngHD and the DNA phosphate backbone (Fig. 1b), consistently with reports on other DNA-binding domains^42,44,45^. To establish the role of these interactions, we investigated how ionic strength affects EngHD’s binding to these DNA molecules. The ionic strength does indeed strongly decrease DNA affinity (i.e. by 200-fold between 50 mM and 350 mM NaCl; Fig. 2c). Therefore, at moderate ionic strengths, electrostatic interactions are a key contributor to the affinity of EngHD for DNA. However, is noteworthy that the affinity changes induced by alteration of the target site exhibit a sequence dependent pattern different from the PWM. For instance, we see that A/T swaps induce smaller affinity drops than changes to G or C, which suggests that there is more to the promiscuous EngHD binding than canonical nonspecific binding via electrostatic interactions.

### A simple theoretical model of EngHD-DNA-binding energetics

**Fig. 3:**
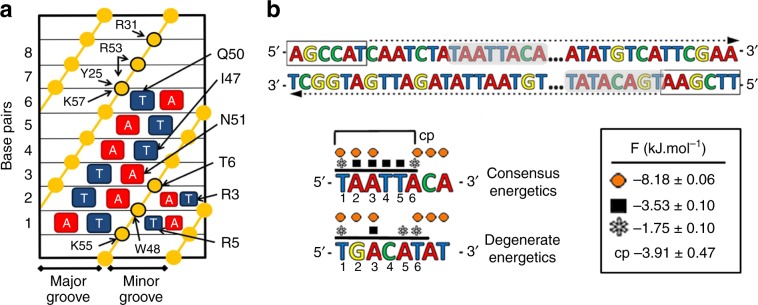
Statistical mechanical model to describe the DNA-binding modes of EngHD. **a** Scheme of the various interactions existing between EngHD and DNA as observed in the 3D structure of the EngHD-DNA complex (based on ref. ^40^, PDB: 1HDD). **b** (top) Representation of how the statistical mechanical model calculates all possible binding events using a 6-bp sliding window that runs 5′–3′ through both strands. (bottom) detailed binding energetics for the two exemplary binding sites selected from the top (shown on top as gray boxes). The upper example includes all the specific binding interactions of a full consensus site, and the lower is an example of a degenerate consensus site. The model and interactions are described in the text, and the parameters obtained after global optimization against all experimental data (Figs. 2, 4 and 5) together with their statistical significance (one standard deviation) are given in the box. Black symbols correspond to consensus core tetrad interactions (${\mathrm{\Delta }}G_{{\mathrm{consensus}},{\mathrm{core}}}$), gray asterisks correspond to degenerate consensus interactions with A or T (${\mathrm{\Delta }}G_{{\mathrm{degenerate}},{\mathrm{AT}}}$). Orange circles correspond to electrostatic (nonspecific) interactions, which extend over 8-bp (${\mathrm{\Delta }}G_{{\mathrm{elec}}}$) and cp is the cooperative interaction that takes place when the site includes the full consensus sequence ($2{\mathrm{\Delta }}G_{cp}$; see model description).

To quantitatively rationalize the complex DNA-binding properties of EngHD we built a statistical mechanical model that considers binding to any 6-bp site available in any given DNA molecule (Fig. 3). Particularly, we implemented two versions of the model energetics: one empirical version based on the Engrailed PWM matrix, and another version inspired by the 3D structure of the EngHD-DNA complex^40^ (Fig. 3a). Both models, the fitting to the experimental data and the resulting parameters are described in Methods. We find that the PWM model reproduces all of the data at a semiquantitative level using only one fitting parameter, whereas the structure-based model fits the data over 50-fold better using four parameters (see Supplementary Fig. 1). A Fisher test indicates that the probability that the statistically simpler model (PWM) is equivalent to the parametrically complex (structure-based) model is below 10^−9^. We thus focused on the structure-based model (Fig. 3b) for all subsequent analyses. The fits to, and predictions from, the structure-based model are shown as red circles and/or red curves throughout the article (e.g. Fig. 2a, c).

The ability of the structure-based model to reproduce the non-trivial changes in affinity that we observe suggests that it captures the fundamental energetics of EngHD binding to DNA. Such binding energetics confirm the existence of a third, non-canonical, DNA-binding mode in which EngHD binds promiscuously to degenerate consensus repeats. From here onwards we denote these three DNA-binding modes as: (1) specific binding to a consensus site (SB); (2) degenerate consensus binding (DCB), which refers to binding to any other site with a partial consensus sequence; (3) nonspecific binding (NSB), defined as sequence-independent, electrostatically driven binding^3^. A similar, semi-specific binding of eukaryotic TFs to clusters of degenerate consensus repeats around a cognate site has been proposed to increase the site’s occupancy in vivo^30,31^ and to accelerate the search for the target site^33^. Here we determine its actual contribution to binding and dissect its molecular mechanism. The key questions that emerge are: how does the interplay of these three binding modes define the overall binding behavior of EngHD? And what are its functional implications?

### The binding free energy landscape flanking the target site

**Fig. 4:**
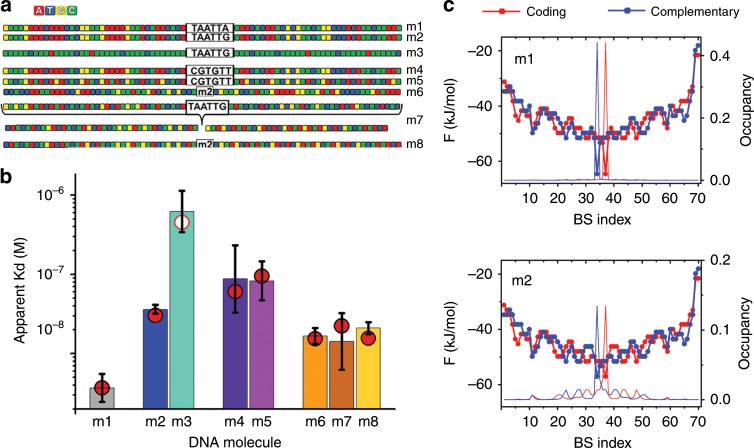
The region around the specific site produces a rugged free energy landscape. **a** DNA molecules used to explore the effects of the sequence of the region flanking the specific site. **b** Dissociation constants for the DNA molecules shown in panel **a** obtained by FCS from three independent experiments, and calculated by the statistical mechanical model (red circles). **c** Examples of binding free energy landscapes and EngHD occupancy profiles obtained by the model. BS index indicates the position of the first base of each potential binding site in the coding or complementary strands. The free energy scale is given on the left, and the probability of each site being occupied on the right *y*-axis. The occupancy profiles have been calculated at the same DNA concentration of 10 nM: just above the *K*_*D*_ for m1 and below the *K*_*D*_ for m2. Bars delimit the 95% confidence interval, see Supplementary Table 2. Source data are provided as a Source Data file.

Our theoretical analysis points to DCB as modulator of EngHD’s affinity for the β3-tubulin first intron. The DNA sequence flanking the target site is indeed rich in A/T clusters^39^ that feature many potential DCB sites (see m2 in Fig. 4a). The calculated binding profile of the whole DNA molecule shows a rugged binding free energy landscape with many minima. The local minima concentrate around the target site, producing an overall funnel for EngHD binding (m1 in Fig. 4c), a property reminiscent of the energy landscapes associated to protein folding^46^, binding and function^47^. Interestingly, binding to many mid-affinity sites around the target site could be a strategy to enhance overall affinity without involving strong specific interactions, that is, maintaining relatively fast dissociation rates. Such behavior is consistent with theoretical predictions^33^. A rugged funneled binding landscape also introduces resilience to mutations on the target site, exactly as we observe experimentally (Fig. 2a). This effect becomes evident by comparing the occupancy profile of the natural DNA (with TAATTG) and the DNA carrying the consensus (SB) site. The landscape of the latter features a global free energy minimum that concentrates most occupancy (m1 in Fig. 4c), whereas the natural sequence lacks the sharp minimum but maintains all flanking DCB sites (m2 in Fig. 4c), which in absence of a competing SB site see their occupancy raised, thereby buffering the overall drop in affinity.

To further investigate this phenomenon, we designed a 75 bp DNA that carries the original β3-tubulin high affinity site (TAATTG), but minimizes A–T content everywhere else, and thus eliminates DCB sites (m3 in Fig. 4a). m3 shows a 21-fold decrease in binding affinity relative to m2 (*K*_*D*_ of ~0.7 · 10^−6^ M)(Fig. 4b). Such drop is striking because the two DNAs have the same target site. We hence confirm that DCB dominates the overall binding to the β3-tubulin intron region. The statistical mechanical model underestimates the affinity drop, presumably because this simple model does not account for the formation of secondary structure that takes place in this G/C-rich DNA (Supplementary Fig. 2) and which is likely to further impair EngHD binding. In fact, our experimental result is close to the model prediction for the same flanking sequence bearing the low affinity CGTGTT on the target site (open circle in Fig. 4b). An alternative explanation for this result could be the potential accumulation of symmetric base repeats in the flanking region, a factor proposed^27^, and found in the TF MAX^28^, to entropically stimulate binding in the absence of base-specific interactions. The β3-tubulin intron fragment does indeed contain many base repeats (m2 in Fig. 4a). We thus tested this possibility using a DNA that maintains the β3-tubulin original base composition, but it eliminates base repeats (m5 in Fig. 4a). We also used the low affinity CGTGTT as target site, aiming to minimize SB contributions and thus increase the experimental sensitivity to differences between DCB and NSB. The statistical mechanical model does not include nonspecific effects from symmetric base repeats, and, accordingly, it calculates minimal binding differences between m5 and the β3-tubulin sequence (m4). FCS experiments also show minimal differences (Fig. 4b), confirming that the flanking DNA effects we see in EngHD arise from promiscuous DCB instead of from nonspecific base repeats. Interestingly, the flanking effects appear to extend over relatively long distances, as suggested by the two-fold higher affinity of the 150-bp β3-tubulin fragment (m6) relative to the 75-bp version (m2; Fig. 4b). Experiments on other 150-bp DNA molecules further confirm that, in absence of a SB site, the affinity increases proportionally to the availability of DCB sites. For instance, shuffling the entire 150-bp sequence (m7) or the external region (m8), does not change the affinity in either experiments or model calculations (red circles for m6, m7 and m8 in Fig. 4b).

### Contribution from promiscuous binding to degenerate consensus sites

**Fig. 5:**
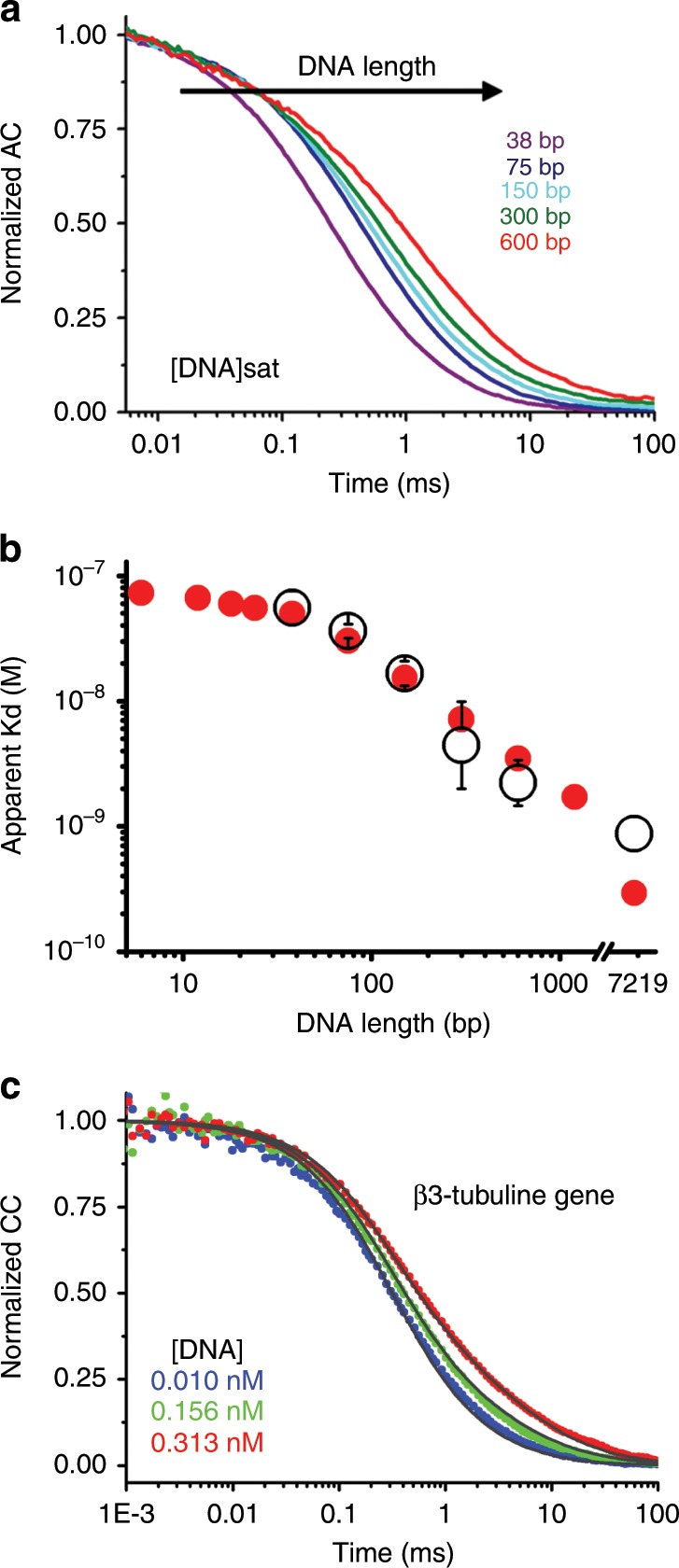
Amplification of EngHD binding affinity induced by the flanking region. **a** FCS autocorrelation decays of EngHD measured in the presence of saturating concentrations of DNA molecules of different size. **b** Dissociation constants of EngHD binding to DNA molecules of varying size determined experimentally by FCS from three independent experiments on the 5 DNAs of panel **a** (open black circles), and predicted by the model (red circles). The data at 7219 bp corresponds to the affinity of the β3-tubulin gene (panel **c**) with experiment and model prediction as before. **c** FCS cross-correlation decays of EngHD measured in the presence of various concentrations of a DNA molecule encompassing the entire β3-tubulin gene sequence (see Fig. 6b). Bars delimit the 95% confidence interval. Source data are provided as a Source Data file.

A key question is whether promiscuous DCB is just localized near the SB or propagates over the full RR of EngHD target genes. This consideration is important given that eukaryotic cis-acting RR extend over thousands of bp, and are often located far (>50 Kb) from the transcription starting site^48,49^. The long eukaryotic RRs could potentially exploit DCB to massively amplify the binding affinity of relevant transcription factors. To investigate this hypothesis, we designed a series of DNA molecules based on the β3-tubulin intron but with varying size (38, 75, 150, 300, and 600 bp). In FCS experiments these DNA molecules diffuse with coefficients that decrease proportionally to their size (Fig. 5a) as expected from the known length dependence of DNA’s translational diffusion coefficient^50^. Binding experiments showed a remarkably strong amplification of EngHD binding as its natural DNA partner grows in length: from *K*_*D*_ ~ 5.6 · 10^−8^ M for the 38-bp DNA to ~2.2 · 10^−9^ M for the 600-bp molecule, or a ~25-fold increase for a 15-fold longer DNA that does not incorporate extra SB sites (Fig. 5b). Exploring by FCS the flanking sequence effects over shorter or longer DNA scales is difficult due to technical limitations: 38-bp is the shortest DNA that results in a complex with diffusion coefficient clearly distinguishable from that of free EngHD, and the affinity of the 600-bp is close to the detection limit. However, the statistical mechanical model, which recapitulates these experimental trends (red circles in Fig. 5b), predicts a 300-fold affinity increase for the entire β3-tubulin gene^51^, relative to its unique high affinity SB site (Fig. 5b). This strong amplification implies that EngHD binding to the β3-tubulin gene is in fact dominated by promiscuous binding to DCB sites, which win over SB by virtue of their vast numbers, even though each site has relatively low affinity (i.e., ~10^−7^ M).

To determine whether binding amplification scales up to full genes, we synthesized a 7.2 kbp DNA encompassing the β3-tubulin gene sequence (including introns, but without 5′ and 3′ UTR). FCS experiments of EngHD in the presence of pM concentrations of this DNA molecule confirm very strong binding, which is noticeable even at ~150 pM (Fig. 5c), indicating a *K*_*D*_ in the sub-nM range (Fig. 5b). These experiments demonstrate that the binding amplification induced by DCB extends over the DNA scales of full genes. As result, the affinity for the entire β3-tubulin RR is orders of magnitude higher than binding to just its original, high affinity target site. In other words, DCB transforms the β3-tubulin RR onto a potent attractor for EngHD molecules. It follows that such binding pattern ensures local availability of the transcription factor as well as low occupancy of the specific site, and thus may operate as a transcription antenna.

### The binding profile of a transcription antenna

**Fig. 6:**
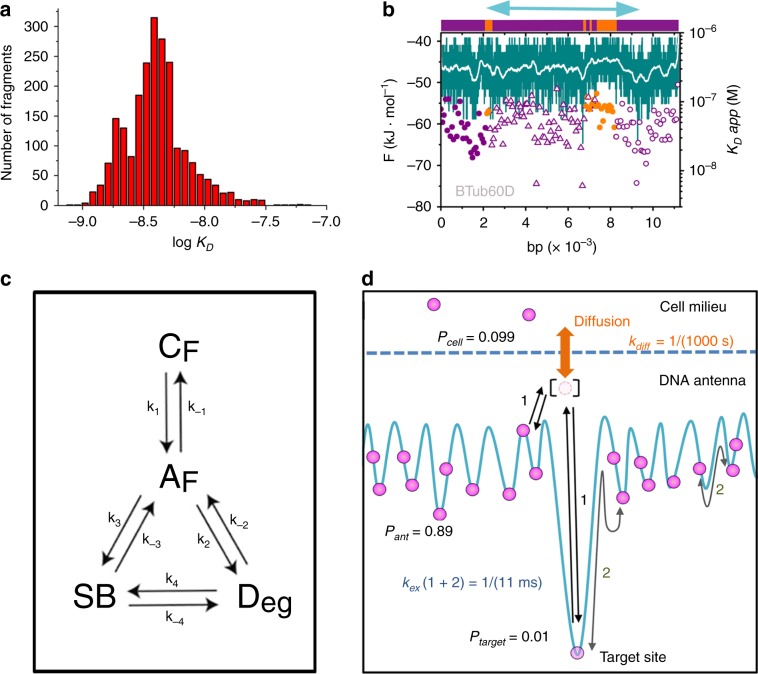
Transcription antennas for tracking and controlling the gene of interest. **a** Histogram of binding affinity (in log10(Molar) units) predicted by the statistical mechanical model for all of the DNA fragments (between 100 and 476 bp long) from the Drosophila genome identified to bind to EngHD in ChIP-Seq experiments on transgenic flies expressing en-GFP fusion proteins (ENCODE project experiment ENCSR952TDU). **b** Profile for EngHD binding to the extended (including 2 kbp before and after) β3 tubulin gene (FlyBase code βtub60d, corresponding to the chromosome region: 2 R 24,305,881–24,313,099) calculated with the statistical mechanical model. The gene organization is shown as a bar on top (exons in orange; 5′ UTR, 3′ UTR and introns in purple). Dissociation constants calculated for successive regions of 75 bp follow the same color scheme with closed circles for 5′ UTR and exons, open circles for 3′ UTR and open triangles for introns. The light blue arrow on top signals the specific region synthesized for the binding experiments of Fig. 5c. The binding free energy for the coding strand is shown in teal. **c** The four-state model describing the kinetic operation of a transcription antenna. The description of the model (states and rate constants) and its physical properties are given in Methods. **d** Diagram representing the β3 tubulin transcription antenna four-state kinetic model implemented with parameters inspired by our experimental results (see Methods). Hundreds of degenerate binding sites (*p*_*ant*_ is the population of *D*_*eg*_) are in fast exchange with each other and with the specific target site (*p*_target_ is the population of *SB*) by dissociation and rebinding events within the antenna (1) and by 1D diffusion (2). The exchange with the cell milieu (*p*_cell_ is the population of *C*_*F*_), which is shown as an orange arrow, is governed by diffusion between the miniscule population of free EngHD molecules within the antenna (square brackets, *A*_*F*_) and EngHD molecules outside of the antenna.

The fingerprint of transcription antennas would be the accumulation of DCB on the regulatory (noncoding) regions of the gene as opposed to the coding regions (exons). We can investigate this question bioinformatically by calculating the EngHD binding profiles for other gene sequences. Before embarking on large-scale DNA sequence profiling, however, we tested the biological significance of the predictions of our model by calculating the binding affinity of the 2226 DNA fragments (each between 100 and 500 bp long) that have been identified in ChIP-Seq experiments as DNA regions that bind Engrailed in vivo. The model predicts high binding affinity for all ChIP-Seq fragments, with over 90% of the fragments’ predicted *K*_*D*_ values between 1 and 10 nM (Fig. 6a). These affinities are comparable to what we have measured in vitro for the 150 bp segment from the first intron of β3-tubulin carrying the consensus site (Fig. 4b). We can thus conclude that the binding predictions of the statistical mechanical model are biologically significant. In this regard, we note that the binding profile of the full β3-tubulin gene (containing the 5′ and 3′ UTR regions) has a distinct pattern of dense local clusters of DCB sites found in the noncoding regions (magenta in Fig. 6b) together with an absence of them in exons (orange in Fig. 6b), which is what we expect for a transcription antenna. Hence, the β3-tubulin gene sequence maximizes EngHD binding along the RR so that molecules of EngHD become localized around the target gene via promiscuous DCB binding, whereas the exons remain unoccupied. In *Drosophila* cells, EngHD is present at 1–10 nM concentrations^2^, which suggests that the β3-tubulin RR will host several EngHD molecules at all times. Here it is important to note that the overall affinity is high, but each DCB binding event has moderate affinity (*K*_*D*_ ~ 10^−7^ M), and therefore a relatively high dissociation rate that could permit the fast interconversion between sites.

### A kinetic model of transcription antennas

The binding profile of the β3-tubulin RR immediately suggests a role as efficient tracking device that limits the TF search for the SB to within the antenna limits, rather than over the entire genome (or cell). Such a transcription antenna could be also used to control the SB occupancy at the levels required for biological function. To quantitatively explore these ideas, we built a very simple kinetic model of a transcription antenna. The model is composed of four species connected kinetically as shown in Fig. 6c, and it assumes that the region of interest is transcriptionally active. For any such accessible gene, the relevant TF is in either of the four states: bound to DCB sites in the antenna (*D*_*eg*_), bound to the specific site (*SB*), in the small cellular volume surrounding the antenna (*A*_*F*_), or in the cellular milieu (*C*_*F*_). Unbound TF molecules enter and escape the antenna space by diffusion (modeled with rate constants k_1_ and k_−1_). Once inside the antenna, free TF molecules (*A*_*F*_) bind to any DCB site (all make *D*_*eg*_) via k_2_, and to *SB* via k_3_. The TF can be released back to *A*_*F*_ from the bound states via k_−2_ and k_−3_, respectively. Finally, the TF can reach *SB* from sites within *D*_*eg*_ or vice versa via one dimensional diffusion (sliding and/or intersegment transfer), represented by k_−4_ and k_4_. The details of the model, rate matrix, and the interpretation of its eigenvalues are provided in Methods. To explore its functioning, we implemented it with parameters (also given in Methods) inspired by our results on the β3 tubulin gene and general properties of Engrailed and *Drosophila* cells, aiming simply to represent a plausible scenario. These calculations confirm the anticipated mode of operation, which is shown graphically in Fig. 6d. As illustrated in the figure, the antenna accumulates the largest population of TF molecules in very slow exchange with the cell milieu (TF either free or bound somewhere else in DNA) because the diffusive exchange depends on the infinitesimally small probability of finding an unbound TF within the antenna (*A*_*F*_, square bracketed species in Fig. 6d). Therefore, the antenna effectively traps TF molecules in myriads of binding events, thereby locally buffering any changes in accessibility of distant DNA due to chromatin dynamics^52,53^, or in TF concentration at the cellular level. In contrast, TF molecules within the antenna (including the target site, *SB*) remain in fast, millisecond exchange (*k*_*ex*_ in Fig. 6d), either by dissociation—facilitated by the relatively high dissociation rates of DCB sites—and quick rebinding (1 in Fig. 6d), or through sliding (2 in Fig. 6d). These general effects are also consistent with recent theoretical work^33^, and demonstrate how a transcription antenna can guarantee a local supply of TF, quick turnover over the SB site, and fine control of *SB* occupancy via binding competition with DCB sites.

### Genes regulated by Engrailed contain transcription antennas

**Fig. 7:**
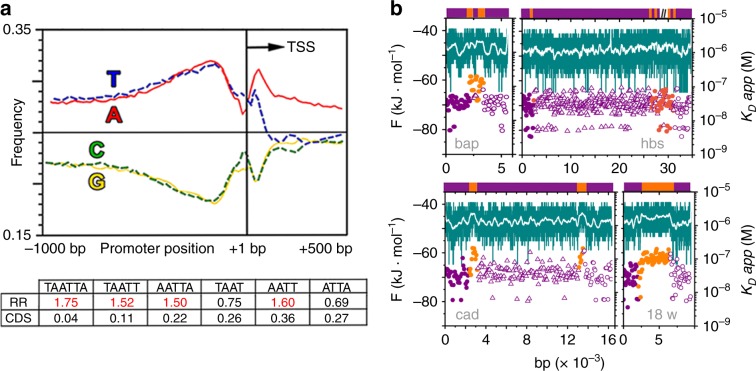
The genes controlled by Engrailed contain transcription antennas. **a** Nucleotide composition observed in the promoter regions of *Drosophila* (figure based on ref. ^55^). TSS indicates the transcription start site observed vs. expected frequency of full, quintet and quartet consensus sites in all of the *D. melanogaster* genes known to be regulated by Engrailed. RR, regulatory regions; CDS, coding sequence. **b** Examples of EngHD binding profiles for *D. melanogaster* genes under control by Engrailed (*bap, hbs, cad*, and *18w*) predicted by the statistical mechanical model. The colors and symbols are as in Fig. 6b. The results for the 39 genes used in the analysis are summarized in Supplementary Table 3.

A follow-up question is whether transcription antennas are specific to the β3 tubulin gene or broadly used by Engrailed. At least 203 *Drosophila* genes are controlled by Engrailed^36^. The *Drosophila* genome is in general A/T rich (about 57%)^54^, but it has a spike in A/T content in the gene promoter regions and a downward trend after the transcription site (Fig. 7a)^55^. This pattern is consistent with transcription antennas. For further bioinformatic analysis, we focused on the 39 genes that are best characterized as being under Engrailed control (Supplementary Table 3). The profile of transcription antennas in these genes is apparent even via simple sequence analysis. For instance, the consensus sextuplet (TAATTA), the two possible quintets and especially the central core quartet (AATT), are all overrepresented in RRs (5′ UTR, 3′ UTR and introns) and heavily underrepresented in coding regions (CDS) (Fig. 7a and Supplementary Table 4) relative to the random expectation for 57% AT content. However, the organization of gene RRs as transcription antennas is most apparent in the EngHD binding landscapes of complete gene sequences. Figure 7b shows four examples, which reveal the same pattern of the β3-tubulin gene (Fig. 6b). The results for all 39 genes are given in Supplementary Table 3. In all cases, the exons (orange) feature relatively weak binding affinity, whether the gene contains multiple short exons (e.g., hbs) or a single long one (e.g., 18w). Binding to noncoding regulatory regions is, on the other hand, uniformly of high affinity. As negative control, we looked into the binding patterns for genes unlikely to be under Engrailed control (see Methods). Although it is not possible to entirely rule out Engrailed control, the binding landscapes for the genes we selected as negative controls show significantly lower affinity for EngHD than the 39 genes of Supplementary Table 3, and no patterns of higher/lower affinity for noncoding versus coding regions (Supplementary Fig. 3 shows six examples). We thus conclude that genes under Engrailed control contain transcription antennas for this TF, whereas this DNA organization is not present in genes without such control. Altogether, our results provide compelling evidence of a systematic usage of transcription antennas by Engrailed.

## Discussion

Eukaryotic TFs track their target genes, control site occupancy, and coordinate binding with partners to form the transcription complex. These processes must involve modes of interaction with DNA that go beyond nonspecific binding and facilitated 1D and/or 2D diffusion^56,57^. By focusing on the DNA regions flanking the target site of real genes, we have discovered, and characterized, a molecular mechanism that enables such functions. The mechanism exploits the natural tendency of biomolecules to exhibit energetic frustration^46,47^, in this case manifested by binding promiscuity. Particularly, we find that the affinity of the *Drosophila* TF Engrailed to the RRs of its target genes is strongly amplified by long tracts of degenerate consensus repeats that are present in such regions. The combination of a promiscuous TF and a DNA region rich in DCB repeats operates as a transcription antenna. Once the DNA region becomes accessible by chromatin dynamics^52^, and thus transcriptionally active, the antenna attracts TF molecules that remain loosely associated to the gene of interest through a highly dynamic exchange among the hundreds of mid-affinity binding sites (<SB but ≫ NSB) within the antenna. In this light, we confirm that the short recognition sequences and promiscuous specific binding of eukaryotic TFs are a functional strategy to ensure their colocalization with the relevant genes, as it has been postulated by other authors^29,31^. For instance, there are ~30,000 copies of Engrailed per cell^17^ and about 200 genes estimated to be under its control^36^. Taking the β3 tubulin antenna as example (Fig. 6b), it follows that each of these 200 genes will contain on average ~150 EngHD molecules trapped in its antenna, whereas fewer than 15 molecules will be found anywhere else in the cell (bound non-specifically or free). The pool of TF molecules inside an antenna will be in exchange between sites that are relatively weak binders, so their faster dissociation rates facilitate turnover over the SB site, and thus enable a nimble gene expression response (Fig. 6d).

The antenna mechanism sheds new light onto some puzzling observations of eukaryotic gene expression. The mechanism predicts two “specific” binding modes: a frequent, still physically localized, but weaker binding event to antenna DCB sites, and a rare, high affinity binding to target sites (SB). These properties are in striking accord with single-molecule TF tracking experiments in mammalian cells^23^, which have reported that only ~1% of detectable binding events (with lifetimes >0.5 s) were to high affinity sites, whereas the remainder involved moderately weak binding events that seem too static to represent TF molecules sliding at 10^5^ bp^2^s^−1^ over DNA^5^. Antennas also enable control of the target site’s occupancy by competing locally for binding. A relatively distant (e.g., few kbp away) antenna can keep the basal SB occupancy of an activator at a suitable minimum and TF supply still relatively local. In contrast, an antenna surrounding the target recognition site can amplify a repressor’s effect. Binding events concentrated on long antennas provides a simple explanation of why crosslinking data on eukaryotes produces many more hits than expected from the number of genes under control of the given TF, and relatively weak correlations between site occupancy and gene expression levels^21,22,58^. Furthermore, the use of transcription antennas could greatly facilitate the synchronous recruitment of various TFs to assemble into the transcription machinery. Summarizing, DNA antennas provide an elegant mechanism that sheds new light on how eukaryotic TFs operate at the molecular level and explains several paradoxes of existing eukaryotic gene expression data. These molecular devices provide an additional layer of eukaryotic transcriptional control in which the size and sequence profile of the antenna can be engineered, whether by evolution or by scientists, to modulate site occupancy, response swiftness and levels of gene expression.

## Methods

### Statistical mechanical model for EngHD binding to DNA

The binding of EngHD to a DNA molecule of *N* base pairs contains a total of 2 · (*N*−5) potential binding sites (binding to all possible 6 bp sites in either strand). Defining the unbound state as reference (statistical weight, *w*_free_ = 1), the partition function for EngHD binding to DNA is thus:

1 $$Q\left( {\left[ {{\mathrm{DNA}}} \right]} \right) = \mathop {\sum }\limits_{i = 1}^{N - 5} w_i + \mathop {\sum }\limits_{j = 1}^{N - 5} w_j + 1$$

where *i* and *j* are dummy indexes that indicate the position in the DNA sequence of the first base of each 6-bp binding site on the coding and complementary strands, respectively, both running from the 5′–3′ end. The statistical weight for EngHD bound to DNA site *x* (whether on the coding or the complementary strand) is defined by the following equation:

2 $$w_x\left( {\left[ {{\mathrm{DNA}}} \right]} \right) = w_0\left[ {{\mathrm{DNA}}} \right]{\mathrm{exp}}\left( { - \Delta G_x^{{\mathrm{Binding}}}/RT} \right)$$

where *w*_0_ is a proportionality constant that represents the ratio between the diffusion controlled rate constant for association ($k_{{\mathrm{on}}}^0$; in M^−1^s^−1^) and the dissociation rate constant in the absence of interactions between EngHD and DNA ($k_{{\mathrm{off}}}^0$; in s^−1^). $\Delta G_x^{{\mathrm{Binding}}}$ is the binding free energy between EngHD and site *x*. Here we set *w*_0_ to 5 · 10^−4^ M^−1^, consistently with EngHD’s diffusion coefficient (determined by FCS as ~122 μm^2^s^−1^, see results). However, its exact value is of no practical consequence since it just scales $\Delta G_x^{{\mathrm{Binding}}}$. To define $\Delta G_x^{{\mathrm{Binding}}}$ as a function of the site’s sequence we developed two complementary interaction models inspired by the PWM and the 3D structure of the EngHD-DNA complex^40^. Both models follow the general formula:

3 $$\Delta G_x^{{\mathrm{Binding}}} = \left( {5 + \delta } \right)\Delta G_{{\mathrm{elec}}} + \mathop {\sum }\limits_{k = x}^{x + 5} \Delta G_k + \left[ {\delta _{16} + \left( {\delta _{15} + \delta _{26}} \right)/2} \right]\Delta G_{cp}$$

where δ is a Kronecker delta that takes a value of 1 for any binding site in a central position of the DNA (*x* ≥ 10 and *x* ≤ *N*−10) or 0 for any binding site located less than 1 turn from either DNA end (*x* ≤ 10 or x ≥ *N*−10) to account for end effects on the electrostatic interactions. $\Delta G_{{\mathrm{elec}}}$ is the free energy associated with each of the six possible electrostatic interactions between EngHD and the phosphate backbone, and depends on the ionic strength as $\Delta G_{{\mathrm{elec}}}\left( I \right) = \Delta G^0{\mathrm{exp}}\left( { - \sqrt I } \right)$ (Debye-Hückel treatment). The electrostatic term is identical for both models, and it is parameterized using the ionic strength dependence data (e.g., Fig. 2c). $\Delta G_k$ is the free energy of the EngHD specific interactions with each base on site x. In the PWM-based model, $\Delta G_k$ is directly defined according to the 6 × 4 position weight matrix (Fig. 2b) as $\Delta G_k = - {\mathrm{RTln}}\left( {p_k^B/0.25} \right)$, where $p_k^B$ is the probability of base *B* to be found in position *k* of the site, and $\Delta G_{cp} = 0.$ This is a model with 24 predetermined and one free fitting parameter ($\Delta G_{{\mathrm{elec}}}$). In the structure-based model, $\Delta G_k$is defined by two types of specific interactions: one interaction for each consensus base in the core tetrad (A_2_, A_3_, T_4_, T_5_) and one interaction for any other T or A present in the site (A_1_/T_1_, T_2_, T_3_, A_4_, A_5_, and A_6_/T_6_) (asterisk in Fig. 3b). The degenerate A/T interaction represents a structural preference of EngHD for the narrower minor groove and increased flexibility (shorter persistence length) of AT rich regions. δ_16,_ δ_15_, and δ_26_ are Kronecker delta that take a value of 1 when the binding site contains the full consensus, the first five or the last five consensus bases, respectively, and 0 otherwise. $\Delta G_{cp}$ is the cooperative binding free energy associated to formation of the specific consensus binding site. $\Delta G_{cp}$ accounts for: (a) the entropy loss of forming the specific complex, which is nearly complete when the protein locks onto the consensus core tetrad, and thus is not additive; (b) the extra hydrogen bonds that T_1_ and A_6_ can make with EngHD’s residues Q50 and N51, respectively (Fig. 3a), once the protein is forming the specific complex. As guidance, the two examples of binding sites shown in Fig. 3b lead to the following calculations for the binding free energy in the structured-based model: $\Delta G_{{\mathrm{TAATTA}}}^{{\mathrm{Binding}}} = 8\Delta G_{{\mathrm{elec}}} + 4\Delta G_{{\mathrm{cons,core}}} + 2\Delta G_{{\mathrm{deg}},{\mathrm{AT}}} + 2\Delta G_{cp}$; and $\Delta G_{{\mathrm{TGACAT}}}^{{\mathrm{Binding}}} = 8\Delta G_{{\mathrm{elec}}} + 1\Delta G_{{\mathrm{cons,core}}} + 3\Delta G_{{\mathrm{deg}},{\mathrm{AT}}}$. In both models the probability of EngHD binding to site x is simply,

4 $$p_x\left( {\left[ {{\mathrm{DNA}}} \right]} \right) = \frac{{w_x}}{Q}$$

and the overall probability of finding EngHD bound to DNA and unbound (free) are, respectively,

5 $$p_{{\mathrm{bound}}}\left( {\left[ {{\mathrm{DNA}}} \right]} \right) = \frac{{Q - 1}}{Q};$$

6 $$p_{{\mathrm{free}}}\left( {\left[ {{\mathrm{DNA}}} \right]} \right) = \frac{1}{Q}$$

The global dissociation constant for EngHD is easily obtained as the concentration of DNA at which $p_{{\mathrm{bound}}}\left( {\left[ {{\mathrm{DNA}}} \right]} \right) = p_{{\mathrm{free}}}\left( {\left[ {{\mathrm{DNA}}} \right]} \right) = 0.5$ (*Q* = 2). This global dissociation constant can be directly compared to the *K*_*D*_ values obtained from the FCS experiments.

We used the two statistical models to analyze our FCS experimental binding data. We first calibrated the overall interactions using the data from Fig. 2a (sequence dependence of specific interactions) and Fig. 2c (ionic strength dependence). We then globally fitted both models to all of the experimental data provided in this work to maximize convergence and determine the parameter’s statistical uncertainty. Fitting the PWM model globally led to $\Delta G_{{\mathrm{elec}}} = - 10.1 \pm 0.12 \,{\mathrm{kJ}}\;{\mathrm{mol}}^{ - 1}$. Fitting the structure-based model globally led to the following parameters: ${\mathrm{\Delta }}G_{{\mathrm{consensus,core}}} = - 3.53 \pm 0.1 \,{\mathrm{kJ}}\;{\mathrm{mol}}^{ - 1}$; $\Delta G_{{\mathrm{degenerate}},{\mathrm{AT}}} = - 1.75 \pm 0.12 \,{\mathrm{kJ}}\;{\mathrm{mol}}^{ - 1}$; $\Delta G_{cp} = - 3.91 \pm 0.47 \,{\mathrm{kJ}}\;{\mathrm{mol}}^{ - 1}$.; $\Delta G_{{\mathrm{elec}}} = - 8.18 \pm 0.06 \,{\mathrm{kJ}}\;{\mathrm{mol}}^{ - 1}$.

### Kinetic model of a gene transcription antenna

The model defines four different states of the TF in reference to its binding status to the DNA: (1) *C*_*F*_ corresponds to the pool of TF on the cell milieu; (2) *A*_*F*_ corresponds to the TF unbound and diffusing within the small cellular volume occupied by the DNA antenna; (3) *D*_*eg*_ corresponds to the TF bound to the cluster of degenerate consensus repeats that conform the antenna; (4) *SB* corresponds to the TF associated to the specific binding site. Obviously, in a real cell the TF will also have the opportunity to bind to other regions of the genome. However, from the point of view of the control of one gene, binding to other regions in the genome (other gene RRs controlled by the same TF and nonspecific binding to any DNA sequence) will simply decrease the overall availability of TF to be in the antenna, and thus it can be implicitly assumed as part of *C*_*F*_. The four states are kinetically connected according to the scheme depicted in Fig. 6c. In this scheme, the unbound protein can get in and out of the antenna space by simple diffusion. This transport is modeled kinetically through the rate constants k_1_ (onto the antenna) and k_−1_ (out of the antenna). k_−1_ is much larger than k_1_ due to purely entropic considerations (the cell volume is several orders of magnitude larger than the volume occupied by the antenna). Once in the antenna, the free TF (*A*_*F*_) can bind by simple 3D diffusion to any of the many DCB sites (together conforming *D*_*eg*_) via k_2_, and to SB via k_3_. The TF molecule can be released back to *A*_*F*_ from the bound states via k_−2_ and k_−3_, respectively. Finally, the TF can reach *SB* from *D*_*eg*_ or vice versa via one dimensional diffusion on the antenna (sliding), which in the model is represented by k_−4_ and k_4_. This kinetic scheme results in the following rate matrix:

7 $$\left[ {\begin{array}{*{20}{c}} { - {\boldsymbol{k}}_1} & {{\boldsymbol{k}}_{ - 1}} & 0 & 0 \\ {{\boldsymbol{k}}_1} & { - \left( {{\boldsymbol{k}}_{ - 1} + {\boldsymbol{k}}_2 + {\boldsymbol{k}}_3} \right)} & {{\boldsymbol{k}}_{ - 2}} & {{\boldsymbol{k}}_{ - 3}} \\ 0 & {{\boldsymbol{k}}_2} & { - \left( {{\boldsymbol{k}}_{ - 2} + {\boldsymbol{k}}_4} \right)} & {{\boldsymbol{k}}_{ - 4}} \\ 0 & {{\boldsymbol{k}}_3} & {{\boldsymbol{k}}_4} & { - \left( {{\boldsymbol{k}}_{ - 3} + {\boldsymbol{k}}_{ - 4}} \right)} \end{array}} \right]$$

For a given set of rate constants, this rate matrix can be solved as an eigenvalue problem using standard matrix calculus. The three nonzero eigenvalues provide the kinetic phases of the model and the eigenvectors provide the kinetic amplitudes (or the equilibrium populations for the zero eigenvalue) for a given set of initial conditions (i.e., initial populations). To illustrate the functioning of such an antenna system with conditions that are functionally relevant, we parameterized the rate matrix as: (a) assuming a concentration of TF in the cell of 1 nM and a diffusion coefficient for a protein inside a cell of $D = 3 \cdot 10^{ - 8}\,{{\mathrm{cm}}^2}{\mathrm{s}}^{ - 1}$. The rate constant to get into the antenna from the cell milieu (k_1_) was set to $10^6\,{\mathrm{M}}^{ - 1} {\mathrm{s}}^{ - 1} \cdot 10^{ - 9}\,{\mathrm{M}} = 10^{ - 3} {\mathrm{s}}^{ - 1}$. The rate constant to escape from the antenna (k_−1_) is defined by the ratio between the cell and antenna volumes (1000 μm^3^/0.033 μm^3^) to keep detailed balance, thus resulting on a rate of 30*s*^−1^. The antenna includes a *D*_*eg*_ composed of 900 degenerate consensus sites, each one of them with $K_{D,2} = 10^{ - 7}\,{\mathrm{M}}$; and a single specific binding site (*SB*) with *K*_*D*,3_ = 10^−8^ M. The dissociation rate constant from *SB* to *A*_*F*_ was set to $k_{ - 3} = 10^9\,{\mathrm{M}}^{ - 1}{\mathrm{s}}^{ - 1} \cdot K_{D,3} = 10\,{\mathrm{s}}^{ - 1}$ and the global dissociation constant from *D*_*eg*_ to A_F_ was set to $k_{ - 2} = 10^9\,{\mathrm{M}}^{ - 1}{\mathrm{s}}^{ - 1} \cdot K_{D,2} = 100\,{\mathrm{s}}^{ - 1}$. The rate constants for binding from *A*_*F*_ to *SB* and *D*_*eg*_ are directly set by detailed balance to $k_3 = 10^7\,{\mathrm{s}}^{ - 1}$ and $k_2 = 900 \cdot 10^7{\mathrm{s}}^{ - 1}$, respectively. Finally, *SB* and *D*_*eg*_ can interconvert by 1D sliding. Using a sliding motion of approximately 200 bp·ms^−1^ and a mean distance separation of 2.5 kbp between *SB* and any degenerate consensus site within *D*_*eg*_, we estimated the mean rate constant to slide onto *SB* as $k_4 = 80\,{\mathrm{s}}^{ - 1}$, and thus, by detailed balance the rate to slide off SB is $k_{ - 4} = 0.89 \,{\mathrm{s}}^{ - 1}$. Using these numbers, the three nonzero eigenvalues of the rate matrix (Eq. 7) are: $\lambda _3 = 2.7 \cdot 10^7\,{\mathrm{s}}^{ - 1};\lambda _2 = 91\,{\mathrm{s}}^{ - 1};\lambda _1 = 1.1 \cdot 10^{ - 3} \, {\mathrm{s}}^{ - 1}$. The equilibrium populations are *C*_*F*_ = 0.099, *A*_*F*_ = 3.3.10^−6^, *D*_*eg*_ = 0.891, and *SB* *=* 0.0099 (Fig. 6d). Analysis of the amplitudes (eigenvectors) indicates that the slowest nonzero eigenvalue (λ_1_) reflects the very slow re-equilibration between the cell milieu and the antenna. The fastest eigenvalue (λ_3_) reflects the extremely fast equilibration between the free and bound TF molecules within the antenna (the population of *A*_*F*_ is very small, and thus its equilibration is nearly instantaneous). Finally, the intermediate eigenvalue (λ_2_) includes all the kinetic flux between *SB* and *D*_*eg*_, and thus it indicates how much time it takes a TF molecule located within the antenna to find the specific binding site (slightly over 10 ms). The response time is thus very fast, even though the specific binding site occupancy is maintained at a minimum (about 1%). These results are incorporated onto the graphical representation of the β3 tubulin gene antenna of Fig. 6d.

### Sequence analysis of engrailed transcription antennas

Engrailed is thought to regulate the expression of at least 203 genes whether by itself or with the participation of other transcription factors^36^. For the analysis of transcription antennas we have chosen 39 of these genes whose regulation by Engrailed is well known and described in depth by the Society for Developmental Biology (Bethesda, MD, USA). We obtained the extended gene sequence (gene transcript region plus 2 Kb on both ends) and the gene coding sequence (CDS) for the 39 selected genes directly from the genomic database for *D. melanogaster* FlyBase [http://flybase.org/]. We defined the regulatory regions for each gene as all of the noncoding regions found in the extended gene sequence, namely corresponding to the 2Kb before (5′ UTR) and after (3′ UTR) plus all the introns. We used this definition to be as comprehensive as possible and following the most extended practice in eukaryotic genomic analyses, which consider all these noncoding sequences as an extensive regulatory network on the basis of their highly conserved patterns^59^. In genes with alternative splicing, we only considered the longest CDS for the analysis. The various alternative transcripts present in each gene were obtained from the Ensembl database [http://www.ensembl.org]. For each gene, we calculated the expected frequency of finding a given sequence motif (the consensus sequence TAATTA, or the two or three different fragments of 5 or 4 consecutive consensus bases) by happenstance in both the CDS and in the regulatory regions taking into account that the A/T content of the *Drosophila* genome is 57%^54^. Accordingly, the expected number of observations for the consensus sequence is $F_{{\mathrm{TAATTA}}} = (0.285)^6x \, ({\mathrm{bp}} - 5)$, where bp is the total number of bases in the gene sequence. To calculate the expected frequency of observation for the fragments of the consensus site and avoid over-counting, we calculate the probability of a site of 6 bases in which one (for the 5-bp consensus fragments or quintets) or two (for the 4-bp consensus fragments or quartets) is different from the consensus sequence as: $F_{{\mathrm{Quintet}}} = (1 - 0.285)x\left( {0.285} \right)^5x({\mathrm{bp}} - 5)$ and $F_{{\mathrm{Quartet}}} = (1 - 0.285)^2x(0.285)^4x({\mathrm{bp}} - 5)$. The number of actual observations was determined by running a 6-bp window over the entire gene sequence and over the CDS. The number of observations on the regulatory region is obtained as the total minus the CDS. The ratio between observed and expected indicates whether the sequence is overrepresented (for ratios higher than 1) or underrepresented (ratios below 1). The results for the 39 genes are given in Supplementary Table 4. In addition to the simple statistical analysis, we employed the statistical mechanical model for EngHD binding to DNA parameterized with our FCS experiments to calculate the landscape of binding energetics for the 39 genes along their entire gene extended sequence (sliding window of 6-bp), as well as the apparent dissociation constant for each possible nonoverlapping segment of 75-bp along the extended gene sequence and the overall dissociation constant for the entire sequence (given in Supplementary Table 3).

### Sequence analysis of genes not under engrailed control

Identifying genes that are demonstrated not to be under Engrailed control is far from trivial because, as key transcription factor in development, Engrailed controls ubiquitous genes involved in many fundamental morphogenesis, communication and signaling processes, including genes that encode for signal proteins, receptors, protein kinases, protein phosphatases, transcription factors, and cell adhesion proteins^36^. We thus looked into the Interactive Fly database https://www.sdbonline.org/sites/fly//aimain/3a-dtest.htm, which contains a large number of *D. melanogaster* genes with detailed functional annotations, and eliminated all genes that are involved in any of those processes mentioned above and/or that are known to be controlled by transcription factors with a similar consensus binding sequence (e.g., most homeoboxes). After applying this filter, we identified a group of 24 genes unlikely to be controlled by Engrailed based on their known function, but still with AT contents close to the 57% of the overall *Drosophila* genome.

### Protein expression and purification

The coding sequence of EngHD (PDB: 3HDD) containing a Cys residue at the C-terminal was ordered from TopGene Technologies cloned into the pBAT 4 vector^60^. Sequence identity was confirmed by DNA sequencing. Recombinant protein expression was performed in transformed BL21 (DE3) cells (Novagen) grown at 37 °C/220 rpm and inducing with 1 mM IPTG at an OD_600nm_ ~ 1 for 4 h. Cells were pelleted, lysed by multiple freeze-thaw cycles in lysis buffer (100 mM Tris-HCl, pH 8, 200 mM KCl, 1 mM EDTA, 2 mM CaCl_2_, 2 mM MgCl_2_, 50% glycerol, 2 mM TCEP-HCl, 1 mM PMFS), and centrifuged at 30,000 rpm for 1 h. The supernatant was purified by cation exchange chromatography using a SP Sepharose Fast Flow column (GE Healthcare). The equilibration buffer contained 25 mM Tris-HCl pH 7.5, 0.1 mM EDTA, 0.1 M NaCl and 2 mM TCEP-HCl. Elution was performed in the same buffer but increasing salt concentration in a gradient of NaCl concentration up to 0.5 M. Fractions containing EngHD were analyzed by PAGE-SDS and dialyzed against PBS. The resulting EngHD samples were subsequently concentrated using Amicon Ultra-15 3.000 NMWL, Da (Millipore) and quantified by Abs_280nm_ and PAGE-SDS.

### Fluorescent labeling for FCS experiments

EngHD was labeled with Alexa 488 c5-maleimide at the C-terminal following the protocol provided by the manufacturer (Molecular Probes). Briefly, 3 mg of EngHD were mixed drop by drop with 1 mg of dye to a final volume of 3 ml in PBS buffer in presence of 2 mM TCEP-HCl. The sample was incubated in total darkness for 2 h at room temperature or overnight at 4 °C, after which the reaction was stopped adding 2-Mercaptoethanol (SIGMA). The fluorescently labeled protein was purified by reverse phase chromatography on 0–95% water/acetonitrile gradient in the presence of 0.1% trifluoroacetic acid. A subsequent purification step to eliminate any leftover of fluorophore non-covalently to the protein was performed by ultrafiltration using Amicon Ultra-15 3.000 NMWL, Da (Millipore) canisters in the presence of 6 M urea. Sample purity was confirmed by MALDI-TOF mass spectrometry. The degree of labeling (~99%) was obtained as the molar ratio between fluorophore and protein determined by absorbance, comparing the Abs_490nm_ for the fluorophore and the Abs_280nm_ for the protein and applying a correction factor of 0.11 for the fluorophore’s contribution to Abs_280nm_ (Molecular Probes).

### Design of the DNA molecules

All the double stranded DNA molecules used in our experiments are based on the sequence of the β3 tubulin gene (Genbank ID: 37888), which is a natural gene from *Drosophila melanogaster* known to be regulated by Engrailed. Specifically, we choose a 150 bp region (nt 2525–2674, counting from exon 1) that contains only one high affinity (but no consensus) binding site (TAATTG) with a predicted affinity in the nM range that is ideal for FCS experiments. The designed DNA sequences contain the high affinity TAATTG site in their central position and include segments from the β3 tubulin gene sequence that expand in both directions to complete a total of 38, 75, or 150 bp for each strand. Versions of the 75 and 150 bp DNA molecules were produced as follows: (1) changes involving just the binding site were directly introduced replacing the “TAATTG” site by TAATTA, TAATTT, GAATTG, TATATA, or CGTGTT; (2) 75 and 150 bp molecules not containing homo-nucleotide clusters were designed maintaining the composition of the original gene and the high affinity binding site but randomly alternating all the other bases to minimize clustering; (3) the 150 bp DNA molecule with half of the sequence containing the original homo-nucleotide clustering and half without clustering was produced using the original 75 bp molecule as central region and using end extensions in both directions designed with a random sequence that minimizes homo-nucleotide clustering; (4) the DNA molecule with high G/C content was designed keeping the high affinity binding site and randomly adding nucleotides (with 0.82 probability for G or C and 0.18 for A or T and probability of 1 for G or C after A/T) to both ends up to the required 75 bp extension; (5) the 300 bp DNA molecule was designed starting at 5′ with a XhoI restriction site, followed by the 150 bp molecule with the original sequence (excluding the TAATTG site and the last three bases), a single TAATTG site, and finalizing with another copy of the same 150 bp segment plus a HindIII restriction site at the 3′ end; (6) the 600 bp DNA molecule was designed starting with a 5′ NcoI restriction site, followed by the 150 bp DNA molecule (without the TAATTG), the sequence AAAGACAAA as nucleotides 148 to 156, a single TAATTG site, two copies in tandem of the 150 bp segment (without the TAATTG), and finalizing with the sequence AAAGACAAA and a HindIII restriction site at the 3′ end. The sequences for all the DNA molecules used in our study are given in Supplementary Table 1. The DNA encompassing the sequence of the entire β3 tubulin gene corresponds to the annotated FlyBase gene entry βtub60d, corresponding to the chromosome region: 2 R 24,305,881 to 24,313,099. The gene was synthesized by Bio Basic [biobasic.com] and cloned into the vector pUC57 using HindIII and KpnI.

### DNA purification and hybridization

Single stranded DNA molecules corresponding to both strands of the 38–150 bp DNA molecules were ordered from Sigma. The ssDNA molecules were re-suspended in sterile MilliQ water and their concentration determined by Abs_260nm_. Hybridization of the pairs of complementary strands were carried out by performing a temperature ramp from 95 °C to 4 °C in 45 min on a thermocycler. dsDNAs were stored at −20 °C in aliquots. The longer DNA molecules (300 bp and 600 bp) were synthesized by TOP Gene Technologies and delivered cloned into the pBAT 4 vector^60^. Plasmids were transformed into DH5α cells (Invitrogene) and purified by Maxiprep (Quiagen). The 300 and 600 bp *ds*DNA fragments were obtained by enzymatic digestion (XhoI/HindIII for 300 bp DNA or NcoI/HindIII for 600 bp DNA, NEB) and isolated from a 2% agarose gel using the Wizard SV Gel and PCR Clean-Up System (Promega). The 7219 bp DNA was obtained by enzymatic digestion (HindIII-KpnI) of the full β3-tubulin gene cloned into the pUC57 vector (Bio Basic) and isolated from a 2% agarose gel using the Wizard SV Gel and PCR Clean-Up System (Promega). *ds*DNA bands were eluted in sterile MilliQ water and quantified by Abs_260nm_. These DNAs were re-hybridized and stored following the same protocol used for the shorter DNAs. As controls to determine the translational diffusion coefficient of the DNA molecules by FCS, we ordered 5′-fluorescently labeled (with Alexa 488) ssDNA of 33 and 75 bases from IBA-lifesciences and ATD bio, respectively. These ssDNA molecules were, hybridized with their complementary unlabeled strand and stored using the same protocol described above.

### Glass coverslips preparation

Glass coverslips 24 × 24 mm #1 (Menzel-Gläser) were immersed in a solution containing 1vol. acetone/1 vol. methanol/2 vol. water and incubated for 5 min, followed by sonication for 30 min. in a bath with a 1 M KOH solution, a thorough rinse with water and immersion in acetone for 5 min. Cleaned coverslips were incubated for 5 min. in Vectabond reagent (Vector labs) using a ratio of 0.02 ml reagent for each ml of acetone. This product chemically modifies the glass to form a highly adherent surface. After Vectabond treatment, the coverslips were rinsed with water and stored in water (for no more than 2 months) until pegylation. As first step in the pegylation procedure, the coverslips were dried and pressed glued to a press-to-seal silicone isolator (Molecular Probes) mask to create the wells. A solution of 0.2 mg/µL of NHS-PEG in 100 mM sodium borate at pH 8.5 was added to each well and incubated for 3 h. This solution was removed after the 3 h incubation and the wells were rinsed with abundant water. The entire process was performed at room temperature, the used reagents were spectroscopic grade and the water was passed through a 0.22 µm filter (Millipore). The pegylated coverslips were stored in a closed box at 277 K with a drop of water in each well to avoid evaporation and were used within 4–5 days of preparation.

### FCS sample preparation

FCS experiments were performed at 296–298 K with 30 µl of solution (10 mM Tris-HCl pH 7.5, 0.1 mM EDTA, 50 mM NaCl) prepared with a fixed EngHD concentration of 2 or 5 nM. The dependence on the ionic strength was studied using the same buffer but increasing the NaCl concentration (150 mM or 350 mM). A series of experiments at various *ds*DNA concentrations was performed for each DNA molecule and condition (salt concentration) to ensure coverage of the entire binding isotherm (from the pM to the mM range). To avoid sample evaporation during the measurement, the well was covered with parafilm.

### FCS instrument configuration

All measurements were carried out on a MicroTime 200 confocal microscope (PicoQuant). A small fraction (~70 μW) of the 485 nm light emitted from a pulsed diode laser (Model LDH-D-C; 40 MHz pulse repetition rate and 50 ps pulse width) was reflected by a 510 nm long-pass dichroic mirror (Chroma) onto the back of the microscope objective (UPlanSApo 60 × /1.2 numerical aperture Olympus water immersion) and focused to a limited spot in the sample droplet with a focal plane ~20 μm above the solvent–glass interface. Fluorescence emitted from the probe volume was collected by the objective, passed through the excitation dichroic, and spatially filtered (with a HQ510 filter Chroma and a 75 μm pinhole) before being filtered again (525/50, Chroma) onto one single photon avalanche photodiode (PicoQuant). The autocorrelation function of the detector counts was calculated using the SymphoTime software (PicoQuant).

### Confocal volume calibration

The confocal volume was routinely determined by the FCS method assuming a Gaussian excitation volume and using a 5 nM solution of Atto488 dye (ATTO-TEC) as reference with known diffusion coefficient^61^, and a suitable pinhole to avoid artifacts^62^. This dye was chosen because it has little triplet buildup and the same excitation wavelength than Alexa 488. The confocal volume was obtained from the fluorescence autocorrelation decay of Atto488 using the SymphoTime software (PicoQuant). The confocal volume was estimated to be between 1 and 1.6 femtoliters for all the experiments described in this work.

### Theoretical calculations of diffusion coefficients

Estimates of the diffusion coefficient for EngHD and for the EngHD-DNA complexes (assuming diffusion of the complex can be approximated by the diffusion of the dsDNA molecule alone given the small contribution expected for the non-rigid ~7 kDa EngHD) were obtained from theoretical calculations with the software Hydropro^63^ (for EngHD and all DNAs up to 150-bp, which is the limit for the rigid rod approximation given the DNA’s persistence length) and from experimental determination from the autocorrelation decay in FCS (for EngHD and *ds*DNAs of 33-bp and 75-bp labeled with Atto488). Agreement between estimates from both methods was excellent.

### Determination of the K_*D*_ from FCS experiments

Determination of the *K*_*D*_ for a given EngHD-DNA complex was carried out by globally fitting the autocorrelation function decays for a series of FCS experiments at varying concentration of DNA in which the decay for each experiment at a given DNA concentration was analyzed using equation:

8 $$G\left( t \right) = G_F\left( t \right) \times G_T\left( t \right) \times G_D\left( t \right)$$

In this equation, the contribution to the fluorescence autocorrelation function corresponding to the diffusion of labeled EngHD molecules (free and in complex with DNA) in and out of the confocal volume is represented by

9 $$G_D\left( t \right) =	 \frac{1}{{\langle N\rangle }}\left[\left( {1 + \frac{t}{{\tau _D^{{\mathrm{prot}}}}}} \right)^{ \!- 1}\left( {1 + \left( {\frac{{\omega _{xy}}}{{\omega _z}}} \right)^2\frac{t}{{\tau _D^{{\mathrm{prot}}}}}} \right)^{ \!- 1/2}\left( {\varepsilon \phi P_{{\mathrm{prot}}}} \right)^2 \right. \\ 	+ \left. \left( {1 + \frac{t}{{\tau _D^{{\mathrm{comp}}}}}} \right)^{ \!- 1}\left( {1 + \left( {\frac{{\omega _{xy}}}{{\omega _z}}} \right)^2\frac{t}{{\tau _D^{{\mathrm{comp}}}}}} \right)^{ \!- 1/2}\left( {\varepsilon \phi \left( {1 - P_{{\mathrm{prot}}}} \right)} \right)^2 \right]$$

where $\langle N^{ - 1}\rangle = ( {\langle N_{{\mathrm{prot}}}\rangle + \langle N_{{\mathrm{comp}}}\rangle } )^{ - 1}$ is the average number of molecules (combination of free and complexed EngHD), $\tau _D^{{\mathrm{prot}}} = \omega _{xy}/4D_{{\mathrm{prot}}}$ is the mean diffusion time for fluorescently labeled EngHD in free form, $\tau _D^{{\mathrm{comp}}} = \omega _{xy}/4D_{{\mathrm{DNA}}}$ is the mean diffusion time for the labeled DNA alone (i.e., assuming the same diffusion coefficient for DNA alone and protein-DNA complex, see above), $\varepsilon \phi$ is the dye brightness and

10 $$P_{{\mathrm{prot}}} = \frac{1}{{1 + K_{D}\left[ {{\mathrm{DNA}}} \right]}}$$

is the fraction of EngHD present in free form at each concentration of DNA. The confocal volume dimensions $\omega _{xy}$ and $\omega _{z}$ were determined using Atto488 as a reference of known diffusion coefficient^61^, as described above.

The component of the fluorescence autocorrelation function due to triplet buildup is obtained as

11 $$G_T\left( t \right) = \left( {1 + A_Te^{( - t/\tau _T)}} \right)$$

where $A_T = \frac{T}{{1 - T}}$ is the fraction of A488 molecules in the triplet state. And the contributions to the fluorescence autocorrelation function arising from the presence of fluorophore molecules not associated to EngHD are obtained as

12 $$G_F\left( t \right) = \left( {1 + A_Fe^{( - t/\tau _F)}} \right)$$

where $A_F = \frac{{Fr}}{{1 - Fr}}$ is the fraction of A488 molecules that are detached from EngHD.

Fluorescence autocorrelation decays were delimited in time to eliminate after-pulsing effects. The complete series of FCS decays for EngHD in association with a given DNA molecule (varying the DNA concentration) were fitted globally to Eqs. (8)–(12) in which the diffusion coefficient for the free protein and for the complex, the apparent *K*_*D*_ for binding, fraction of A488 molecules detached from EngHD, the dye brightness on the free protein, and dye brightness on the complex, are all global parameters. The diffusion coefficient for free EngHD and EngHD-DNA complex were bound to the values that we determined in independent FCS experiments and theoretically using Hydropro^63^. For EngHD we obtained a value of 122 μm^2^ s^−1^, in good agreement with the value of 131 μm^2^ s^−1^ estimated by Hydropro. For the longest, non-rigid, DNAS (300 and 600-bp) we obtained the bounded values for the diffusion coefficient from values previously reported by other authors^64^ and from independent FCS measurements performed in conditions of DNA saturation (100% complex). The non-linear fitting of the fluorescence correlation function to Eqs. (8)–(12) was performed using a custom-built MATLAB routine implemented with the lsqcurvefit function for least-squares optimization. Equations (8)–(10) assume a simple two-state model for the binding of EngHD to DNA (each EngHD molecule is either free or bound), which is a reasonable approximation for FCS binding experiments in which the protein is labeled, the much larger DNA molecule determines the overall diffusion coefficient of the complex, and the experiments are performed at DNA concentrations equal or larger than the protein concentration (to ensure that the probability of two EngHD molecules binding to the same DNA molecule is small). The fitting procedure was performed for each series of FCS experiments at different concentrations of a given DNA molecule (FCS titrations). FCS titrations were repeated three independent times, and the fit was performed for each experiment independently. The fits were carried out expressing the *K*_*D*_ in base 10 logarithms to ensure linear sampling and fit convergence. The global fit to each individual FCS titration rendered an estimate of the *K*_*D*_ and uncertainty (at a 95% confidence, or two standard deviations). The reported values are weighted averages of the *K*_*D*_ and uncertainty (at 95% confidence) from the multiple FCS titrations performed for each DNA molecule. The weighted mean *K*_*D*_, uncertainty, *K*_*D*_ estimated for each individual titration experiment and their statistical weight are given in Supplementary Table 2. The statistical weight for each FCS titration of a given molecule was defined as the inverse of the squared uncertainty obtained from the global fit to all the FCS curves at different DNA concentrations. The statistical weights for each experiment were divided by the sum of the inverse of squared uncertainties for the three experiments to ensure proper normalization.

### Comparison with in vivo ChIP-Seq profile

ChIP-Seq experiments on transgenic flies expressing en-GFP fusion proteins, and IP using an anti-GFP antibody were retrieved from the ENCODE Project database (experiment ENCSR952TDU) and profiled for EngHD binding in vitro using the structure-based statistical mechanical model described in this work. Optimal idr thresholded peaks (file ENCFF680AMJ, dm6 *D. melanogaster* last whole genome release in 2014) rendered a collection of 2226 DNA fragments (<500 bp) that were translated to FASTA. The sequences of the 2226 fragments were profiled for in vitro binding to EngHD with the statistical mechanical model. The overall dissociation constant (i.e., for the entire fragment) was calculated for each fragment.

### Reporting summary

Further information on research design is available in the Nature Research Reporting Summary linked to this article.

## Supplementary information

Supplementary Information

Peer Review

Reporting Summary

## Data Availability

All data and the gene expression plasmid containing the EngHD are available upon request. The sequence of all the DNA molecules used in this study are given as supplementary information (Supplementary Table 1). DNA molecules from 38 to 150 bp were produced by chemical synthesis and purchased directly from Sigma–Aldrich (Missouri). DNA molecules of 300 bp and 600 bp were synthesized and cloned into the pBAT 4 vector by Top Gene Technologies (Canada). The 7219 bp full β3 tubulin gene was synthesized and cloned into the pUC57 vector by Bio Basic (Canada). The raw data resulting in Figs. 1c, 2a–c, 4b, c, 5a-c, 6a, b, 7a, b and Supplementary Figs. 1–3 are provided as a Source Data File.

## Source data

Source Data

## Code availability

Analysis of the individual FCS curves, the global fitting of FCS curves obtained at different DNA concentrations for each DNA molecule to determine their experimental *K*_*D*_, the two versions of the statistical mechanical model for EngHD binding to DNA, routines for the global fitting of the experimental *K*_*D*_ values for all DNA molecules with the statistical mechanical models, and the kinetic model of transcription antenna have been performed using custom code for Matlab. All the Matlab scripts are available upon request to the corresponding author.
