# Supplementary material for: Eukaryotic transcription factors can track and control their target genes using DNA antennas
Source: Nat Commun. 2020 Jan 28;11:540. doi: 10.1038/s41467-019-14217-8 (PMC6987225; doi:10.1038/s41467-019-14217-8)
Supplement: Supplementary file 1 — Supplementary Information [file 41467_2019_14217_MOESM1_ESM.docx]

**Supplementary Information**

**Eukaryotic transcription factors can track and control their target genes using DNA antennas**

Castellanos *et al*.^1,2,3^

*^1^IMDEA Nanosciences, Faraday 9, Campus de Cantoblanco, Madrid, 28049, Spain*

*^2^National Biotechnology Center, Consejo Superior de Investigaciones Científicas, Darwin 3, Campus de Cantoblanco, Madrid, 28049, Spain*

*^3^Department of Bioengineering, School of Engineering, University of California, 95343 Merced, CA, USA.*

**
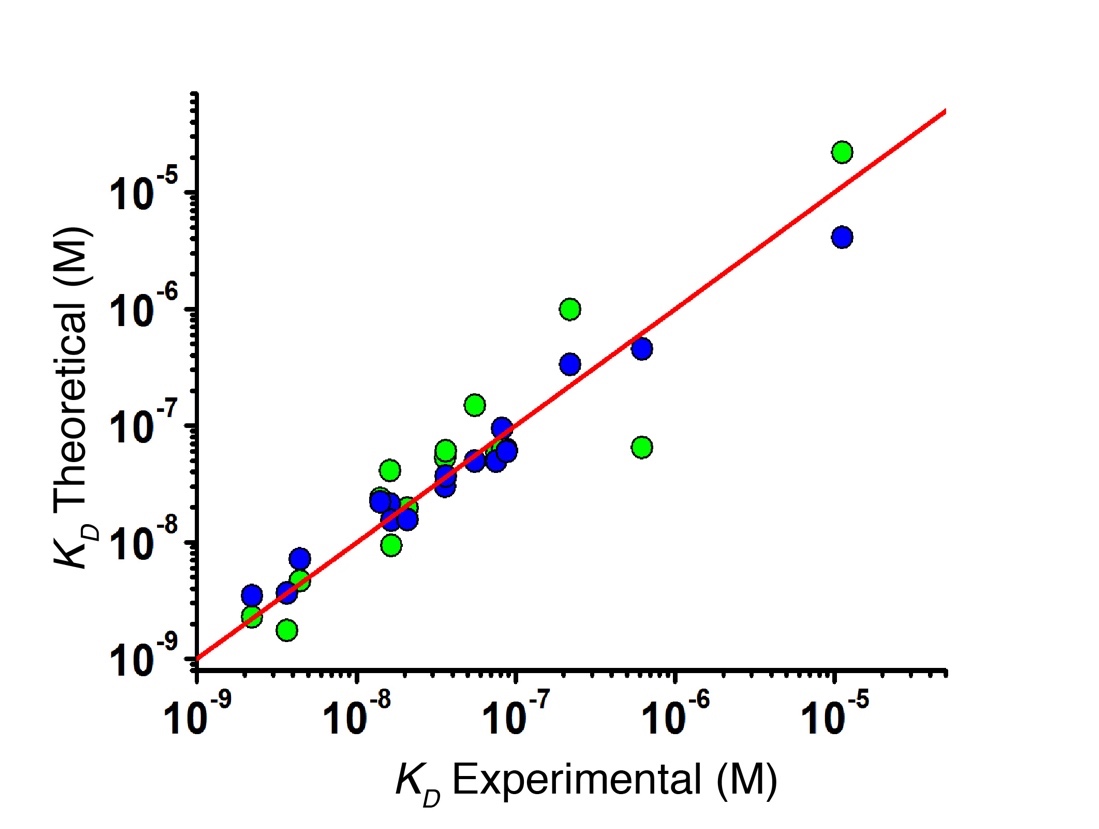
**

**Supplementary Figure 1.** Comparison between the binding affinity (*K_d_*) of EngHD for all of DNA molecules used in this work (tables s1 and s2) and the calculations with the globally optimized parameters for the two statistical mechanical models: the structure-based 4-parameter model (blue circles), and the position weight matrix model combined with electrostatic interactions, or PWM-Elec, (green circles). The red line represents a 1:1 correlation (i.e. perfect agreement between experiment and calculation). Parameter optimization was performed in log10 units to linearize the fitting procedure. The structure-based model calculations render a sum of least squares of 0.464 log 10 units for the structure-based model, and of 2.25 log10 units for the PWM-Elec model. That is, the structure-based model has 50-fold better agreement with the experimental data, which results in a probability smaller than 10^‑9^ for the statistically simpler model (PWM-Elec) to be comparable to the statistically complex model (structure-based) according to the Fisher test (SLS of 2.9 and 4 fitting parameters versus SLS of 145 and 1 fitting parameter).

**
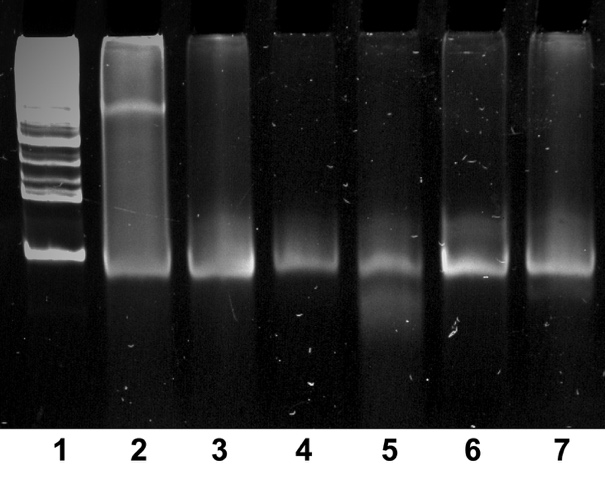
**

**Supplementary Figure 2.** Electrophoretic mobility in native conditions of the 75bp DNA molecules used in this study. The electrophoresis of all of the annealed dsDNA molecules was performed in a non-denaturing polyacrylamide (15%) gel. Lanes from the left to the right: 1) 100bp DNA ladder (Lonza); 2) 75bp DNA with high G/C content; 3) 75 bp original β3 tubulin gene fragment with the original TAATTG sequence in the specific binding site; 4) 75 bp original β3 tubulin gene fragment with GAATTG in the specific binding site; 5) 75 bp original β3 tubulin gene fragment with TAATTA in the specific binding site; 6) 75 bp original β3 tubulin gene fragment with TAATTT in the specific binding site; 7) 75 bp original β3 tubulin gene fragment with CGTGTT in the specific binding site. The DNA with high G/C content (lane 2) shows additional low mobility bands that reflect the formation of secondary structure.


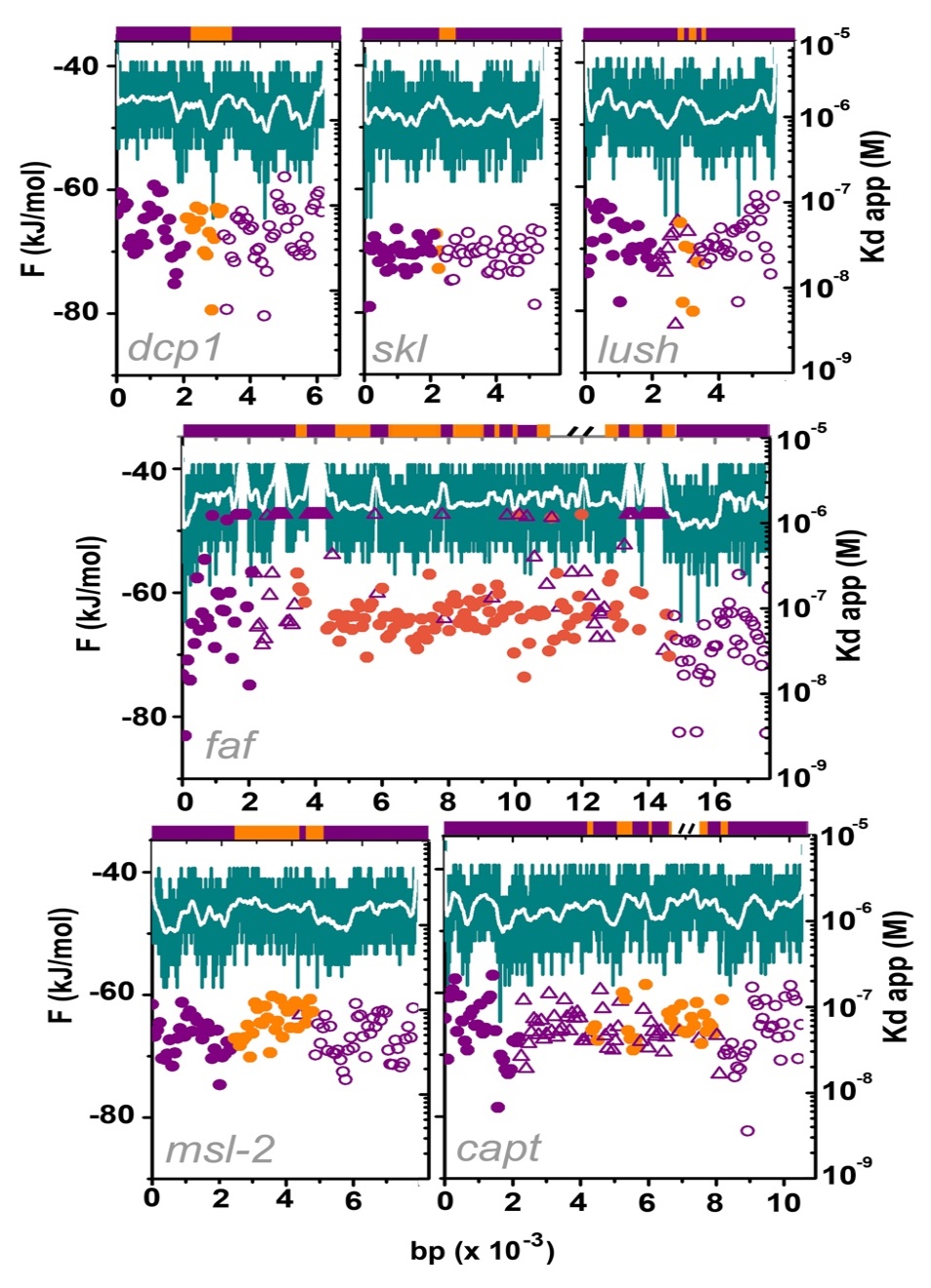


**Supplementary Figure 3.** Examples of EngHD binding profiles predicted by the statistical mechanical model for *D. melanogaster* genes (including 2 kbp before the 5’ UTR and 2 kbp after 3’ UTR) that are not expected to be under control by Engrailed. The gene organization is shown as a bar on top (exons in orange; 5’ UTR, 3’ UTR and introns in purple). Dissociation constants (right scales) follow the same color scheme with closed circles for 5’ UTR and exons, open circles for 3’ UTR and open triangles for introns. The binding free energy for the coding strand is shown in teal (left scales). The AT content for the six genes are (in the same order of the figure): 52%, 62.5%, 59%, 53%, 52%, 50%.

| **DNA molecule**  **(binding site)** | **DNA molecule**  **(flanking sequence)** | **Sequence (5’-3’)** |
| --- | --- | --- |
| 38 bp TAATTG | Original β3 tubulin gene sequence | AAAAGCCATCAATCTA**TAATTG**CAATCTAACTGTGCCT |
| 75 bp TAATTG |  | GCCCGAAATAAATCTGGCCGAAAAGCCATCAATCTA**TAATTG**CAATCTAACTGTGCCTCGGTTTATACCTCCAAC |
| 75 bp TAATTA |  | GCCCGAAATAAATCTGGCCGAAAAGCCATCAATCTA**TAATTA**CAATCTAACTGTGCCTCGGTTTATACCTCCAAC |
| 75 bp TAATTT |  | GCCCGAAATAAATCTGGCCGAAAAGCCATCAATCTA**TAATTT**CAATCTAACTGTGCCTCGGTTTATACCTCCAAC |
| 75 bp GAATTG |  | GCCCGAAATAAATCTGGCCGAAAAGCCATCAATCTA**GAATTG**CAATCTAACTGTGCCTCGGTTTATACCTCCAAC |
| 75 bp TATATA |  | GCCCGAAATAAATCTGGCCGAAAAGCCATCAATCTA**TATATA**CAATCTAACTGTGCCTCGGTTTATACCTCCAAC |
| 75 bp CGTGTT |  | GCCCGAAATAAATCTGGCCGAAAAGCCATCAATCTA**CGTGTT**CAATCTAACTGTGCCTCGGTTTATACCTCCAAC |
| 150 bp TAATTG |  | TTCTTAACCAAATTGAATTCTCGCCACCGATCGTGTTCGCCCGAAATAAATCTGGCCGAAAAGCCATCAATCTA**TAATTG**CAATCTAACTGTGCCTCGGTTTATACCTCCAACTGCCGTTCACCGAAAAGGCCGACAGTTTTCCGACAAA |
| 300 bp TAATTG |  | CTCGAGTTCTTAACCAAATTGAATTCTCGCCACCGATCGTGTTCGCCCGAAATAAATCTGGCCGAAAAGCCATCAATCTACAATCTAACTGTGCCTCGGTTTATACCTCCAACTGCCGTTCACCGAAAAGGCCGACAGTTTTCCGAC**TAATTG**TTCTTAACCAAATTGAATTCTCGCCACCGATCGTGTTCGCCCGAAATAAATCTGGCCGAAAAGCCATCAATCTACAATCTAACTGTGCCTCGGTTTATACCTCCAACTGCCGTTCACCGAAAAGGCCGACAGTTTTCCGACAAGCTT |
| 600 bp TAATTG |  | CCATGGTTCTTAACCAAATTGAATTCTCGCCACCGATCGTGTTCGCCCGAAATAAATCTGGCCGAAAAGCCATCAATCTACAATCTAACTGTGCCTCGGTTTATACCTCCAACTGCCGTTCACCGAAAAGGCCGACAGTTTTCCGACAAAGACAAATTCTTAACCAAATTGAATTCTCGCCACCGATCGTGTTCGCCCGAAATAAATCTGGCCGAAAAGCCATCAATCTACAATCTAACTGTGCCTCGGTTTATACCTCCAACTGCCGTTCACCGAAAAGGCCGACAGTTTTCCGAC**TAATTG**TTCTTAACCAAATTGAATTCTCGCCACCGATCGTGTTCGCCCGAAATAAATCTGGCCGAAAAGCCATCAATCTACAATCTAACTGTGCCTCGGTTTATACCTCCAACTGCCGTTCACCGAAAAGGCCGACAGTTTTCCGACTTCTTAACCAAATTGAATTCTCGCCACCGATCGTGTTCGCCCGAAATAAATCTGGCCGAAAAGCCATCAATCTACAATCTAACTGTGCCTCGGTTTATACCTCCAACTGCCGTTCACCGAAAAGGCCGACAGTTTTCCGACAAAGACAAAAAGCTT |
| 75 bp CGTGTT | Shuffled | GCTCGACATAGATCTGACTGACATGCTATCAGTCTA**CGTGTT**CAGTCTAGCTGTGCATCGATCTATACATCTAGC |
| 75 bp TAATTG | High G/C content | CCCCCTCCCACCCCTCCACCCCCTCCCCTCCCCCCC**TAATTG**CCCTCCCCTCCCCCACCCCCTCCCCCACCCCCT |
| 150 bp TAATTG | Shuffled | TACTCATCGATACTCAGTACTCGCGACGCATCGTGTACGCGCGACATACATCTCACTGATACGCGATCACTCTA**TAATTG**CACTCTATCTGTGCATCGCTCTATAGCTCTATCTGCAGTACACTGATATGACAGACAGTATGCTCACATA |
| 150 bp TAATTG | Half original/ Half Shuffled | TACTCATCGATACTCAGTACTCGCGACGCATCGTGTACGCCCGAAATAAATCTGGCCGAAAAGCCATCAATCTA**TAATTG**CAATCTAACTGTGCCTCGGTTTATACCTCCAACTGCAGTACACTGATATGACAGACAGTATGCTCACATA |

**Supplementary Table 1.** Sequences of the dsDNA molecules used in this work. The coding strand in the 5’ to 3’ sense is shown.

| **DNA molecule**  **(binding site)** | **-log10 *K_D_***  **weighted mean & uncertainty (95%)** | **-log10 *K_D_***  **single experiments (statistical weight)** | **DNA molecule**  **(flanking sequence)** |
| --- | --- | --- | --- |
| 38 bp TAATTG | 7.26 ± 0.13 | 7.36 (1.00)  7.03 (0.86)  7.36 (0.86) | Original β3 tubulin gene sequence |
| 75 bp TAATTG | 7.44 ± 0.06 | 7.38 (0.44)  7.39 (0.99)  7.52 (1.00) |  |
| 75 bp TAATTG  (150 mM) | 6.66 ± 0.45 | 6.91 (0.83)  5.98 (1.00)  7.20 (0.85) |  |
| 75 bp TAATTG  (350 mM) | 4.95 ± 0.91 | 4.06 (1.00)  6.50 (0.64)  4.67 (0.41) |  |
| 75 bp TAATTA | 8.43 ± 0.18 | 8.49 (0.15)  8.07 (0.89)  8.38 (0.93)  8.81 (1.00) |  |
| 75 bp TAATTT | 7.79 ± 0.10 | 7.66 (0.88)  8.25 (0.90)  7.64 (0.90)  7.90 (1.00) |  |
| 75 bp GAATTG | 7.12 ± 0.07 | 7.01 (0.78)  7.17 (1.00)  7.17 (0.79) |  |
| 75 bp TATATA | 7.44 ± 0.07 | 7.42 (1.00)  7.43 (0.49)  7.37 (0.53)  7.63 (0.99)  7.25 (0.68) |  |
| 75 bp CGTGTT | 7.06 ± 0.43 | 6.58 (1.00)  6.95 (0.45)  7.75 (0.77) |  |
| 150 bp TAATTG | 7.78 ± 0.10 | 7.66 (1.00)  7.64 (0.97)  7.81 (0.94)  8.01 (0.95) |  |
| 300 bp TAATTG | 8.35 ± 0.35 | 8.98 (0.61)  8.18 (0.81)  8.11 (1.00) |  |
| 600 bp TAATTG | 8.65 ± 0.18 | 8.78 (0.63)  8.91 (0.56)  8.43 (1.00) |  |
| 75 bp CGTGTT | 7.08 ± 0.25 | 7.12 (0.94)  7.33 (1.00)  6.66 (0.67) | Shuffled |
| 75 bp TAATTG | 6.21 ± 0.26 | 5.80 (0.57)  6.43 (0.69)  5.69 (0.55)  6.57 (1.00) | High G/C content |
| 150 bp TAATTG | 7.85 ± 0.36 | 7.51 (0.90)  7.58 (0.99)  8.42 (1.00) | Shuffled |
| 150 bp TAATTG | 7.68 ± 0.07 | 7.74 (0.82)  7.63 (1.00) | Half Original/ Half Shuffled |

**Supplementary Table 2.** Experimentally determined dissociation constants (*K_D_*) and their statistical uncertainty at 95% confidence (two standard deviations) shown in base 10 logarithm units. The second column provides the weighted mean and uncertainty. The third column shows the individual *K_D_* values from the global fits to each independent titration experiment and their statistical weight (calculated from the residuals of the global fit, see Methods). DNA molecules are named as in Supplementary table 1. Unless indicated, the experimental ionic strength is 50 mM.

| **Gene (ID)** | **Length (bp)*** | **Engrailed function** | ***K_D_* (pM)** |
| --- | --- | --- | --- |
| 18 wheeler (18w) | 9,422 | Activation | 150 |
| abdominal a (abd-A) | 26,836 |  | 42 |
| bagpipe (bap) | 5,570 |  | 250 |
| eagle (eg) | 12,898 |  | 81 |
| hedgehog (hh) | 17,738 |  | 91 |
| polyhomeotic distal (phd) | 11,560 |  | 150 |
| polyhomeotic proximal (php) | 16,162 |  | 920 |
| slit (sli) | 56,624 |  | 23 |
| thickveins (tkv) | 56,385 |  | 20 |
| ventral nervous system defective (vnd) | 20,031 |  | 82 |
| wingless (wg) | 13,107 |  | 95 |
| araucan (ara) | 19,347 | Repression | 51 |
| branchless (bnl) | 46,745 |  | 25 |
| **β-Tubulin at 60D (βtub60D)** | 11,219 |  | 164 |
| caudal (cad) | 16,405 |  | 91 |
| caupolican (caup) | 16,168 |  | 67 |
| cubitus interruptus (ci) | 13,332 |  | 53 |
| Connectin (Con) | 146,276 |  | 8 |
| Division abnormally delayed (dally) | 67,688 |  | 17 |
| Deformed (Dfd) | 14,593 |  | 71 |
| decapentaplegic (dpp) | 35,452 |  | 40 |
| Drop (Dr) | 12,859 |  | 85 |
| engrailed (en) | 8,205 |  | 18 |
| frazzled (fra) | 39,132 |  | 32 |
| frizzled (fz) | 98,297 |  | 11 |
| frizzled 2 (fz2) | 98,399 |  | 11 |
| hibris (hbs) | 34,739 |  | 30 |
| huckebein (hkb) | 5,632 |  | 355 |
| intermediate neuroblast defective (ind) | 5,126 |  | 242 |
| invected (inv) | 39,231 |  | 31 |
| knirps (kni) | 7,049 |  | 163 |
| knirps-like (knrl) | 27,519 |  | 39 |
| Neuroglian (Nrg) | 41,798 |  | 29 |
| patched (ptc) | 19,575 |  | 81 |
| rhomboid (rho) | 9,134 |  | 151 |
| scribbler (sbb) | 83,099 |  | 13 |
| sloppy paired 1 (slp1) | 5,425 |  | 237 |
| sloppy paired 2 (slp2) | 6,346 |  | 228 |
| Ultrabithorax (Ubx) | 81,804 |  | 14 |

**Supplementary Table 3.** Genes regulated by Engrailed used in the analysis of transcription antennas. The table includes the gene ID and length of the extended gene sequence (whole transcript plus 2 Kb on both 5’ and 3’ ends: “Gene Extended” in Flybase; [<http://flybase.org/>], an annotation indicating whether Engrailed acts as activator or repressor, and the overall dissociation constant (*K_d_*) calculated from the gene sequence by the statistical mechanical model.

| **Gene (ID)** | **Regulatory Regions (Observed/Expected)** | | | | | | **CDS (Observed/Expected)** | | | | | |
| --- | --- | --- | --- | --- | --- | --- | --- | --- | --- | --- | --- | --- |
|  | **TAATTA** | **TAATT** | **AATTA** | **TAAT** | **AATT** | **ATTA** | **TAATTA** | **TAATT** | **AATTA** | **TAAT** | **AATT** | **ATTA** |
| **18w** | 10/3 | 14/7 | 20/7 | 16/18 | 22/18 | 16/18 | 0/2 | 0/6 | 0/6 | 3/14 | 2/14 | 2/14 |
| **abd-A** | 24/13 | 64/34 | 58/34 | 79/85 | 172/85 | 66/85 | 0/1 | 0/2 | 0/2 | 1/6 | 3/6 | 1/6 |
| **bap** | 3/2 | 8/6 | 11/6 | 9/15 | 25/15 | 11/15 | 0/1 | 0/2 | 0/2 | 0/4 | 2/4 | 1/4 |
| **eg** | 14/6 | 19/16 | 15/16 | 41/40 | 63/40 | 49/40 | 0/1 | 0/2 | 0/2 | 0/4 | 0/4 | 1/4 |
| **hh** | 7/9 | 26/22 | 25/22 | 47/55 | 74/55 | 39/55 | 0/1 | 0/2 | 0/2 | 0/5 | 2/5 | 0/5 |
| **phd** | 6/4 | 9/9 | 9/9 | 22/23 | 23/23 | 13/23 | 0/2 | 0/6 | 2/6 | 3/16 | 6/16 | 6/16 |
| **php** | 6/6 | 20/15 | 19/15 | 26/38 | 70/38 | 22/38 | 0/3 | 0/6 | 2/6 | 3/16 | 3/16 | 4/16 |
| **sli** | 52/27 | 86/67 | 109/67 | 128/169 | 238/169 | 112/169 | 0/3 | 1/9 | 0/9 | 2/22 | 9/22 | 2/22 |
| **tkv** | 58/29 | 107/73 | 90/73 | 131/184 | 265/184 | 129/184 | 0/1 | 0/2 | 0/2 | 2/6 | 0/6 | 3/6 |
| **vnd** | 9/10 | 32/24 | 24/24 | 39/60 | 76/60 | 46/60 | 0/1 | 0/3 | 0/3 | 2/7 | 1/7 | 1/7 |
| **wg** | 10/6 | 20/16 | 25/16 | 26/39 | 58/39 | 33/39 | 0/1 | 0/2 | 0/2 | 1/5 | 0/5 | 2/5 |
| **ara** | 26/9 | 43/23 | 47/23 | 49/58 | 110/58 | 42/58 | 0/1 | 1/3 | 0/3 | 1/7 | 2/7 | 0/7 |
| **bnl** | 37/24 | 107/60 | 77/60 | 127/150 | 286/150 | 111/150 | 0/1 | 1/3 | 0/3 | 6/8 | 4/8 | 3/8 |
| **βtub60D** | 2/5 | 17/13 | 19/13 | 19/33 | 41/33 | 19/33 | 0/1 | 0/2 | 0/2 | 0/5 | 0/5 | 0/5 |
| **cad** | 7/8 | 26/20 | 29/20 | 36/51 | 60/51 | 51/51 | 0/1 | 0/2 | 0/2 | 0/4 | 2/4 | 1/4 |
| **caup** | 18/8 | 33/19 | 28/19 | 38/48 | 89/48 | 39/48 | 0/1 | 0/3 | 0/3 | 1/7 | 1/7 | 1/7 |
| **ci** | 26/5 | 23/12 | 28/12 | 43/31 | 72/31 | 44/31 | 0/2 | 6/6 | 10/6 | 15/14 | 17/14 | 13/14 |
| **Con** | 120/77 | 284/194 | 276/194 | 373/486 | 781/486 | 290/486 | 0/1 | 2/3 | 1/3 | 3/7 | 7/7 | 2/7 |
| **dally** | 69/35 | 134/88 | 116/88 | 145/222 | 356/222 | 120/222 | 0/1 | 0/3 | 0/3 | 4/6 | 4/6 | 0/6 |
| **Dfd** | 19/7 | 21/17 | 23/17 | 39/43 | 61/43 | 38/43 | 0/1 | 0/2 | 2/2 | 0/6 | 1/6 | 1/6 |
| **dpp** | 23/18 | 46/45 | 57/45 | 70/114 | 158/114 | 50/114 | 0/1 | 0/2 | 2/2 | 1/6 | 1/6 | 2/6 |
| **Dr** | 17/6 | 34/15 | 32/15 | 26/38 | 51/38 | 32/38 | 0/1 | 0/2 | 0/2 | 0/5 | 0/5 | 0/5 |
| **en** | 5/4 | 19/9 | 21/9 | 13/22 | 24/22 | 14/22 | 0/1 | 0/2 | 0/2 | 0/6 | 0/6 | 2/6 |
| **fra** | 28/19 | 70/46 | 72/46 | 76/117 | 188/117 | 86/117 | 1/2 | 0/6 | 1/6 | 5/15 | 5/15 | 11/15 |
| **fz** | 98/52 | 222/130 | 207/130 | 242/325 | 543/325 | 237/325 | 0/1 | 0/2 | 0/2 | 1/6 | 3/6 | 4/6 |
| **fz2** | 92/51 | 186/129 | 203/129 | 240/324 | 533/324 | 205/324 | 0/1 | 0/3 | 0/3 | 1/8 | 1/8 | 1/8 |
| **hbs** | 37/17 | 79/42 | 54/42 | 79/105 | 169/105 | 119/105 | 1/2 | 1/5 | 3/5 | 5/13 | 6/13 | 3/13 |
| **hkb** | 0/3 | 3/6 | 9/6 | 8/16 | 21/16 | 11/16 | 0/0 | 0/1 | 0/1 | 0/3 | 0/3 | 2/3 |
| **ind** | 4/2 | 10/6 | 14/6 | 16/14 | 24/14 | 4/14 | 0/1 | 0/1 | 0/1 | 0/3 | 2/3 | 3/3 |
| **inv** | 34/20 | 85/50 | 54/50 | 97/126 | 189/126 | 79/126 | 0/1 | 0/2 | 0/2 | 1/6 | 2/6 | 0/6 |
| **kni** | 7/3 | 7/8 | 17/8 | 13/19 | 26/19 | 12/19 | 0/1 | 0/2 | 0/2 | 0/4 | 0/4 | 1/4 |
| **knrl** | 24/14 | 66/34 | 64/34 | 68/86 | 152/86 | 57/86 | 0/1 | 0/3 | 1/3 | 3/7 | 2/7 | 1/7 |
| **Nrg** | 31/20 | 78/51 | 69/51 | 97/128 | 190/128 | 94/128 | 0/2 | 1/5 | 1/5 | 3/13 | 8/13 | 6/13 |
| **ptc** | 8/8 | 15/21 | 25/21 | 35/53 | 69/53 | 34/53 | 0/2 | 0/5 | 1/5 | 4/13 | 4/13 | 1/13 |
| **rho** | 5/4 | 20/11 | 21/11 | 21/27 | 46/27 | 14/27 | 0/1 | 0/1 | 0/1 | 0/4 | 1/4 | 1/4 |
| **sbb** | 85/41 | 137/102 | 139/102 | 192/257 | 442/257 | 177/257 | 0/4 | 0/9 | 2/9 | 7/24 | 11/24 | 2/24 |
| **slp1** | 5/2 | 5/6 | 8/6 | 12/15 | 23/15 | 7/15 | 0/1 | 0/1 | 0/1 | 0/3 | 1/3 | 0/3 |
| **slp2** | 2/3 | 10/7 | 18/7 | 17/17 | 23/17 | 16/17 | 0/1 | 1/2 | 0/2 | 1/5 | 2/5 | 3/5 |
| **Ubx** | 60/43 | 180/108 | 186/108 | 192/272 | 477/272 | 181/272 | 0/1 | 0/2 | 0/2 | 2/4 | 0/4 | 0/4 |
| **Ratio** | 1.75 | 1.52 | 1.50 | 0.75 | 1.6 | 0.69 | 0.04 | 0.11 | 0.22 | 0.26 | 0.36 | 0.27 |

**Supplementary Table 4.** Binding sequence and degenerated sequences observation in Engrailed regulated genes. Probability for expected (Exp.) T/A motifs was calculated as described in Material and Methods section and compared with observed (Obs.) frequency in both Regulatory Regions (UTRs and Introns) and Coding regions (CDS). Sextet corresponds to the consensus binding sequence “TAATTA”. Quintets and Quartets are all possible sequences which bases follow the same order than in the Binding Sequence, flanked for any base not included in the sextet. In red, the observed values when they are higher than the expected ones.
