# Supplementary material for: Eukaryotic transcription factors can track and control their target genes using DNA antennas
Source: Nat Commun. 2020 Jan 28;11:540. doi: 10.1038/s41467-019-14217-8 (PMC6987225; doi:10.1038/s41467-019-14217-8)
Supplement: Supplementary file 2 — Peer Review [file 41467_2019_14217_MOESM2_ESM.pdf]

Reviewers' comments:

Reviewer #1 (Remarks to the Author):

The manuscript addresses fundamental questions of interactions between proteins and DNA with implications to the mechanism by which the DNA target site is recognized. The main finding of this paper is the existence of flanking DNA sequences in the periphery of the target site that increase the affinity of the protein close to the relevant region in the genome. The study is performed for EnHD protein. This idea of "antenna" effect for DNA search (or the existence of a funneled landscape was proposed in the past and no evidence was found for that. The supports provided in the current study are only partially convincing.

Specific comments:
1. It was found that the A and T content are sufficient to increase the affinity of the protein to DNA and their clustering has little effect on the "antenna effect". How can one validate that this is because the consensus binding site for EnHD is rich with A & T? Perhaps, this is related to the conformation of DNA or its rigidity that serve as an "antenna"?

2. The presented statistical mechanics model is very simple and might lack generality to other systems. For example, why two terms are required for cooperativity (both cp5 and cp6). Why non-specificity is modeled for A and T, can't G and C also interact non-specifically. Overall, this model includes 8 free-parameters so their predictive power is limited.

3. The authors used single molecules measurements (FSC technique) to measure free energy for binding. There are more conventional ways to measure DG for binding that should be used to assess the new methodology.

4. The authors argue that the "antenna" works by increasing the frustration for protein-DNA binding and find this reminiscent to protein folding. However, in protein folding, the frustration should be minimal. In the current study, one gets the impression that ruggedness (frustration) is advantageous however, it may have a strong effect of slowing down kinetics. Improving the affinity of protein-DNA is just one aspect but kinetics (and 1D diffusion) might be slowed down dramatically by the frustration.

5. Figures 6 and 7: how come the protein has lower free energy to non-specific binding sites than to the consensus site. This is counterintuitive. Is there a meaning then to a specific binding site? The meaning is that the energy landscape for target site is anti-funnel.

6. Figure 7: The minimum to the left of the consensus site show a depletion of A and T content. Also does one expect a flanking region to be so long (hundreds of bp)?

7. It is argued that the DNA diffuse with coefficients that decrease proportionally to their size, indicating that they behave as rigid dsDNA. How a 600 bp DNA can be rigid? Why this excludes possibility of more compact DNA conformation?

Reviewer #2 (Remarks to the Author):

In their manuscript, Castellanos and Munoz investigate the DNA binding properties and specificity of the Drosophila TF Engrailed (En). First, they employ FCS to measure the binding affinity of En to several DNA sequences (75bp) from an intron sequence from the b-tubulin 3 gene. They find that En binds not only to its target site, but also to degenerate sites embedded within this DNA sequence, and that these interactions depend on the salt concentration. Based on these results, as well as the Xtal structure of the En DBD with DNA, they parametrize a statistical mechanics model for En DNA binding.

Using this model, they then investigate the DNA sequences around En binding sites, which reveals large numbers of potential degenerate binding sites. Thus, the authors propose that En can interact with DNA either nonspecifically, can bind to the consensus site, or bind to the degenerate sites. They then posit that the number of degenerate sites may act as antennae that direct En binding to its target sites in vivo. By sequence analysis, the authors show that degenerate sites are enriched at non-coding regions at several genes, and less enriched over coding regions. Using a kinetic model, they indicate how these degenerate sites may direct TFs to their target site, i.e. act as antennae.

This manuscript conveys a potentially interesting hypothesis, in that the DNA sequences surrounding promoters and enhancers are enriched with degenerate sequences for the relevant TFs that regulate said genes (named "antennae" by the authors). While this is an interesting hypothesis and worthwhile testing, I am however not convinced that the experimental data provided in this manuscript supports this hypothesis at this point. I thus think that this study is too premature to be published in Nature Communication. Comments follow below:

1. The authors make a case that the Antenna model is general. They however only provide data for En. I am thus not convinced that this is a general mechanism.
First, as the authors state on line 253 that Drosophila genome has a high AT content in intergenic regions, whereas this drops in coding regions. Purely random base arrangement might thus generate the proposed accumulation of En sites outside all genes (but not specifically for En).
Conversely, the Antenna model would not hold for TFs with a high GC content in their recognition sequence. To make a strong statement about a general mechanism, an "Antenna" sequence arrangement has to be shown for multiple, diverse TFs.

2. As there exists ample data on En binding sites within the Drosophila genome, e.g. by ChIP or DNAseI footprinting etc.
The authors write in the discussion that such data provides "too many hits and little correlation occupancy and gene expression levels". This is a very strong statement and should be supported by more evidence.
In contrary, the authors should show that their statistical model recapitulates obtained in vivo binding profiles.

3. The statistical model contains > 8 parameters, and is parameterized using only a 6 binding experiments. It is not clear to me how accurate / robust this model is. Moreover, the description of the parameters in Fig. 3b is unclear (circles, boxes etc.). It would be more informative to provide the actual parameters in a table, including parameter confidence intervals, and a parameter sensitivity analysis.
Then the model could also be validated with the native ChIP profiles.

4. The authors completely ignore the contribution of chromatin. The Drosophila genome is packed into chromatin which modulates motif accessibility genome wide. Nucleosome depleted regions around promoters however enable local TF binding. Chromatin thus may be a key driving factor for TF site selection. The authors should discuss this.

5. "the binding free energy landscape around the cognate site is rugged, resulting in binding competition"
I am not sure what this sentence means, this should be better explained.

Reviewer #3 (Remarks to the Author):

This paper on the biophysics of TF-DNA interaction explores the hypothesis that several weak binding sites for a transcription factor (TF) in the vicinity of a strong site may serve the special purpose of attracting and trapping the TF molecules to that locus, thereby increasing the effective local concentration of the TF. The authors draw an analogy of this phenomenon to an antenna. The study is performed mainly in the context of the TF Engrailed (EN) binding to the regulatory region of a particular gene (β3-tubulin) in Drosophila. The main contributions of the work are (a) use of fluorescence correlation spectroscopy (FCS) to measure dissociation constant (Kd) values for several binding sites for EN, (b) using these Kd data to fit a statistical mechanical model of TF binding to a longer DNA sequence, and (c) use of such a model on the regulatory region of the β3-tubulin gene to claim that the region's total affinity for the TF is completely dominated by contributions from weak sites, which play the important role of increasing the local TF concentration for the strong site in the region.

Overall, the paper presents a very interesting technical study of a phenomenon that others have written about as well, including at the level of biophysical models. The authors study an important topic, and employ interesting novel approaches to the problem. The main claim made needs to be made more often, or at least examined more often, than it is today. At the same time, I have a few main concerns and several technical questions that I write below.

General comments:
1) I do not think the observation of the 'antenna' has to be made by first introducing binding data from a less-used approach and a new computational model. Using a simple position weight matrix model of Engrailed binding (which is available), one could probably have reached similar conclusions, at least qualitatively, perhaps even quantitatively.
2) The main claim -- of weak binding sites enhancing local TF concentrations – has been made previously, e.g., in the relatively recent work of Boris Adryan and Sridhar Hannenhalli. I believe the work presented by the authors here is more quantitatively detailed, to their credit, but the claim is not novel.
3) I am not convinced that the extension of the model's predictions, shown to fit observations on sequences of length up to 600 bp, necessarily generalizes to sequences of length ~10 Kbp, and important step in the authors' demonstration of the antenna concept.
4) The narrative approach of hiding nearly all modeling details in the main text (and presenting them instead in Supplement only) does not quite work in this paper because the modeling is a key aspect of the work and without clarity on how the model works, it is hard to interpret its predictions and findings.

Specific comments:

"decreases in affinity that were proportional to their divergence from the consensus". How do the observed changes in affinity track with predictions from say a PWM model?

"key implication of these results is that EngHD is promiscuous in its specific binding to DNA." How is this interpretation reached? Please clarify.

"include specific interactions with each consensus base, plus two cooperative terms that kick in when the binding site contains the first 5 bases or the entire consensus site. For simplicity, we exploited structural similarity to group consensus interactions in four types: T1 + T5, A2 + A3, T4, and A6." The model needs better explanation. If one cannot understand even the basic ideas without the Supplementary Methods, it is hard to appreciate the work. Related specific questions:
1) How was the value of 510-4 obtained for w_0 ?
2) The model (Eqn 8 in supplement) is based on knowledge of the 3D structure of the protein bound to its consensus site, and assumes, by and large, that the sequence-dependent binding energies are dictated by the (position-specific) nucleotides seen in this consensus site. This key assumption has not been justified. For instance, the motifs for Engrailed at FlyFactorSurvey, based on Bacterial 1-hybrid

technology, suggest that the last two positions allow ‘G’, but ‘G’ at these positions may not be weak interactions.
3) No discussion of parameter estimation of the statistical mechanical model of Eqn 8 is provided. How many sequences are fit to? How reliable are these parameter estimates (reported in Figure 3B)? How good is the fit?
4) How is the partition function calculated for longer sequences that may harbor overlapping binding sites? How is the exponential time complexity of the calculation addressed?
5) Is it true, as Equation 6 seems to suggest, that only microstates with at most one site bound to the DNA molecule are considered? Previous models (e.g., PMID 19956545) allow for microstates with multiple bound sites. Does it make a difference, especially since the focus here is on DNA sequences with clusters of weak sites?

“EngHD engages in three different types of DNA binding, the classical specific (consensus) and non-specific binding modes, plus a new mode consisting of promiscuous specific binding to degenerate versions of the consensus sequence.” The B1H motif for EN (Fly Factor Survey) also suggests that the TF can bind promiscuously to mismatched versions of the consensus sequence. Please clarify what is the new finding using the FSC data and stat mech model.

“Contributions from flanking DCB could in fact explain why deviations from the consensus sequence on the SB site produce only minor affinity decreases. For example, replacement of A6 by T involves the loss of 4.8 kJ/mol in binding free energy, equivalent to a seven-fold drop in affinity, but the KD increases by only 4-fold (m2 vs. m1 in fig. 4b).” Yes, but this specific example could be more simply explained by the trained model being inaccurate. I was under the impression that the model was trained on all available affinities (not just for the consensus sequence). Is this not correct?

“The statistical mechanical model does indeed explain these apparent discrepancies as arising from rugged binding free energy landscapes with many local minima corresponding to DCB sites (fig. 4c).” How? I don’t see how Figure 4C explains the apparent discrepancies.

“Experiments with m3 showed a marked decrease in binding affinity with KD of ~0.7·10-6 M, or a 21-fold decrease relative to m2 (fig. 4b).” This is interesting to see, from experimental results. It would be nice to know how the computational estimates of binding strength would have compared (between m2 and m3) if the estimates were made by scoring for presence of known PWM of EN (From Fly Factor Survey), using sum (exp (LLR(site)) over the entire sequence.

An important claim made by the authors is “the effects from the flanking sequence arise from DCB instead of from sequence-independent base clustering”. I agree with this, in light of the data and the model. But it seems to me that this is confirming what is generally believed to be the case. When prior literature talks about facilitated diffusion, doesn’t it assume that the facilitating sites invoke sequence-dependent binding? I may be mistaken about this, but that was my understanding.

The most significant idea/claim presented by the authors is “DCB transforms the β3-tubulin RR onto a potent attractor for EngHD molecules, which could accordingly operate as a transcription antenna to ensure local availability of the transcription factor as well as low occupancy of the specific site.” This is based on “a 500-fold affinity increase for the entire β3-tubulin gene, relative to its high affinity SB site alone (fig. 5b)”, as predicted by their model. However, this extrapolation is not justified. It is entirely possible for a model to operate up to a scale of 600 bp but not extend to a scale of 10000 bp. For instance, models of enhancer function that work on ~1 Kbp scale do not work at the ~10 Kbp scale.

The ‘gcPBM’ technology provides binding intensity measurements of hundreds of thousands of binding sites in their genomic context. Such data are available for several TFs. Could similar results have been obtained (as those presented here) by analyzing the gcPBM data using PWM motifs? Such an approach would not require the hand-crafting of the stat mech model presented here.

.

Reviewer #4 (Remarks to the Author):

This is a paper that combines biophysical experiments and theory to analyze the mechanisms of transcription factors recognizing their specific binding sites in eukaryotic cells. The subject is very important, and authors made a lot of work to clarify the very complex nature of these phenomena. There are several interesting ideas and results, and I like this work. I think the paper is potentially publishable but authors have to improve in many cases and many questions must be answered.
My specific comments are:
1) Generally, it is implicitly assumed that the transcription factors binding to their targets in cell is an equilibrium process. This is clearly not correct. So the consideration of affinities is important, but it is not enough to explain the dynamics of transcription factors binding. Authors should emphasize this.
2) There is a statement in the Introduction that for prokaryotic TF the targets are found by 1D sliding. This is clearly not correct statement. It is combination of sliding, binding from the bulk solution and intersegment transfer, and maybe other processes. See, e g., experimental and theoretical work in Nucl. Acid Res. 42, 7039 (2014), J.Phys. Chem. B 117, 12695 (2013).
3) The statistical mechanical model (as well as the kinetic model) must be explained better. It is a very phenomenological at this point. It has 8 parameters, as far as I can understand. But how many experimental data have been used to fit the data? 3 points in Fig. 2b is not enough. I think it is an overstatement (page 3) that the model captures the fundamental properties in the system. What things calculates the kinetic model?
4) page 4 - what is the "local base clusters"? It is not clear to me. Actually, authors criticize the theory due to Lukatsky and coworkers, but if you look at other papers of Lukatsky (not referred here) then the findings of this work actually agree. Lukatsky argued that the increase effective affinity to the specific sites is due to effective interactions from the flanking regions - exactly what is found in this work. I recommend to modify the discussion of these issues.
5) The effect of semi-specific sites and the role of the flanking regions on dynamics of TF have been explored theoretically, and authors seems unaware of this. Look at J. Chem. Phys. 143, 245101 (2015), where the dynamics of finding the target was analyzed using the analytically solvable model. Also see J. Phys. Chem. B 119, , 12410 (2015). Many similar results are found in these papers. Some discussion of these connections might be useful.
6) The picture proposed by the authors (DNA antennas) can work if the interactions in the flanking regions is not too strong and not too weak. So there is some optimality in the number and the strength of the degenerate sites. These degenerate sites must be similar but not too similar or not too dissimilar. It should be emphasized.

Point by point response to Reviewer's comments:

Reviewer 1

The manuscript addresses fundamental questions of interactions between proteins and DNA with implications to the mechanism by which the DNA target site is recognized. The main finding of this paper is the existence of flanking DNA sequences in the periphery of the target site that increase the affinity of the protein close to the relevant region in the genome. The study is performed for EnHD protein. This idea of "antenna" effect for DNA search (or the existence of a funneled landscape was proposed in the past and no evidence was found for that. The supports provided in the current study are only partially convincing.

As reviewer 1 indicates, the major finding of our work is the discovery, characterization (via biophysical experiments and bioinformatic analysis) and rationalization (via theoretical modeling) of a third binding mode on eukaryotic transcription factors (and Engrailed in particular) that combines the promiscuous DNA binding properties of its homeodomain with the specific organization of long tracts of degenerate consensus binding sites found in the regulatory regions of genes under Engrailed control to produce an efficient gene tracking mechanism. The mechanism that we find nicely explains several outstanding questions of eukaryotic gene expression at the molecular level. Reviewer 1 is absolutely correct that the idea that a special molecular mechanism beyond the interplay between specific and non-specific binding is needed to explain how eukaryotic TFs efficiently track and control their target genes is a long standing one. It is also absolutely true that, along those lines, many related mechanisms have been proposed over the years, which have all proven difficult to demonstrate experimentally. We think that one of the reasons for such gap has been a disconnect between molecular biologists and biophysicists working in this area, and an excessive focus on rationalizing all of the existing binding and bioinformatic data in terms of one or the other canonical binding modes: specific consensus binding and non-specific, sequence-independent, DNA binding/sliding.

The focus of molecular biologists has been on profiling the DNA sequences recognized by transcription factors by obtaining massive amounts of binding data using functional genomics assays (ChIP-seq,…) and high-throughput binding assays (SELEX, B1H, MITOMI,…). These methods are very powerful because they scan vast DNA sequence spaces, but are designed to only select the highest affinity binders, and are not truly quantitative. Therefore, the data that emerges from these methods are **consensus binding profiles** for each transcription factor (or their binding logos) that reflect their most favorable binding target (specific binding) and do not provide quantitative information of the actual binding affinity: a positive hit simply implies that the affinity is above the experimental threshold of the assay. Our understanding of the interactions associated to specific binding comes from structural studies of protein-DNA complexes and conventional *in vitro* low resolution binding assays that have traditionally used short artificial DNA sequences as model templates (thus missing the biological context), and which are analyzed with simple binding models that do not contemplate multisite binding and/or a broad distribution of affinities. In a parallel front, biophysical researchers, including theoretical, computational and experimental groups, and starting with the seminal work by von Hippel and coworkers, have focused on **non-specific binding/sliding**. In this context, several

theoretical analyses have recently entertained ideas of strengthened non-specific binding to the DNA regions flanking the functional target sites in eukaryotic systems. However, as reviewer 1 correctly states (and the other reviewers agree on, although expressed with variate emphasis), nobody before us has characterized experimentally the promiscuous binding of eukaryotic TFs to the DNA regions surrounding their functional target sites, nor it has been determined how much such semi-specific promiscuous binding contributes to the overall affinity of the TF, or how it interplays with the two canonical binding modes. We appreciate reviewer 1's understanding of the novelty and importance of our work.

Reviewer 1 also indicates that she/he is only partially convinced in light of the information presented in our original manuscript, and raises several issues that she/he deems necessary to be addressed to strengthen our work. We have taken reviewer 1's comments very seriously and have made multiple additions/changes to the manuscript that we believe fully address the stated concerns and should satisfy reviewer 1. The result is a much clearer and stronger study. Our point by point responses are given below.

Specific comments:

1. It was found that the A and T content are sufficient to increase the affinity of the protein to DNA and their clustering has little effect on the "antenna effect". How can one validate that this is because the consensus binding site for EnHD is rich with A & T? Perhaps, this is related to the conformation of DNA or its rigidity that serve as an "antenna"?

Reviewer 1 is correct that one of the observations of our study is that EnHD binds AT rich DNA with higher affinity than GC rich sequences. This is a type of non-consensus, but sequence-dependent (biologically significant), binding mode that we identify. But, it is just one aspect of the much richer binding patterns that we have discovered. In general, we find that EnHD binds DNA with a ladder of affinities, in which the full consensus has the highest, then the consensus core tetrad with non-consensus A/T in positions 1 and/or 6, then the same consensus tetrad with G/C in positions 1 and/or 6, then any site with incomplete consensus in the core tetrad (and within them, better for those rich in A/T versus G/C), then followed by full non-consensus but A/T rich sequences, and finally sequences that are G/C rich with the lowest affinity. All of these possible interactions, and their specific contributions, emerge directly from our experimental data and theoretical analysis, which demonstrate that all these binding nuances are present and contribute to the overall binding profile of EnHD (some with stronger and others with weaker overall contribution).

Therefore, it is definitely not just about being AT rich, and it is not about the accumulation of clustered base repeats (another type of non-consensus binding behavior proposed by Lukatsky and coworkers that does not seem to contribute significantly to EnHD's promiscuous binding). In reference to the overall preference for A/T, we agree with reviewer 1 that this is likely due to structural factors characteristic of these regions (narrower minor groove/more exposed major groove and increased flexibility). In the revised manuscript, we have included a full description of the statistical mechanical model (in response to comments below and comments also made by other reviewers) where we point to structural factors as potential origin for the overall preference for A/T even without any consensus agreement. We also describe in more detail all other interactions and their thermodynamic/structural justification.

Finally, whereas the preference for A/T versus G/C of EnHD is interesting in its own right, it definitely does not suffice to produce the type of transcription antenna mechanism that we propose, as it is not sufficiently specific to guarantee targeting the TF to the proper regions of the genome: many genome regions are likely to be A/T rich for a variety of reasons not related to EnHD binding. It is the full spectrum of binding affinities that we describe above what enables the transcription antenna mechanism and its easy implementation by patterning the sequence of the DNA regions with gene regulatory roles.

2. The presented statistical mechanics model is very simple and might lack generality to other systems. For example, why two terms are required for cooperativity (both cp5 and cp6). Why non-specificity is modeled for A and T, can't G and C also interact non-specifically. Overall, this model includes 8 free-parameters so their predictive power is limited.

This comment includes several issues that we address separately.

2.1) We discover and characterize the promiscuous binding of eukaryotic transcription factors to DNA and its utilization as transcription antennas in the model system EnHD. We believe that the phenomenon is general, as many of the properties of EnHD's binding to DNA are shared by most eukaryotic transcription factors. Particularly, the phenomenon should be directly extensible to all homeobox TFs, as their DNA binding domains have the same structure and binding profile of EnHD (most homeobox consensus sites are combinations of As and Ts). But, it is likely to be even more general given that all eukaryotic TF have very short recognition sequences (6-8 bp) that will necessarily result in promiscuous binding. Having said that, **the particular ladder of affinities for each TF will depend on the specifics of the consensus binding sequence recognized by that TF, the structure of the DNA binding domain (homeobox, HLH, zinc-finger) and also its aminoacid sequence (which will encode for base-specific recognition).**

Therefore, the tracts of sequence motifs conforming the transcription antennas of different TFs are likely to differ. The statistical mechanical model developed for this work targets EnHD and its parameters are specific for EnHD (determined from EnHD binding data). However, the same statistical mechanical model can be applied to other transcription factors by simply changing the parameters to properly reflect the interactions with DNA of the new protein. For example, Vismay is another *Drosophila* TF homeobox that binds to the consensus sequence TGACA. The ladder of promiscuous affinities for Vismay will contain degenerate versions of the repeat TGACA (in addition to the A/T rich pattern that is likely to be general for all homeoboxes).

2.2) We included two cooperativity terms in the model because it seemed to reproduce our experimental data better. We have, however, simplified this cooperativity term in the new version of the model (see below).

2.3) In the model, non-specific binding arises from electrostatic interactions, and thus, it does not depend on the sequence (whether A,T,G or C). Then, the various types of specific interactions included in the model are consensus interactions and the semi-specific (or degenerate) A/T interactions that likely reflect structural preferences of the homeobox binding to DNA (see

previous point). Therefore, G/C interactions are not explicitly considered; they are however included implicitly in the non-specific binding term that is driven by electrostatics.

2.4) The original statistical mechanical model contained 8 parameters as reviewer 1 correctly states. The spirit of our previous analysis was to build a model that was as detailed as possible in terms of pairwise interactions because, originally, we were expecting binding to be very specific to the consensus sequence. However, in response to the comments of various reviewers, we have carefully revisited the model with the goal of simplifying and generalizing it as much as possible while maintaining is essential features. We have also used all of our experimental data (rather than just the data subset corresponding to the variations of the target site sequence and ionic strength dependence) to test and refine this new version. And, we have also tried other possible models to describe the specific consensus interactions, including one that uses the existing position weight matrix data for engrailed to encode for the consensus interactions (as suggested by reviewers 2 and 3). As result of these efforts, we have implemented a refined model that it is much more straightforward and general, has fewer parameters, and still works much better than a model based on existing PWM data. **The final model used in this revised work only has 4 parameters**: a) electrostatic interaction, b) interaction for each consensus base in the core tetrad, c) interactions with any A or T in positions 1 and 6, or non-consensus A and T in positions 2-5; 4) one cooperative interaction for the full consensus (or half the interaction for 5 consecutive consensus bases: 1-5 or 2-6). We also provide the rationalization for each interaction in the new description of the model (which has been moved to the main text from the supplementary information). This reduced model works almost as well as the original one in reproducing all our experimental data, and does predict very high affinity for the available ChIP-Seq data on Engrailed (see new figures 6 and 7), indicating that it is also capable of recapitulating in vivo binding data. This condensed model is robust, predictive and generalizable (the parameters can be recalibrated with binding data for any other TF).

3. The authors used single molecules measurements (FSC technique) to measure free energy for binding. There are more conventional ways to measure DG for binding that should be used to assess the new methodology.

We do use fluorescence correlation spectroscopy to characterize the binding of EnHD to DNA molecules of different sequence and/or length. The reason for using FCS is that it is a very powerful method to measure binding over a broad range of affinities (sub-nM to almost mM) and provides simultaneous sensitivity to non-specific and specific binding as well as to their interplay. Even more importantly, FCS does give directly the populations of the bound and free forms of the protein, whereas other more conventional methods rely on the analysis of binding isotherms with a specific binding model. This difference is critical when the binding process is multivariate, as we find in EnHD. FCS affords direct determination of the free and bound populations because it detects single molecules in very short observation times (< 1 millisecond), which eliminates contributions from the kinetics of binding/release; which are particularly insidious when binding experiments have net contributions from events with variate affinities (e.g. a mix of non-specific and consensus binding). In other words, using FCS is what allows us to unequivocally assign the binding behavior of EnHD to a variety of DNAs with affinities

ranging from nM to nearly 1 mM and with varying contributions of specific and non-specific binding.

We should also say that FCS is an extremely well established technique, and its strengths to characterize complex binding behavior of biomolecules are widely documented. The use of FCS to characterize binding of biomolecules is so well established that the method is featured in several textbooks, e.g. Single-Molecule Techniques. A Laboratory Manual by Selvin and Ha (CSHL Press). Moreover, even though it may seem non-conventional for the study of protein-DNA interactions, mostly because several simple techniques were readily available early on and to a broad spectrum of researchers, FCS has been used multiple times to study protein DNA interactions *in vitro* and *in vivo.* Some examples are:

https://www.ncbi.nlm.nih.gov/pubmed/10521283
https://www.ncbi.nlm.nih.gov/pubmed/12939158
https://www.ncbi.nlm.nih.gov/pubmed/29872735
https://www.ncbi.nlm.nih.gov/pubmed/28686586
http://jcb.rupress.org/content/217/4/1181

4. The authors argue that the "antenna" works by increasing the frustration for protein-DNA binding and find this reminiscent to protein folding. However, in protein folding, the frustration should be minimal. In the current study, one gets the impression that ruggedness (frustration) is advantageous however, it may have a strong effect of slowing down kinetics. Improving the affinity of protein-DNA is just one aspect but kinetics (and 1D diffusion) might be slowed down dramatically by the frustration.

After reading this comment by reviewer 1 and re-reading our manuscript, we realized that our original discussion on this issue was somewhat confusing. What we find in the binding free energy landscape of genes under Engrailed control is that there is an accumulation of mid affinity binding sites (non-consensus, but still sequence dependent) in the non-coding regions of the gene. Such accumulation of mid affinity binding sites produces frustration or ruggedness. However, these sites accumulate preferably around particular target (consensus, high affinity) sites, which results in a rugged funneled landscape (by accumulating near the target, their basal occupancies increase the average proximity of the TF to the target site). The analogy to protein folding is that folding landscapes are funneled, but are also expected to contain significant leftovers of frustration since natural selection does not remove it all.

Going back to the DNA binding properties of EnHD, it is the spatial distribution of these tracts of mid affinity sites what makes for an effective overall funnel for binding. Of course, the presence of many mid-affinity sites (ruggedness) implies that 1D sliding will be slower than in regions with only non-specific binding. However, the TF search is not performed by sliding alone (here is where the analogy to protein folding ends), it is done by a combination of 3D and 1D diffusion events. Having an accumulation of mid affinity binding sites near the target ensures that the overall search is fast because the dissociation rates from these degenerate consensus sites are relatively high, and their physical proximity to the recognition site increases the probability of the TF encountering the target (by combined 3D and 1D diffusion, see the antenna diagram in figure 6). This behavior is actually what our simple kinetic model for a transcription antenna

nicely recapitulates. It is also in agreement with theoretical models of the search dynamics of a TF in the presence of "traps" (or semi-specific binding sites) in the vicinity of the target site (see for example: Lange et al. Dynamics of the protein search for targets on DNA in the presence of traps. *J. Phys. Chem. B* **119**, 12410-12416, 2015).

In the revised version of the manuscript we have rephrased our description of the binding free energy landscapes of the β3 tubulin gene and EnHD to state these issues more clearly. We have also changed figure 4 to add the TF occupancy along the DNA molecule in addition to the free energy landscape so that these issues are more readily apparent. The revised manuscript now says:

"Our theoretical analysis points to DCB as modulator of the overall affinity and specificity of EngHD to the β3-tubulin first intron. The DNA sequence flanking the target site is indeed rich in A/T clusters [38] that give rise to many potential DCB sites (see m2 in fig. 4a). Calculation of the binding profile for this DNA region with our model shows a rugged free energy landscape with many minima. However, the local minima concentrate around the target site, producing an overall funnel for EngHD binding (m1 in fig. 4c). Such binding properties are reminiscent of the energy landscapes associated to protein folding[45], binding and function[46]. An important implication is that EngHD can occupy multiple mid-affinity binding sites surrounding the target site in a strategy that enhances the overall affinity without involving very strong interactions, that is, while maintaining relatively fast dissociation rates. This behavior is consistent with predictions from theoretical modeling[32]. A rugged funneled binding landscape also introduces resilience to mutations on the target site, which is exactly what we observe in the experiments of fig. 2a. The mechanism behind binding resilience becomes apparent by comparing the binding profiles of the natural sequence (with TAATTG in the target site) and the sequence with a consensus, or SB, site. The latter produces a landscape with a global minimum and highly localized binding (m1 in fig. 4c), whereas the natural sequence lacks a sharp free energy minimum but maintains all flanking DCB sites (m2 in fig.4c), which in absence of competing SB see their occupancy raised, thereby buffering the overall drop in affinity."

We have also incorporated the transcription antenna kinetic model into a new section of the main text so that the kinetic connotations of the mechanism, and its connections with prior theoretical work are more clear and discussed in more depth.

5. Figures 6 and 7: how come the protein has lower free energy to non-specific binding sites than to the consensus site. This is counterintuitive. Is there a meaning then to a specific binding site? The meaning is that the energy landscape for target site is anti-funnel.

We suspect that reviewer 1 may have gotten confused by the prokaryotic model for gene expression. In eukaryotes there is not a single recognition site per gene (unlike in a prokaryotic operon), but several target sites spread around the non-coding regions of the gene of interest, which are then classified globally as regulatory regions without being placed at a specific location relative to the transcription initiation site (they operate from a long distance in *cis* and even in *trans*). What our analysis finds is entirely consistent with this general description for eukaryotic transcription, namely that, in addition to the consensus target sites (the deepest spikes in the free energy landscapes: shown in teal color in figs. 6 and 7), the flanking regions also have

myriads of mid affinity sites (degenerate consensus sites), that increase the overall occupancy of the TF and which are found much less frequently in the coding regions (even though these are still A/T rich). I.e. this pattern is very apparent upon comparison of binding profiles for the purple (regulatory) and orange (coding) regions shown in the figures. What the rugged funnel for binding does in this case is to increase the probability that the TF is near (but not on) the target site.

6. Figure 7: The minimum to the left of the consensus site show a depletion of A and T content. Also does one expect a flanking region to be so long (hundreds of bp)?

In *Drosophila*, the genome has 57% A/T, which means that the random distribution of its genome is 28.5% for each of them, versus 21.5% for G or C. These are the levels at which figure 7a converges as the distance from the TSS increases (both downstream (left) and upstream (right)). What the figure shows is that the AT content is well above the global 57% in the region just before the transcription starting site (TSS). The TSS is not where the consensus target site for the TF is located, but is where the RNA polymerase starts to transcribe. The region upstream the TSS is where the promoters tend to accumulate (as labeled in the figure). This region is also rich in TF target sites, and it can be very long (on average about 500 bp as figure 7a shows, but for certain genes the regulatory sites where TFs bind can be thousands of bps away).

7. It is argued that the DNA diffuse with coefficients that decrease proportionally to their size, indicating that they behave as rigid dsDNA. How a 600 bp DNA can be rigid? Why this excludes possibility of more compact DNA conformation?

We realize that this comment was misleading. We have measured the diffusion coefficient for all these DNAs using FCS, and thus we are not assuming that they are rigid rods. The persistence length of B DNA is about 50 nm in conditions similar to what we use for these experiments (or about 150 bp), so our longest DNA segment is 4 persistence-lengths long. What happens is that for these range of lengths (75 to 600 bp), the deviation of the diffusion coefficient from one just estimated assuming a rigid rod is relatively small. There are in fact several measurements of diffusing DNA available, both in vitro and in cells, and our results for the diffusion coefficient are consistent with them. For instance, the diffusion coefficient for B DNA changes according to the simple formula (Lukacs, G. L. *et al. Journal of Biological Chemistry* **275**, 1625-1629, 2000):

$$D(DNA) = 490 \times 10^{-8}\, cm^2 s^{-1} \cdot [bp\ size]^{-0.72}$$

as opposed to the $[\text{bp size}]^{-1}$ dependence expected for a rigid rod. However, in the revised manuscript we have eliminated this comment to avoid misleading the reader, and especially, given that this issue is of no relevance, or significance to our study, observations and analysis. The new sentence states: *"In FCS experiments these DNA molecules diffuse with coefficients that decrease proportionally to their size (fig. 5a) in a manner consistent with previous studies of the length dependence of B DNA's translational diffusion coefficient[49]."*
In which reference 49 is the one given above.

Reviewer 2

In their manuscript, Castellanos and Munoz investigate the DNA binding properties and specificity of the Drosophila TF Engrailed (En). First, they employ FCS to measure the binding affinity of En to several DNA sequences (75bp) from an intron sequence from the b-tubulin 3 gene. They find that En binds not only to its target site, but also to degenerate sites embedded within this DNA sequence, and that these interactions depend on the salt concentration. Based on these results, as well as the Xtal structure of the En DBD with DNA, they parametrize a statistical mechanics model for En DNA binding. Using this model, they then investigate the DNA sequences around En binding sites, which reveals large numbers of potential degenerate binding sites. Thus, the authors propose that En can interact with DNA either nonspecifically, can bind to the consensus site, or bind to the degenerate sites. They then posit that the number of degenerate sites may act as antennae that direct En binding to its target sites in vivo. By sequence analysis, the authors show that degenerate sites are enriched at non-coding regions at several genes, and less enriched over coding regions. Using a kinetic model, they indicate how these degenerate sites may direct TFs to their target site, i.e. act as antennae. This manuscript conveys a potentially interesting hypothesis, in that the DNA sequences surrounding promoters and enhancers are enriched with degenerate sequences for the relevant TFs that regulate said genes (named "antennae" by the authors).

We appreciate reviewer 2's view that the hypothesis of transcription antennas in eukaryotic systems is interesting and worthwhile testing. This was exactly our motivation for pursuing the biophysical experiments and theoretical analysis described in this manuscript, and is also a view shared by the other 3 reviewers of our manuscript.

While this is an interesting hypothesis and worthwhile testing, I am however not convinced that the experimental data provided in this manuscript supports this hypothesis at this point. I thus think that this study is too premature to be published in Nature Communication.

We find this point of view unwarranted. The observation that "*the DNA sequences surrounding promoters and enhancers are enriched with degenerate sequences for the relevant TFs that regulate said genes…"* is unquestionable, and well documented in the literature (refs. 15, 25, 26 in manuscript). Straightforward bioinformatic analysis of genome sequences suffices to show the accumulation of degenerate consensus binding repeats for relevant transcription factors in the regulatory regions of eukaryotic genes. This type of sequence patterning is precisely what is used to define the regulatory regions of eukaryotic genes, which are otherwise difficult to identify given their lengths (thousands of bp), varying location from the transcription initiation site, and lack of distinctive sequence signals such as the ones of prokaryotic operons.

However, **the key to the transcription antenna hypothesis is to demonstrate that these tracts of degenerate consensus repeats found near genes have strong cumulative effects on the binding behavior and DNA occupancy of the relevant transcription factors** (those that control the expression of the gene). This is precisely what our experimental data show unambiguously, namely that the DNA binding behavior of EnHD is not just due to the expected combination of consensus (specific) and non-specific (sequence independent) binding modes, but

consists of a ladder of affinities that grow roughly proportionally to the degree of similarity between the site and the consensus sequence. In retrospect, this may seem an obvious result, but nobody before us has experimentally determined the contribution that degenerate consensus repeats have on the overall binding of a eukaryotic transcription factor to the flanking (regulatory) regions of a gene under its control. Our experimental results using high sensitivity biophysical methods and theoretical analysis of the data leave no room to ambiguity. More importantly, we discover that, despite the mid-affinities involved in EnHD binding events to degenerate consensus repeats, their contribution to overall occupancy of the gene regulatory region is actually enormous because of their sheer numbers. The contribution is such that the expectation is for the TF to be bound to the myriads of degenerate consensus repeats around the target site for most of the time. Therefore, binding to these tracts of degenerate consensus repeats explains two major molecular puzzles of eukaryotic transcription: how to control the occupancy of the functional site and how to ensure that the TF can find its target quickly and efficiently when the gene needs to be turned on or off. Moreover, the implementation of degenerate consensus binding energetics into a simple statistical mechanical model allows for specific ***quantitative*** predictions of Engrailed binding affinity to any DNA sequence. The model is a powerful tool compared to standard bioinformatic analyses because it permits to address quantitative questions about affinity and occupancy using just genomic sequence data, as we do in the last section of the manuscript.

Comments follow below:
1. The authors make a case that the Antenna model is general. They however only provide data for En. I am thus not convinced that this is a general mechanism. First, as the authors state on line 253 that Drosophila genome has a high AT content in intergenic regions, whereas this drops in coding regions. Purely random base arrangement might thus generate the proposed accumulation of En sites outside all genes (but not specifically for En). Conversely, the Antenna model would not hold for TFs with a high GC content in their recognition sequence. To make a strong statement about a general mechanism, an "Antenna" sequence arrangement has to be shown for multiple, diverse TFs.

The elements for a transcription antenna are: **1) promiscuous semi-specific binding of the TF and 2) tracts of degenerate consensus repeats present on the regulatory regions of target genes**. We demonstrate these two elements for the transcription factor Engrailed, as reviewer 2 acknowledges. Reviewer 2 also argues that this is just one observation, which in her/his view does not establish generality.

Although we recognize that our work focuses on only one TF, we must say that our analysis of binding energetics together with existing structural knowledge of protein-DNA complexes and conventional bioinformatic genome analyses permits us to safely extrapolate because all the molecular elements required for the transcription antenna mechanism are generally present in eukaryotic transcription factors and genes. The vast majority of eukaryotic transcription factors recognize very short DNA sequences (6-8 bp). Whether the DBD are homeodomains, helix-loop-helix or zinc fingers, specific binding takes place by inserting an alpha-helix into the major groove. Therefore, the pattern of protein-DNA base interactions is essentially the same for all these TF classes. What we find from our experimental and theoretical analysis, as well as from

the structures of protein-DNA complexes, is that base-aminoacid interactions involved in base recognition for specific binding are pairwise, very limited in numbers, and mostly additive (with the exception of a general, entropy-based cooperative interaction). This type of sparse, additive, pairwise energetics lead by definition to promiscuous binding. In fact, the specific binding of eukaryotic TFs has always been a structural/molecular puzzle given the type of protein-DNA interactions found in the 3D structures of the complexes (this is very apparent in fig. 3a of the manuscript for EnHD, but such general binding pattern is found in all other TFs). Therefore, the real surprise would be that other TFs do not bind promiscuously as EnHD does.

Likewise, our analysis with the statistical mechanical model allow us to make quantitative predictions of binding affinity to EnHD for any sequence, which confirm the generality of the transcription antenna mechanism for Engrailed. A similar quantitative analysis is not yet possible for other TFs (parameterizing the model for other TFs requires dissecting its binding energetics using high-resolution biophysical experiments rather than high throughput binding assays). Nevertheless, once we have demonstrated that TFs bind promiscuously to degenerate consensus repeats, it is straightforward to investigate the DNA profiles of transcription antennas using conventional bioinformatic analysis (i.e. DNA sequence patterning). As we mentioned above, there are in fact several papers in which other authors have documented tracts of TF degenerate consensus repeats in the regulatory regions of eukaryotic genes. The key aspect of the transcription antenna mechanism requires determining the contribution of promiscuous binding to the overall affinity of a TF for DNA. We have done this for the first time on EnHD. Once our identification and quantification of degenerate consensus binding for EnHD becomes available to the scientific community, other works can follow by looking for the same behavior on other TFs and thus demonstrate generality. It seems an impossible standard to demand that both the discovery of a new mechanism and the demonstration of its generality be done simultaneously in a single publication. A first observation is always interesting and important. The interest it elicits in the scientific community is what subsequently leads to further studies from the original and other authors to assess its generality.

We would also like to clarify two important technical points that seem to have confused reviewer 2.

Point 1: Drosophila's genome has a 57% AT content, which obviously implies that there is a higher probability of finding AT than GC repeats by random happenstance. However, the accumulation of degenerate consensus repeats in regulatory regions and depletion in the coding regions that we identify have been calculated in reference to the overall 57% AT probability of the *Drosophila* genome. All the numbers presented in the tables (in fig. 7a and in supplementary materials) are relative to the probability of finding such pattern at random in sequences with 57% AT (frequencies calculated with base probabilities: A=0.285, T=0.285, C=0.215 and G=0.215, see supplementary materials for the formulas). Fig. 7a also shows very distinctly that the region around the transcription starting site has a spike in A/T content that is well above the 0.285 average for A and T, and which quickly decays down to the 0.285 level in the regions downstream and further upstream the TSS.

Point 2: The antenna mechanism involves promiscuous binding to tracts of degenerate consensus repeats. For EnHD the consensus sequence is TAATTA, and thus, the degenerate repeats are necessarily composed of As and Ts. However, their affinities are not all the same but are

proportional to how similar to the consensus the degenerate repeat is. This means that the base arrangement is also important (e.g. XAATTX leads to much stronger binding than XTATAX). However, this does not mean that all transcription antennas for all TF must be composed of tracts of AT repeats. **For each transcription factor the degenerate consensus repeats will be defined by its own consensus binding sequence**, otherwise the mechanism would not have any signal specificity. For many homeodomains their degenerate repeat patterns will be similarly AT-rich since a large fraction of homeodomains recognize consensus sequences composed of only As and Ts with different arrangements; but the order of bases will make them somewhat selective (partial overlap may also have functional advantages for TFs that work concertedly or anti-concertedly, a common trait in eukaryotes). The transcription antennas for other TFs will respond to the specifics of their binding energetics. For example, the TF Max recognizes the sequence CACGTG, and thus, the degenerate consensus repeats for Max should be composed of versions of that pattern, like for example the core tetrad XACGTX, or even the motif CXCGXG, which does not contain a single A or T. Presumably, the same will be true for TFs with HLH or zinc-finger DBDs.

2. As there exists ample data on En binding sites within the Drosophila genome, e.g. by ChIP or DNAseI footprinting etc. The authors write in the discussion that such data provides "too many hits and little correlation occupancy and gene expression levels". This is a very strong statement and should be supported by more evidence. In contrary, the authors should show that their statistical model recapitulates obtained in vivo binding profiles.

Reviewer 2 raises an important point. We should point out that our comment in the manuscript that the correlation of *in vivo* site occupancy of eukaryotic TFs measured by ChIP-Seq or other methods does not strongly correlate with the patterns of gene expression was not based on our own results, but on several studies by other authors, who after analyzing both types of datasets jointly found poor correlations (ref. 17, 19-22 in revised manuscript). Nevertheless, we have modified this sentence of the introduction to make this point more accurate: "*As result, the in vivo site occupancy patterns of eukaryotic TFs are more complex than predicted by their in vitro site-specific binding profiles*[19, 20] *and do not strongly correlate with the actual levels of gene expression*[17, 21, 22]"

This change notwithstanding, we do agree with reviewer 2 in that our statistical mechanical model of EnHD binding gives a unique opportunity to calculate the binding affinities of Engrailed to all the hits found in these experiments and determine whether *in vivo* site occupancies recapitulate the *in vitro* affinities of those sequences. In our revised manuscript we now include the results of comparing the ChIP-Seq data available on Engrailed (ENCODE Project, experiment ENCSR952TDU) with the calculations with our model. What we find is that all of the 2,226 ChIP-seq fragments correspond to high affinity for EnHD according to our analysis (95% of the fragments have predicted affinities of 10 nM or higher). 20% of them are very high affinity (near 1 nM), and another 70% correspond to affinities of approximately 5 nM. This result confirms that the DNA fragments picked up by ChIP-Seq (from 100 to 500 bp long) do correspond to very high affinity sequences for EnHD. However, we also found out that there are no ChIP-Seq fragments corresponding to the β3-tubulin gene, which is consistent with the idea that *in vivo* TF occupancies are not necessarily indicative of the genes under control of the TF. Following reviewer 2's request, we now include a panel in figure 6 (revised fig. 6a) that

shows the histogram of affinities predicted by our model for the ChIP-Seq data on Engrailed (ENCODE project).

In the revised manuscript we also include the following paragraphs:
In section **The binding profile of a transcription antenna,** *"For transcription antennas to be functionally significant, promiscuous DCB should accumulate on the regulatory (non-coding) regions of the gene whereas the opposite should be true for the coding regions (exons). We can investigate this question using the statistical mechanical model to calculate EngHD binding profiles for full gene sequences. Before embarking on large-scale DNA sequence profiling, however, we performed a test of the biological significance of the affinity predictions produced by our statistical model. For this test, we calculated the predicted affinity of the 2,226 DNA fragments (each about 100-500 bp long) that have been identified in ChIP-Seq experiments on transgenic flies as DNA regions that bind engrailed in vivo (ENCODE Project, experiment ENCSR952TDU). The statistical mechanical calculations predict very high binding affinity for all ChIP-Seq fragments, with over 90% of the fragments presenting predicted K_D values between 1 and 10 nM (fig. 6a). These affinities are comparable to what we have measured in vitro for the 150 bp segment from the first intron of β3-tubulin carrying the consensus site (fig. 2a). We can thus conclude that the binding profiles of the statistical mechanical model have biological relevance."*

and in the **Conclusions,** *"Binding events concentrated on the antennas rather than on target sites provides a simple explanation of why crosslinking data on eukaryotes produces many more hits than expected from the number of genes under control of a given TF, and relatively weak correlations between site occupancy and gene expression levels[20, 21, 57]. In this regard, we note that whereas the available ChIP-Seq data on Engrailed do reflect high affinity binding (fig. 6a), it does not contain any hit corresponding to the β3 tubulin gene."*

3. The statistical model contains > 8 parameters, and is parameterized using only a 6 binding experiments. It is not clear to me how accurate / robust this model is. Moreover, the description of the parameters in Fig. 3b is unclear (circles, boxes etc.). It would be more informative to provide the actual parameters in a table, including parameter confidence intervals, and a parameter sensitivity analysis. Then the model could also be validated with the native ChIP profiles.

The original statistical mechanical model had exactly 8 parameters and was parameterized using the binding data from the 6 DNA molecules of figure 2, plus the ionic strength dependence of the binding to those molecules (which led to determination of the electrostatic interaction term), and the data from molecules m4 and m5 from figure 5, which provided information about the generic non-consensus AT interaction. The original model was developed with the intent of recapitulating the interactions observed in the 3D structure of the EnHD complex with DNA plus a cooperative term necessary to account for the non-additivity of the entropic loss concomitant to binding (most of the entropy is already lost when the first interactions are made, which results in an effective strengthening of the last ones; i.e. when all the consensus interactions are made). Our modeling approach was to use specific experiments to independently set the values of individual parameters, and then test the predictive power of the model with independent experiments.

From the comments of both reviewers 2 and 3 (see below), we realized that our approach may not have been the most consensual one. It had the additional inconvenient of making it difficult to rigorously evaluate the statistical significance of the parameters and their values beyond the model's ability to predict experimental results that it had not seen before (in our view the predictive power of a model is a most powerful test of significance, but it does not lend itself to statistical analysis). Accordingly, in response to both reviewers' requests, we decided to use a more conventional statistical approach of fitting the model parameters to all the experimental data globally, and estimate the statistical significance of the parameters using covariance analysis (from the Jacobian matrix of the best fit). In addition we performed principal component analysis to find an optimally reduced set of parameters. From this exercise, **we have been able to simplify the statistical mechanical model to only 4 parameters** without significantly sacrificing the model's performance (the sum of least squares for all of the data). We also tried a model implemented with base-specific interactions derived from the available 6x4 position weight matrix (PWM) for Engrailed measured in B1H experiments (we did this per reviewer 3's suggestion) plus the same non-specific electrostatic term that we have used for all of the structure-based models (this model has 6x4 fixed parameters from the PWM plus the electrostatic term as single fitting parameter). The reduced structure-based model with 4 parameters performs much better (about 50-fold smaller sum of least squares) than the PWM-based model. To determine the statistical significance of both models we performed the Fisher test, which indicated that the probability that the performance of the statistically simpler model (PWM with just 1 free parameter) is comparable to that of the complex one (structure-based with 4) is less than 10^{-9}.
In the revised manuscript we have accordingly opted for the reduced structure based model (rather than the original one), which we now describe in depth in the main text rather than in supplementary information (this was also requested by reviewers 3 and 4). The original manuscript included a table with the parameter values that we used as part of figure 2 (using symbols to connect with the schematic description of the model given in this figure). In the revised manuscript we still provide the table; but in addition, we describe the 4 relevant interactions: 1-consensus based in the core tetrad; 2- A or T in positions 1 and 6 and non-consensus A or T in the core tetrad; 3-a cooperative term for the full consensus sequence (or half its free energy when the consensus site lacks the first or last base); 4-electrostatic interaction term). And, we justify their physical relevance and provide the statistical significance of their values (determined from the global fitting errors). Furthermore, we also discuss the PWM-based model and its comparison with the structure based model (both in terms of performance and statistical significance). A comparison of the performance of both models (4-parameter structure-based and PWM-Electrostatic) is given as new supplementary figure s1. Finally, as explained in our response to issue 2, we have directly tested the structure-based statistical mechanical model with the ENCODE ChIP-Seq data on Engrailed (see above). These results are given in the new figure 6a.

We believe that all these additions and tests fully address all the requests made by reviewer 2 regarding the statistical mechanical model and its significance.

4. The authors completely ignore the contribution of chromatin. The Drosophila genome is packed into chromatin which modulates motif accessibility genome wide. Nucleosome depleted

regions around promoters however enable local TF binding. Chromatin thus may be a key driving factor for TF site selection. The authors should discuss this.

We are well aware that the control of gene expression in eukaryotes is multilayered and complex, and that chromatin structure and dynamics play a key role in it, as we indicated in the introduction of our original manuscript, citing two key articles: a review by one of the seminal authors working on chromatin (G. Felsenfeld) and the paper by Cairns in *Nature* (2009), refs. 8 and 9 in the original manuscript, and 9 and 10 in the revised one. Our view is that chromatin structure and dynamics exert the master control of gene expression since, to be transcriptionally active, the gene needs to be devoid of nucleosomes. The transcription antenna mechanism that we propose in this work acts at a lower level, once that a particular region becomes transcriptionally active (euchromatin), by channeling the relevant transcription factors to the region of interest via myriads of mid-affinity binding events around the regulatory region of the naked gene. In other words, the transcription antenna operates once the DNA region is naked to permit binding of TF molecules. In our original manuscript, we decided not to discuss the role of chromatin in depth to simplify the discussion (already loaded with new concepts) and given that the role of transcription antennas, like any mechanism based on thermodynamic binding equilibria, only operates by definition on DNA that is already naked since TF binding does not carry sufficient binding free energy to displace the nucleosomes.

However, as per reviewer 2's request, in the revised manuscript we now discuss the transcription antenna mechanism in the context of transcriptionally active DNA and under the control exerted by chromatin, and we include some additional references to highlight the importance of chromatin dynamics in transcription control. Particularly, the role of chromatin in reference to transcription antennas is discussed in the following additional excerpts: in the section describing the transcription antenna kinetic model (which is now part of the main text rather than being in sup. mat.): *"The model aims to capture the fundamentals of the binding/release kinetics of a TF to a gene that contains long tracts of DCB sites in its non-coding (regulatory) regions. Binding of the TF requires that the region of interest is transcriptionally active and thus depleted of nucleosomes via chromatin dynamics*[51, 52]*."*
References 51 and 52 are the works of Hihara et al. *Cell Reports* (2012) and Nagashima et al. *J. Cell Biol.* (2019). And in the Conclusions: *"Once the DNA region becomes accessible by chromatin dynamics*[51]*, and thus transcriptionally active, the antenna attracts TF molecules that remain loosely associated to the gene of interest through a highly dynamic exchange among the myriads of mid-affinity binding sites (< SB but >> NSB) that are present in the antenna."*

5. "the binding free energy landscape around the cognate site is rugged, resulting in binding competition" I am not sure what this sentence means, this should be better explained.

We have completely rewritten this section of the manuscript to make all its statements as clearly as possible. The new section reads: *"Calculation of the binding profile for this DNA region with our model shows a rugged binding free energy landscape with many minima. However, the local minima concentrate around the target site, producing an overall funnel for EngHD binding (m1 in fig. 4c). Such binding properties are reminiscent of the energy landscapes associated to protein folding*[45]*, binding and function*[46]*. An important implication is that EngHD can occupy*

multiple mid-affinity binding sites surrounding the target site in a strategy that enhances the overall affinity without involving very strong interactions, that is, while maintaining relatively fast dissociation rates. This behavior is consistent with predictions from theoretical modeling[32]*. A rugged funneled binding landscape also introduces resilience to mutations on the specific site, which is exactly what we observe in the experiments of fig. 2a. The mechanism behind binding resilience becomes apparent by comparing the site occupancy of the natural sequence (with TAATTG in the target site) and the sequence with a consensus, or SB, site. The latter produces a landscape with a global minimum that concentrates most of the occupancy (m1 in fig. 4c), whereas the natural sequence lacks the sharp free energy minimum but maintains all flanking DCB sites (m2 in fig.4c), which in absence of the competing SB site see their occupancy raised, thereby buffering the overall drop in affinity."*

We have also added the occupancy profiles to the binding free energy landscapes of figure 4c to facilitate the reader the conversion between free energies and populations.

Reviewer 3

This paper on the biophysics of TF-DNA interaction explores the hypothesis that several weak binding sites for a transcription factor (TF) in the vicinity of a strong site may serve the special purpose of attracting and trapping the TF molecules to that locus, thereby increasing the effective local concentration of the TF. The authors draw an analogy of this phenomenon to an antenna. The study is performed mainly in the context of the TF Engrailed (EN) binding to the regulatory region of a particular gene (β3-tubulin) in Drosophila. The main contributions of the work are (a) use of fluorescence correlation spectroscopy (FCS) to measure dissociation constant (Kd) values for several binding sites for EN, (b) using these Kd data to fit a statistical mechanical model of TF binding to a longer DNA sequence, and (c) use of such a model on the regulatory region of the β3-tubulin gene to claim that the region's total affinity for the TF is completely dominated by contributions from weak sites, which play the important role of increasing the local TF concentration for the strong site in the region.
Overall, the paper presents a very interesting technical study of a phenomenon that others have written about as well, including at the level of biophysical models. The authors study an important topic, and employ interesting novel approaches to the problem. The main claim made needs to be made more often, or at least examined more often, than it is today.

We thank reviewer 3 for her/his positive assessment of our work, including agreement with the importance of the topic of how eukaryotic transcription factors track and control their target genes, alignment with our hypothesis of transcription antennas, as well as her/his positive outlook on our biophysical approach, which, as reviewer 3 acknowledges, is relatively novel in this research field and therefore always more difficult to be understood/accepted.

At the same time, I have a few main concerns and several technical questions that I write below.

We have very carefully looked at all the comments/concerns/suggestions made by reviewer 3, which we found very useful to make our manuscript more clear and stronger. We provide below a point by point description of how we have addressed them.

General Comments (they go from 1 to 4)
1) I do not think the observation of the 'antenna' has to be made by first introducing binding data from a less-used approach and a new computational model. Using a simple position weight matrix model of Engrailed binding (which is available), one could probably have reached similar conclusions, at least qualitatively, perhaps even quantitatively.

We understand that reviewer 3 may feel more comfortable with the widely used high-throughput binding assays, but the special characteristics of the FCS method are precisely what allowed us to determine the contributions from degenerate consensus binding, which are difficult to extricate from other contributions in conventional binding experiments. For instance, traditional *in vitro* binding assays (e.g. gel shift assays) use short DNA sequences, which do not allow to determine the contributions from the accumulation of large numbers of mid affinity sites, and need slow dissociation rates relative to the electrophoretic mobility of the complex (or they will produce smear and will be hardly quantitative), and are analyzed as simple binding isotherms (binding to a single site with fixed affinity). In parallel, high throughput *in vitro* assays are specifically designed to detect the highest affinity binding (as a way to minimize false positives), which then leads to the very specific consensus binding profiles or binding logos that we have grown accustomed to.

In contrast, FCS has a very broad range of sensitivities from sub-nM to almost mM, it works best with larger DNAs (so the difference in translation diffusion coefficient between bound and free is most apparent), has very fast time resolution (better than 1 millisecond) that permits quantitative analysis of even the weaker binding events, and detects the composite binding to all the binding sites present in the DNA without introducing thermodynamic biases (such as the biases introduced by the electric field and interactions with the matrix in gel shift assays).

The use of FCS is what really allowed us to discover the importance of degenerate consensus binding and characterize its overall contribution to TF DNA recognition. This is why we think it is essential to introduce the problem starting with the FCS experiments that we carried out to determine the effects on affinity of changes in the sequence of the target site (consensus and variants diverging from the consensus). These are the most conventional studies (make mutations on the DNA target site and measure the changes in affinity), and they highlight the binding resilience to mutations on the target site, a property that is strongly indicative of a strong contribution from promiscuous binding to other sites on the DNA.

However, we do see the point of reviewer 3 of the importance of interpreting that first set of FCS binding experiments in a more conventional way (using available PWM data) to most effectively convey the message to the wide community of researchers interested in gene expression and TF binding to DNA. Therefore, in our revised manuscript we introduce the problem using the FCS binding experiments of affinity versus sequence changes in the target site (figure 2), and follow

with an analysis based on the PWM for Engrailed. The PWM provides a probability for each base to be found in any of the six positions of the target site (a 6x4 matrix). The PWM can then be used to determine the expected changes in affinity (relative to the consensus) from the probabilities of the particular base combination found in the sequence. When we estimate the changes in affinity for the six DNAs of figure 2a using the PWM we do see a correlation. However, the correlation is relatively weak and, most importantly, the scale of the predicted changes is off by many orders of magnitude (i.e. the PWM predicts drastic changes in affinity whereas we see relatively weak effects). These observations demonstrate that the overall binding process is not just controlled by specific consensus binding to the target site but it contains other important factors (which we then dissect using the statistical mechanical model). We have reworded the full section that discussed the experiments from figure 2a to use the PWM as reference standard. The revised text is as follows (in section **Specific versus non-specific contributions to EngHD binding to DNA)** :

"These experiments reveal evident changes in affinity. However, the decreases in affinity are seemingly small relative to how much the binding site diverges from the consensus, most notably for CGTGTT in which only one consensus base is retained. To further investigate this issue, we compared our affinity results with the position weight matrix (PWM) for Engrailed obtained from bacterial one-hybrid assays[40]. The PWM recapitulates the consensus binding site (fig. 2b), and predicts a decreasing binding probability as the target site sequence diverges. However, the PWM predicts changes in binding probability that are much sharper (many orders of magnitude) than what we find in our experiments (fig. 2c). The PWM also predicts huge differences in binding (several orders of magnitude) for target sites that exhibit very similar affinity in the context of the 75 bp β3-tubulin intron DNA fragment and our experiments. The implication is that EngHD DNA binding is not just dictated by pairwise interactions with the consensus motif but is more complex. The most likely explanation is that EngHD binds promiscuously to the flanking DNA sequence to an extent that partially compensates the degradation and even elimination of the consensus site."

The revised manuscript also includes two additional panels in figure 2, one with the PWM logo, and a second one with a plot of the measured affinities versus the product of the six PWM probabilities (new figures 2b and 2c) for each variant to illustrate that binding is more complex than simple consensus specific binding to the target site. We think this comparison makes that key point much more clearly, especially to a broad readership, and thank reviewer 3 for her/his suggestion.

2) The main claim -- of weak binding sites enhancing local TF concentrations – has been made previously, e.g., in the relatively recent work of Boris Adryan and Sridhar Hannenhalli. I believe the work presented by the authors here is more quantitatively detailed, to their credit, but the claim is not novel.

We must say here that we were not aware of the prior work by Adryan and Hannenhalli on the bioinformatic analysis of DNAse footprinting data on gene regulatory regions, and most specifically on regions containing proximal clusters of enhancer sequences which they term archipelagos, and for which they find correlations with high *in vivo* occupancies determined from DNAse footprinting data. This is indeed very relevant to our work and we thank reviewer 3 for pointing us to their papers. We now cite their work in the introduction (including a reference to

their BioRxiv article, which is the most relevant one to this issue, and since we could not find a follow up peer-reviewed publication on this topic): "Analysis of existing DNAse footprint data reveals that clusters of spatially proximal enhancers (or archipelagos[29]) around a functional specific binding site correlate with increased TF occupancy *in vivo*[30]."

However, we should also say that whereas Adryan and Hannenhalli have found a correlation between *in vivo* occupancy and their archipelagos (clusters of enhancer sequences), they have not measured the actual binding effect, nor have they dissected the energetics of the process, or determined the overall contribution to binding of a transcription factor (and how is that related to the promiscuous binding properties of the TF itself). In contrast, we do all those things in this work. In many ways, their prior observations strengthen our work by providing a connection of our observations and transcription antenna mechanism with *in vivo* occupancy data. We emphasize this connection in the revised manuscript by running our statistical mechanical model on all the DNA fragments derived from ChIP-Seq data in *Drosophila f*or Engrailed, where we see that all the ChIP-Seq fragments correspond to very high affinity to engrailed (K_D<10 nM) according to our analysis (see our response to related specific points below)

3) I am not convinced that the extension of the model's predictions, shown to fit observations on sequences of length up to 600 bp, necessarily generalizes to sequences of length ~10 Kbp, and important step in the authors' demonstration of the antenna concept.

Although we cannot think of any obvious physical factor that could change the mechanism of binding of a TF to DNAs when they go from 600 bp to a few thousand kbp (TF binding is extremely local and should be independent of the DNA length provided that it is long enough, devoid of nucleosomes and in B-from), we do think that the point raised by reviewer 3 could resonate with part of the potential readership of this work and wanted to address it. Here we were pressed between a rock and a hard place because testing longer DNAs really push the sensitivity limits of our binding assays (which are still superior for addressing this question than other available alternatives). However, we did synthesize a DNA of 7,219 bp that contains the entire β3-tubulin gene sequence without the 5' and 3' UTRs. This sequence contains the full, very long intron 1 where our target site is, and thus contain a significant fraction of the gene's regulatory region, but by dropping the UTRs we could decrease the predicted overall affinity somewhat, and thus still investigate its binding using FCS. The FCS experiments do not allow to fully determine the K_D for this DNA with sufficient accuracy, but they do show very strong binding (diffusion retardation) of EngHD at very low DNA concentrations of just 100 pM. These experiments indicate that the overall K_D of EngHD to the 7,219 bp β3-tubulin gene DNA is sub nM (our experimental upper estimate for the K_D is ~700 pM). We now show the FCS experimental data for this very long DNA in the revised manuscript as figure 5c. We also plot the value of affinity determined experimentally in the revised figure 5b, together with the affinity predicted by the reduced 4-parameter structure-based model.

4) The narrative approach of hiding nearly all modeling details in the main text (and presenting them instead in Supplement only) does not quite work in this paper because the modeling is a key aspect of the work and without clarity on how the model works, it is hard to interpret its predictions and findings.

We introduced all the technical details about the modeling part of the work in the supplementary materials thinking about potential readers that may be interested in the general results but could not follow the modeling details (to expand the scope of the potential readership), whereas the researchers in the specific field and theoretically savvy outsiders could always get all of the required information from the supplementary materials. However, per reviewer 3's request, in the revised manuscript we now include the description of the statistical mechanical model for binding, and also of the kinetic model of the transcription antenna. This narrative approach lays all the key information in the main article, which we also prefer (we hope that *Nature Communications* will allow the extra space in the manuscript required for implementing this change).

Specific comments:
"decreases in affinity that were proportional to their divergence from the consensus". How do the observed changes in affinity track with predictions from say a PWM model?

As explained in the previous point, we have now introduced a comparison between the changes in affinity upon modifying the target site's sequence that we observe experimentally and the predictions from the PWM for Engrailed. This is discussed in the text and the correlation shown as new figure 2c.

"key implication of these results is that EngHD is promiscuous in its specific binding to DNA." How is this interpretation reached? Please clarify.

This interpretation is reached from the quantitative analysis of the binding data for the various DNA molecules of this study (fig. 2 and fig.4 and 5). As we indicated, the binding affinity drops minimally upon mutating the target site away from the consensus sequence. This suggests that binding occupancy is distributed over many other DNA sites (for which the sequence is not modified), that is, that binding must be promiscuous. In the revised manuscript we have tried to state this issue more clearly given that we now compare the FCS binding results with the PWM predictions (here is important to remind reviewer 3 that the approaches used to derive PWM data select only the highest affinity binding, and so, they are largely insensitive to promiscuous mid-affinity binding). However, the demonstration that binding is promiscuous comes from the analysis of the experimental data with the statistical mechanical model, which produces binding profiles with many mid-affinity sites. These mid affinity sites are not occupied in the presence of the consensus specific site (in which case all occupancy is localized in it), but when the consensus sequence is degraded, these alternative sites start to be occupied thus buffering the drop in affinity. We discuss this issue in more depth in the revised manuscript to avoid misunderstandings, and also include the occupancy profile together with the binding free energy landscapes in figure 4 to make the point more evident. These changes together with the new comparison between experimental affinities and PWM (figure 2c) should make this point clear.

"include specific interactions with each consensus base, plus two cooperative terms that kick in

when the binding site contains the first 5 bases or the entire consensus site. For simplicity, we exploited structural similarity to group consensus interactions in four types: T1 + T5, A2 + A3, T4, and A6." The model needs better explanation. If one cannot understand even the basic ideas without the Supplementary Methods, it is hard to appreciate the work.

We are deeply sympathetic to reviewer 3's frustration with the coming and going from main article to sup. inf. that was imposed on the reader to follow through our original manuscript. Accordingly, we have included the full description of the model in the main text. Having the model(s) with all the equations and explanations of the physical origin/rationalization for each parameters (and specifically for the more subtle cooperative interaction) should facilitate the reader to follow our reasoning, the physical basis of the model and how it is used to interpret the experimental data.

Related specific questions: (they go from 1 to 5, plus a few more unnumbered)
1) How was the value of 510-4 obtained for w_0 ?

The statistical weight w_0 reflects the ratio between the rate constants for forming and breaking the encounter complex between EngHD and DNA in the absence of any interactions. It depends on the diffusion coefficients of the two molecules (and mostly on that of EngHD, because it is much faster), which we measured by FCS. This is, however, just a scaling factor that defines the absolute value of the binding free energies (the relative free energies for all interactions are independent of this factor: it is just a constant free energy value added/subtracted to all parameters). We now explain this more clearly with the following sentence:
"where w_0 is a proportionality constant that represents the ratio between the diffusion controlled rate constant for association (k_{on}^{0}; in $M^{-1}s^{-1}$) and the dissociation rate constant in the absence of interactions between EngHD and DNA (k_{off}^{0}; in s^{-1}). …..Here we set w_0 to $5{\cdot}10^{-4}$ M^{-1}, consistently with EngHD's diffusion coefficient (determined by FCS as ~122 $\mu m^2 s^{-1}$, see mat. sup.). However, its exact value is of no practical consequence since it is just a scaling factor for $\Delta G_x^{Binding}$."

2) The model (Eqn 8 in supplement) is based on knowledge of the 3D structure of the protein bound to its consensus site, and assumes, by and large, that the sequence-dependent binding energies are dictated by the (position-specific) nucleotides seen in this consensus site. This key assumption has not been justified. For instance, the motifs for Engrailed at FlyFactorSurvey, based on Bacterial 1-hybrid technology, suggest that the last two positions allow 'G', but 'G' at these positions may not be weak interactions.

The original statistical mechanical model was strictly based on the 3D structure of the complex as reviewer 3 correctly indicates. The assumption behind this model is the canonical one in structure-based modeling, which has such a long history of successes in molecular biophysics and biophysical chemistry that we found the need to justify it superfluous. The main idea behind the approach is that the structural contacts between bases and aminoacids of EngHD that are present in the complex (whether hydrogen bonds or hydrophobic interactions between the bases and aliphatic side chains) reflect the formation of favorable interactions that stabilize the complex. When a base is substituted by another one in a given site, the possibility of forming the same interaction is removed given the different chemistry and geometry of the 4 bases. This idea

of rationalizing the energetics of any given biomolecular process (binding, folding, assembly, catalysis) from the interactions observed in the relevant 3D structures is as old as structural biology (starting with Max Perutz and his rationalization of the T and R states of hemoglobin from the salt bridge network formed/broken in the X-ray structures of deoxygenated and oxygenated hemoglobin).

As for the comment regarding the possibility of finding a G in fifth and sixth positions, we agree with reviewer 3 that the observation of a contact (or occurrence) in a given molecule or complex is not per se an indication of the presence of a strong interaction. However, if there is a contact in the structure, there is an interaction. What is not clear from the structure is whether the interaction is strong, weak or even unfavorable. This is why we used the structure to define the most relevant interactions, but we then parameterized their values using targeted experiments. However, in response to reviewers 2 and 3, we have, in the revised manuscript, included two different statistical mechanical models for EngHD binding to DNA, one in which the energetics are based on the 3D structure of the complex (although simplified from the original one, see below), and one based on the PWM. Both models work well, although the structure-based model works much better (objectively and also according to the Fisher test, which accounts for the difference in statistical complexity: i.e. number of fitting parameters). The comparison between the two models given in the revised manuscript should satisfy these questions.

3) No discussion of parameter estimation of the statistical mechanical model of Eqn 8 is provided. How many sequences are fit to? How reliable are these parameter estimates (reported in Figure 3B)? How good is the fit?

Our original modeling approach was to define the possible interactions based on the 3D structure of the complex, use specific experiments designed to independently set the values of each individual parameter, and then test the predictive power of the model with all other experiments. This approach tests the significance of model from its performance in predicting experimental results that it had not seen before and were completely independent from the training set (in our case using DNAs with variations on the target site only to predict the binding behavior of molecules with increasing segments of biologically relevant flanking regions). We are strong believers on the strengths of this approach which we have successfully used before in the context of protein structure prediction and protein folding and assembly.

However, the downside is that such approach makes it difficult to rigorously evaluate the statistical significance of the parameters and their values. Thus, in response to the comments of reviewers 2 and 3, we decided to use a more standard statistical approach of fitting the model parameters to all the experimental data globally, and estimate the statistical significance of the parameters using covariance analysis (from the Jacobian matrix of the best fit). The statistical significance for each parameter emerging from the global data analysis is now given in the revised table of figure 3. We also provide a plot of the experimental versus calculated affinities for all the DNAs used in this study as new figure s1. New figure s1 includes calculations with the reduced version of the structure-based model as well as with a new model based on the PWM for Engrailed, which we implemented per suggestion of reviewer 3 (see below).

As explained above, we have extensively worked in finding out the simplest possible model (in terms of types of interactions and also number of fitting parameters) that could still reproduce our data. Particularly, we performed principal component analysis to find an optimally reduced parameter set for the statistical mechanical model**, which led to a structure-based model with only 4 parameters** and still good performance (i.e. judged as the sum of least squares for all of the data). The four reduced interactions are still based on the 3D structure and consist of: 1) for each consensus base in the core tetrad; 2) for A or T in positions 1 and 6 and non-consensus A or T in the core tetrad; 3) a cooperative term for the full consensus sequence (or half its free energy when the consensus site lacks the first or last base); 4) electrostatic interaction term.

We also tried another model implemented with base-specific interactions derived from the available 6x4 position weight matrix (PWM) for Engrailed together with the non-specific electrostatic term that we have used for all of the structure-based models (this model has 6x4 fixed parameters from the PWM plus the electrostatic term as only fitting parameter). We found that the reduced structure-based model with 4 parameters performs much better (about 50-fold better sum of least squares) than the PWM-based one (see new figure s1). To determine the statistical significance of both models we performed the Fisher test, which indicates that the probability that the performance of the statistically simpler model (PWM with just 1 free parameter) is comparable to that of the complex one (structure-based with 4 parameters) is lower than 10^{-9}. Accordingly, in the revised manuscript we have finally opted for the reduced structure based model (rather than the original or the PWM-Electrostatic models), which we now describe in depth in the main text rather than in supplementary materials. Nevertheless, anticipating interest from a section of the potential readers of this work, we describe the PWM-based model as well, and compare it with the structure based model (both in terms of performance and statistical significance).

4) How is the partition function calculated for longer sequences that may harbor overlapping binding sites? How is the exponential time complexity of the calculation addressed?

The partition function grows as 2xDNA length (see equation 1). Therefore, the increase in computing time required to calculate the partition function and binding profile increases linearly with the DNA length. This means that it is relatively straightforward to calculate the partition function for DNAs as long as 1Mb. However, when the DNA is very long, the calculation of an overall affinity becomes somewhat non-sensical because it is not of much use to know the total occupancy when it is distributed over such very long distances. This is why for the binding profiles of full gene sequences (from 5 to 50 kbp), we calculate the partition function and binding free energy landscape for the whole sequence (the patterns in teal color in figures 6 and 7) whereas the K_D values are shown as effective K_D for fragments of 75 bp (i.e. each K_D value is calculated as the overall K_D for all the individual sites present in each 75 bp segment of the DNA). We use 75 bp to reduce the site-to site binding fluctuations (i.e. to make the binding patterns over longer sequences easier to detect) while using a DNA binding unit that is still sufficiently short as to make the overall K_D values meaningful from the viewpoint of specific location. This definition is, however, used as an analytical tool rather than being a technical limitation.

5) Is it true, as Equation 6 seems to suggest, that only microstates with at most one site bound to the DNA molecule are considered? Previous models (e.g., PMID 19956545) allow for microstates with multiple bound sites. Does it make a difference, especially since the focus here is on DNA sequences with clusters of weak sites?

Binding of a TF bearing a single DNA binding domain (e.g. a homeodomain) to any site in a DNA molecule occurs, for the most part, independently of binding to any other site (binding events are essentially independent, and energetically decoupled). This means that the probability of two TF molecules simultaneously binding to two distant sites can be obtained simply as the product of the probabilities for binding to each site calculated with the partition function from equation 1. The exception to this rule is for the six binding sites that partially overlap with a given (central) binding site, in which case simultaneous binding is sterically forbidden. However, when the concentration of TF is much smaller than the number of available binding sites, the local overlap factor can be ignored because the probability that two TF molecules will try to access overlapping binding sites is negligible. In our experiments, and also in typical *in vivo* conditions, the concentration of TFs is ~1nM and thus the number of TF molecules is miniscule compared to the number of available binding sites (determined by [DNA]x2x(L_{DNA}-5)); accordingly, we utilize the fully independent model represented by the partition function given in equation 1. The only conditions in which this simplification makes a significant error is when the TF concentration reaches near saturation of all possible binding sites (for example in an experiment with 10 nM of a 75 bp DNA, the TF would need to be at concentrations >1.4 μM, or in a mammalian cell with 2% of its DNA being transcriptionally active (~50 Mb) the overall TF concentration would have to be > 5 μM), in which case the probability for occupying a central site is essentially 1/6 of the probability determined with equation 1 (or somewhere in between these extremes for conditions near but below saturation).

"EngHD engages in three different types of DNA binding, the classical specific (consensus) and non-specific binding modes, plus a new mode consisting of promiscuous specific binding to degenerate versions of the consensus sequence." The B1H motif for EN (Fly Factor Survey) also suggests that the TF can bind promiscuously to mismatched versions of the consensus sequence. Please clarify what is the new finding using the FSC data and stat mech model.

The B1H motif for Engrailed does show indeed certain heterogeneity for binding that is suggestive of promiscuous binding. However, the **PWM from these experiments greatly underestimates the actual ladder of binding affinities**. For example, in the core tetrad, the consensus base is found >90% (95% for the two central bases), and all other base types are only found with 2-3% frequencies. For the two most extreme bases (positions 1 and 6), the PWM still gives 75% probability for the consensus, and then almost all that is left (23%) is for just one other base: G (for 1) or C (for 6). This is why the PWM predicts huge drops in affinity with small deviations from the consensus sequence (see new figure 2c), grossly underestimating the binding resilience that we observe in quantitative biophysical binding assays using a broad dynamic range technique such as FCS. This is not much of a surprise because high throughput binding assays are specifically designed to detect only very high affinity binding, which is a requirement to minimize the false positives that are always the main culprit of high throughput experiments. In fact, many researchers in the field would consider frequencies of 1 or 2% in the PWM to be false positives.

By performing a detailed biophysical characterization of the binding properties of EngHD to DNAs that include a natural flanking sequence (of varying length and sequence) combined with detailed statistical mechanical modeling, we could determine the full ladder of binding affinities, going from purely non-specific DNA binding (driven by electrostatics) to full consensus, as well as all of the promiscuous binding affinities lying in between. The surprise was that specific (or quasi-specific) binding is much more degenerate than anticipated from PWM data, to the extent that promiscuous binding to degenerate consensus repeats dominates the occupancy of the TF to the DNA regulatory regions that contain target sites. **This result was completely unexpected because the focus for many decades has been on either specific binding to the target site (with little variation around the consensus motif as in the binding logos) or completely non-specific (sequence independent)**. What we find is that most of the actual binding events in which a eukaryotic TF is engaged are neither, but are instead of this new class of binding to degenerate consensus repeats, which dominate due to their mid-affinity and sheer numbers. We could not have obtained such conclusive results without the combination of quantitative biophysical binding experiments and statistical mechanical modeling.

Moreover, when we used the statistical mechanical model to analyze bioinformatically the sequences of genes controlled by Engrailed, we could then unambiguously characterize the patterns of long tracts of degenerate consensus repeats in the regulatory regions of relevant genes, and quantitatively determine that most of the actual binding of Engrailed will be concentrated in these degenerate consensus tracts rather than in the few specific target sites. This is an idea that has been going around for some time (as several reviewers including 3 point out; and as we now discuss in more depth in the revised manuscript's introduction). But, it is not possible to assess its true significance as a functional mechanism unless a complete quantitative description of the promiscuous binding of the TF to the relevant regulatory gene regions is obtained. That is mostly what we have done for Engrailed by trailblazing in between the parallel views obtained from the vast amount of (highly filtered for highest affinity) binding data provided by high throughput and functional genomics approaches for specific binding, and from advanced biophysical experiments, simulations and theory for non-specific (without base recognition) binding as the foundation for facilitated 1D diffusion.

"Contributions from flanking DCB could in fact explain why deviations from the consensus sequence on the SB site produce only minor affinity decreases. For example, replacement of A6 by T involves the loss of 4.8 kJ/mol in binding free energy, equivalent to a seven-fold drop in affinity, but the KD increases by only 4-fold (m2 vs. m1 in fig. 4b)." Yes, but this specific example could be more simply explained by the trained model being inaccurate. I was under the impression that the model was trained on all available affinities (not just for the consensus sequence). Is this not correct?

As we explained above, the original model was parameterized using specific experiments for each parameter (data in former figs. 2a and 2b and DNA molecules m4 and m5 from fig. 4). We now present an optimally reduced statistical mechanical model (with only 4 parameters) that has been parameterized using all the data globally (see above). However, the point that we were making in that excerpted paragraph does not depend on the model, but on the binding resilience that we experimentally observe in the DNA variants with changes in the target site (figure 2). We

were just using the parameters calculated from the model to illustrate the point quantitatively. However, in the revised manuscript we now introduce a calculation of the drop in binding affinity upon mutation as determined from the frequencies of the PWM that illustrates this point more clearly and also in a more powerful way.

The revised text now reads: *"These experiments reveal evident changes in affinity. However, the decreases in affinity are seemingly small relative to how much the binding site diverges from the consensus, most notably for CGTGTT in which only one consensus base is retained. To further investigate this issue, we compared our affinity results with the position weight matrix (PWM) for Engrailed obtained from bacterial one-hybrid assays[40]. The PWM recapitulates the consensus binding site (fig. 2b), and predicts a decreasing binding probability as the target site sequence diverges. However, the PWM predicts changes in binding probability that are much sharper (many orders of magnitude) than what we find in our experiments (fig. 2c). The PWM also predicts huge differences in binding (several orders of magnitude) for target sites that exhibit very similar affinity in the context of the 75 bp β3-tubulin intron DNA fragment and our experiments. The implication is that EngHD DNA binding is not just dictated by specific interactions with the consensus motif but is more complex. The most likely explanation is that EngHD binds promiscuously to the flanking DNA sequence to an extent that partially compensates the degradation and even elimination of the consensus site."*

"The statistical mechanical model does indeed explain these apparent discrepancies as arising from rugged binding free energy landscapes with many local minima corresponding to DCB sites (fig. 4c)." How? I don't see how Figure 4C explains the apparent discrepancies.

This point has to do with the fact that the local binding minima observed in the binding free energy landscapes (arising from the many degenerate consensus binding sites) provide binding resilience to mutations by becoming partially occupied when there is not a high affinity specific site dominating the occupancy. It is easier to understand this point by looking at the occupancy profiles rather than the free energy landscapes. Therefore, we have modified figure 4 to include the occupancy profiles together with the binding free energy landscapes so that this point is more apparent.

We have also modified the text, which now reads: *"Calculation of the binding profile for this DNA region with our model shows a rugged binding free energy landscape with many minima. However, the local minima concentrate around the target site, producing an overall funnel for EngHD binding (m1 in fig. 4c)……………………..A rugged funneled binding landscape also introduces resilience to mutations on the specific site, which is exactly what we observe in the experiments of fig. 2a. The mechanism behind binding resilience becomes apparent by comparing the binding occupancy of the natural sequence (with TAATTG in the target site) and the sequence with a consensus, or SB, site. The latter produces a landscape with a global minimum that concentrates most of the occupancy (m1 in fig. 4c), whereas the natural sequence lacks the sharp free energy minimum but maintains all flanking DCB sites (m2 in fig.4c), which in absence of the competing SB site see their occupancy raised, thereby buffering the overall drop in affinity."*

"Experiments with m3 showed a marked decrease in binding affinity with KD of ~0.7·10-6 M, or

a 21-fold decrease relative to m2 (fig. 4b)." This is interesting to see, from experimental results. It would be nice to know how the computational estimates of binding strength would have compared (between m2 and m3) if the estimates were made by scoring for presence of known PWM of EN (From Fly Factor Survey), using sum (exp (LLR(site)) over the entire sequence.

We have taken this suggestion to heart (also realizing how much high throughput binding data shapes the view that researchers in the field have of how TFs interact with DNA). Accordingly, we have built an alternative statistical mechanical model in which the specific interactions are directly derived from the frequencies for finding each base on any of the six positions of the target site found in the available PWM (simply as $E_{pos,base}=-RT\ ln(f_{PWM}/0.25)$ in which a random distribution of bases is used as reference). In addition to this 6x4 parameter set for consensus interactions, the model included the electrostatic interaction to account for non-specific (sequence independent) binding and the effects on binding affinity of the ionic strength (former fig. 2b, new fig. 2d). Once we fitted the electrostatic term to all the data at various ionic strengths (this term also scales the free energies), this version of the model reproduces the overall trends in the data, but its performance is about 50 times worse (in terms of global sum of least squares) than our reduced, 4-parameter structure-based model. A Fisher test gives a probability lower than 10^{-9} that the PWM-based model is comparable to the structure-based one once their different statistical complexity is taken into account. We are, however, describing both models in the section on statistical mechanical modeling (which is now in the main manuscript), and also show a comparison of their performance in the new figure s1.

An important claim made by the authors is "the effects from the flanking sequence arise from DCB instead of from sequence-independent base clustering". I agree with this, in light of the data and the model. But it seems to me that this is confirming what is generally believed to be the case. When prior literature talks about facilitated diffusion, doesn't it assume that the facilitating sites invoke sequence-dependent binding? I may be mistaken about this, but that was my understanding.

When researchers in the field talk about facilitated diffusion they define it as a mechanism resulting from the ability of TFs to bind to DNA in a non-specific way, meaning that the TF binds with low affinity to any DNA sequence without engaging in specific interactions with the bases and mostly driven by electrostatic interactions between the positive charge of the TF and the phosphate backbone of DNA. This description of non-specific binding and facilitated diffusion are the original ones given by von Hippel in his seminal works on facilitated diffusion (see for example refs. 3 and 4), and what causes 1D sliding over DNA as mechanism to accelerate the search. Most of the biophysical work on DNA binding and diffusion refers to this type of binding that is devoid of base recognition.

The most significant idea/claim presented by the authors is "DCB transforms the β3-tubulin RR onto a potent attractor for EngHD molecules, which could accordingly operate as a transcription antenna to ensure local availability of the transcription factor as well as low occupancy of the specific site." This is based on "a 500-fold affinity increase for the entire β3-tubulin gene, relative to its high affinity SB site alone (fig. 5b)", as predicted by their model. However, this extrapolation is not justified. It is entirely possible for a model to operate up to a

scale of 600 bp but not extend to a scale of 10000 bp. For instance, models of enhancer function that work on ~1 Kbp scale do not work at the ~10 Kbp scale.

Although we cannot think of any obvious physical factors that could change the mechanism of binding of a TF to DNAs when they go from 600 bp to a few thousand kbp (TF binding is very local provided that the DNA is naked and in B-from), we do see that the point raised by reviewers 2 and 3 could resonate with part of the potential readership of this work and wanted to address it. Here we were pressed between a rock and a hard place because testing longer DNAs really push the limits of sensitivity of our binding assays (which are still superior for testing this question than other available alternatives). However, we did synthesize the a DNA of 7,219 bp that contains the entire β3-tubulin gene sequence without the 5' and 3' UTRs. This sequence contains the very long intron 1 where our target site is, and thus contain a significant fraction of the gene's regulatory region, but by dropping the UTRs we could decrease the predicted overall affinity somewhat, and thus investigate its binding using FCS. The FCS experiments do not allow to determine a K_D for this DNA with the same level of accuracy, but they do show very clear binding (diffusion retardation) of EngHD at very low concentrations of even 100 pM). These experiments indicate that the overall K_D of EngHD to the 7,219 bp β3-tubulin gene DNA is sub nM (our upper estimate for the K_D is for it to be ~700 pM). We now show the FCS experimental data for this very long DNA in the revised manuscript as figure 5c. We also plot the value of affinity determined experimentally in the revised figure 5b, together with the affinity predicted by the reduced 4-parameter structure-based model.

The 'gcPBM' technology provides binding intensity measurements of hundreds of thousands of binding sites in their genomic context. Such data are available for several TFs. Could similar results have been obtained (as those presented here) by analyzing the gcPBM data using PWM motifs? Such an approach would not require the hand-crafting of the stat mech model presented here.

As we said above, we now include an alternative model to our structure-based one that uses PWM data for Engrailed as the basis for calculating pairwise specific interactions. The two models are described in the section on statistical mechanical model (now part of the main text of the revised manuscript). The performance of both models is also discussed in the manuscript, and their comparison relative to the experimental data is given in the new figure s1.

Reviewer 4

This is a paper that combines biophysical experiments and theory to analyze the mechanisms of transcription factors recognizing their specific binding sites in eukaryotic cells. The subject is very important, and authors made a lot of work to clarify the very complex nature of these phenomena. There are several interesting ideas and results, and I like this work. I think the paper is potentially publishable but authors have to improve in many cases and many questions must be answered.

We appreciate reviewer's 4 general comments and appreciation for the importance of our work. We also thank this reviewer for her/his suggestions to improve our work, which we have carefully followed. Our response to each individual comment are given below.

My specific comments are:
1) Generally, it is implicitly assumed that the transcription factors binding to their targets in cell is an equilibrium process. This is clearly not correct. So the consideration of affinities is important, but it is not enough to explain the dynamics of transcription factors binding. Authors should emphasize this.

We fully agree with reviewer 4 that the process of transcription factor binding is a combination of two important aspects, the binding affinity and the search kinetics. We also agree with this reviewer that often times the kinetic aspects of the problem are overlooked. Our work is based on measuring binding affinity, and thus, the equilibrium aspects of the problem are centerstage. However, our measurements of affinity are of cumulative occupancy rather than of canonical single-site binding. The consequence is that, in our measurements, the kinetic aspects of the process also play a key role by determining the overall affinity as a dynamic exchange between a very large number of local mid-affinity binding sites. The conclusions of our work are that it is the interplay between myriads of binding events of moderate affinity what allows spatial co-localization without kinetic trapping. The key here is that the mid-affinity of the degenerate consensus binding sites makes it for relatively fast dissociation rate constants, and extremely fast rebinding (due to the high local concentration of potential binding sites). This key kinetic aspect originates from our binding measurements (from the K_D values one can roughly estimate dissociation rate constants), but it is also what percolates from the simple kinetic model of the transcription antenna mechanism that we originally presented in the supplementary materials. In response to reviewer 4, we have now included the description of the transcription antenna kinetic model in the main text of the revised manuscript (a new section), and we also further emphasize the kinetic underpinnings of the transcription antenna mechanism in this section. We also have included several references to prior theoretical work that had considered similar binding scenarios (see response below).

2) There is a statement in the Introduction that for prokaryotic TF the targets are found by 1D sliding. This is clearly not correct statement. It is combination of sliding, binding from the bulk solution and intersegment transfer, and maybe other processes. See, e g., experimental and theoretical work in Nucl. Acid Res. 42, 7039 (2014), J.Phys. Chem. B 117, 12695 (2013).

We thank reviewer 4 for pointing this out. It was not our intention to simplify the process to just 3D and 1D sliding, what we were trying to say is that the combination of specific and non-specific binding suffices to explain transcription in prokaryotes. Upon re-reading the introduction, we realized it was not sufficiently clear. We have now rewritten this section of the introduction to also discuss other mechanisms of facilitated diffusion by non-specific binding, and specific that it is the combination of all those that are important in prokaryotes. The revised section now reads: *"Non-specific binding facilitates the search for the target site by allowing the TF to slide along DNA via a relatively slow, but efficient, one dimensional diffusive motion*

($D<10^{-8}$ cm^2s^{-1}) [4] *that involves rotation about the DNA axis*[5] *and covers distances between 300 and 10,000 bp* [6]*. Another mechanism of facilitated diffusion occurs when the TF is transferred between DNA regions that are transiently in spatial proximity*[7]*. These various non-specific binding modes act jointly to increase the kinetics of binding of the TF to its target site*[8]*. For instance, in vivo imaging experiments in bacteria indicate that the combination of these molecular elements are sufficient to explain the homing, selectivity and occupancy of prokaryotic TFs*[9]*. "*

3) The statistical mechanical model (as well as the kinetic model) must be explained better. It is a very phenomenological at this point. It has 8 parameters, as far as I can understand. But how many experimental data have been used to fit the data? 3 points in Fig. 2b is not enough. I think it is an overstatement (page 3) that the model captures the fundamental properties in the system. What things calculates the kinetic model?

Following comments by all reviewers, who consistently requested a better explanation of the statistical mechanical model, the fitting process and also to provide quantitative analysis of the significance of the parameters, we have very thoroughly revised the model and its parameters. First, we do now describe the modeling in the main text of the paper rather than in supplementary materials. We have also done a very extensive comparative analysis of multiple models, including models that use the position weight matrix (PWM) data from high throughput binding experiments on Engrailed, and structure-based models of increasing simplicity (parameter reduction). We have also changed the fitting approach to determine the statistical significance of the parameters and thoroughly compare the performance of different models.

In our original modeling exercise we used a structure-based approach very similar in spirit to structure-based models used in other areas of molecular biophysics (such as protein folding). Accordingly, we defined the possible interactions between Engrailed and DNA based on the 3D structure of the complex, and then used specific experiments designed to independently set the values of each individual parameter. Finally, we tested the predictive power of the model with all the other experiments that had not been used before to parameterize the model. In this approach, the significance of model comes from its performance in predicting experimental results that it had not seen before and were completely independent from the training set (in our case we used the DNAs with variations on the target site and the ionic strength dependence data of figure 2, plus DNAS m4 and m5 of figure 4 to determine the 8 parameters, and tested the model by predicting the binding behavior of molecules with increasing biologically relevant flanking regions, figures 4 and 5). We are strong believers on the strengths of this approach which we have successfully used before in the context of protein structure prediction and protein folding and assembly.

However, the downside of such approach is that it makes it difficult to rigorously evaluate the statistical significance of the parameter values. Thus, in response to the comments of reviewers 2, 3, and 4, we decided to use a more conventional statistical approach of fitting the model parameters to all the experimental data globally, and estimate the statistical significance of the parameters using covariance analysis (from the Jacobian matrix of the best fit). The statistical significance for each parameter emerging from the global data analysis is now given in the revised table of figure 3. We also provide a plot of the experimental versus calculated affinities

for all the DNAs used in this study as new figure s1. This figure includes calculations with the simplified version of the structure-based model that we have produced as well as with a new model based on the PWM for Engrailed, which we implemented per suggestion of reviewers 2 and 3.

As mentioned above, we have extensively worked in finding out the simplest possible model (in terms of types of interactions and also number of fitting parameters) that could still reproduce our data. Particularly, we performed principal component analysis to find an optimally reduced parameter set for the statistical mechanical model**, which led to a structure-based model with only 4 parameters** and still good performance (i.e. judged as the sum of least squares for all of the data). The four reduced interactions are still based on the 3D structure and consist of: 1) for each consensus base in the core tetrad; 2) for A or T in positions 1 and 6 and non-consensus A or T in the core tetrad; 3) a cooperative term for the full consensus sequence (or half its free energy when the consensus site lacks the first or last base); 4) electrostatic interaction term.

We also tried another model implemented with base-specific interactions derived from the available 6x4 position weight matrix (PWM) for Engrailed together with the non-specific electrostatic term that we have used for all of the structure-based models (this model has 6x4 fixed parameters from the PWM plus the electrostatic term as only fitting parameter). We found that the reduced structure-based model with 4 parameters performs much better (about 50-fold lower sum of least squares) than the PWM-based one (see new figure s1). To determine the statistical significance of both models we performed the Fisher test, which indicates that the probability that the performance of the statistically simpler model (PWM with just 1 free parameter) is comparable to that of the complex one (structure-based with 4) is less than 10^{-9}. Accordingly, in the revised manuscript we have finally opted for the reduced structure based model (rather than the original one), which we now describe in depth in the main text rather than in supplementary materials. We also describe the PWM-based model and compare it with the structure based model (both in terms of performance and statistical significance).

4) page 4 - what is the "local base clusters"? It is not clear to me. Actually, authors criticize the theory due to Lukatsky and coworkers, but if you look at other papers of Lukatsky (not referred here) then the findings of this work actually agree. Lukatsky argued that the increase effective affinity to the specific sites is due to effective interactions from the flanking regions - exactly what is found in this work. I recommend to modify the discussion of these issues.

By local base clusters we referred to tracts of repeats of the same base (e.g. repeated patterns such as AAA or TTT) that lead to certain local sequence symmetries. This is the type of sequence pattern in regions flanking target genes that Lukatsky and coworkers have worked on for several years. Reviewer 4 is correct that Lukatsky and coworkers have explored very actively the effects of the flanking sequence around transcription factor target sites. From that viewpoint, there are clear similarities between their work and what we do find: effects of the flanking sequence on binding affinity, and overall occupancy. We do agree with reviewer 4 that the mechanism proposed by Lukastky and coworkers is conceptually the closest one available to our findings, and this is precisely why we specifically investigated the contribution of their mechanism for enhanced binding by the flanking region to our data on EngHD (including DNA

molecules we purposely designed to distinguish between their mechanism and the promiscuous binding to degenerate consensus repeats that we propose).

In this regard, we should also say that there is a very clear difference between the two mechanisms. In their published papers (we now cite three of them: refs. 27, 28 and 29 of the revised manuscript), Lukatsky and coworkers are very specific about the effect they are proposing, which they categorize as non-specific binding, that is, binding that does not depend on base recognition. This point of binding that does not involve base recognition (or is non-consensus) is central to their mechanism and very clearly stated in all their publications, as can be easily assessed from just their titles. For example, their PNAS 2014 is entitled: "*Protein-DNA binding in the absence of specific base-pair recognition*" (new ref. 28), or their PLoS One in 2015 entitled: "*Nonconsensus protein binding to repetitive DNA sequence elements significantly affects eukaryotic genomes*" (new ref. 29).

Therefore, the mechanism of transcription antennas in EngHD that we propose and characterize experimentally, theoretically and bioinformatically is completely different from Lukatsky's in that it involves promiscuous binding to tracts of degenerate versions of the consensus sequence, and thus is quasi- or semi-specific, and is also mid-affinity (tighter than non-specific and softer than specific). In any event, we have now changed the manuscript to reference the work of Lukatsky and coworkers more extensively (three of their articles are referenced, including their arguably most influential 2014 *PNAS*), provide a distinct statement in the introduction of what their mechanism is: "*The potential roles that repetitive sequence patterns flanking the cognate site may play on how eukaryotic TFs find their target have been recently subject to intense scrutiny. For instance, when surrounding the target site, certain symmetric sequence repeats that do not involve specific-base recognition can affect the TF binding preference by statistical (or entropic) factors*[27, 28, 29]*.*"
We also compare the two mechanisms (Lukatsky's symmetry-driven enhanced non-specific binding vs. promiscuous binding to degenerate consensus repeats) and how each contributes (or not) to our binding data in the **The binding free energy landscape of the DNA region flanking the target site** section of the revised manuscript: *"An alternative explanation is that binding enhancement is caused by the accumulation of symmetric base repeats in the flanking region, a factor that has been proposed*[27]*, and found in the TF Max*[28]*, to entropically stimulate binding in the absence of base-specific interactions. The β3-tubulin intron fragment does contain many base repeats (m2 in fig. 4a). We thus tested this possibility experimentally using a DNA that maintains the β3-tubulin original base composition, but eliminates base repeats (m5 in fig. 4a). We used the low affinity CGTGTT as target site, aiming to minimize SB contributions and thus increase the sensitivity of our experiments to differences between DCB and NSB. The statistical mechanical model does not include non-specific effects from symmetric base repeats, and, accordingly, it calculates minimal binding differences between m5 and the β3-tubulin sequence (m4). FCS experiments also show minimal differences (fig. 4b), confirming that the flanking DNA effects we see in EngHD arise from promiscuous DCB instead of from non-specific base repeats."*

5) The effect of semi-specific sites and the role of the flanking regions on dynamics of TF have been explored theoretically, and authors seems unaware of this. Look at J. Chem. Phys. 143,

245101 (2015), where the dynamics of finding the target was analyzed using the analytically solvable model. Also see J. Phys. Chem. B 119, , 12410 (2015). Many similar results are found in these papers. Some discussion of these connections might be useful.

The articles mentioned by Reviewer 4 do indeed investigate the role of semi-specific binding sites located in DNA regions flanking the target sites of TFs using theory via an analytically solvable kinetic model. In these works the authors mention semi-specific sites that they characterize as kinetic traps (and thus have stronger binding than non-specific sites), and which are found to be able to accelerate the search for the target site when they are located in its vicinity. This result is very interesting because it is in principle counterintuitive (kinetic traps are normally associated with a slowdown of the overall kinetics), unless there is an optimized balance between the rate of dissociation, the diffusion coefficient and processivity of the sliding mode, and the distance between traps and target site. These works are indeed very relevant to what we find experimentally and propose theoretically. In the revised manuscript we have added these references and discuss at length the connections between our results and transcription antenna mechanism and the sophisticated kinetic effects investigated in these theoretical papers. We thank reviewer 4 for pointing them out to us.

For instance, the new **introduction** includes the sentence: *"Moreover, theoretical modeling indicates that a flanking DNA sequence that is heterogeneous[32], or contains weakly competing binding sites[33], could accelerate the TF search for its target site."*

The section **A simple statistical mechanical model of EngHD-DNA binding energetics** includes the following paragraph: *"Semi-specific binding of eukaryotic TFs to clusters of degenerate consensus repeats around a cognate site has been proposed to increase the site's occupancy in vivo[30, 31] and to accelerate the search for the target site[33]. Here we determine its actual contribution to overall binding and dissect its molecular mechanism. The key questions that emerge are: how does the interplay of these three binding modes define the overall binding behavior of EngHD? and what are its functional implications?"*

The section **The binding free energy landscape of the DNA region flanking the target site** includes the following sentence: *"An important implication is that EngHD can occupy multiple mid-affinity binding sites surrounding the target site in a strategy that enhances the overall affinity without involving very strong interactions, that is, while maintaining relatively fast dissociation rates. This behavior is consistent with predictions from theoretical modeling[33]."*

The section **The binding free energy landscape of the DNA region flanking the target site** includes paragraph: *"An important implication is that EngHD can occupy multiple mid-affinity binding sites surrounding the target site in a strategy that enhances the overall affinity without involving very strong interactions, that is, while maintaining relatively fast dissociation rates. This behavior is consistent with predictions from theoretical modeling[33]."*

6) The picture proposed by the authors (DNA antennas) can work if the interactions in the flanking regions is not too strong and not too weak. So there is some optimality in the number

and the strength of the degenerate sites. These degenerate sites must be similar but not too similar or not too dissimilar. It should be emphasized.

Reviewer 4 is absolutely correct in that what makes the antenna work is an optimal balance between the strength of each binding event and the number of them and their location. Larger numbers of mid affinity sites implies a larger overall occupancy around the target site (but also an increasingly long average distance to it and eventually trapping if these interactions are too strong). We think that the revised version of the manuscript, which now includes the whole description of the transcription antenna kinetic model as new section **A simple kinetic model for the operation of a transcription antenna**, makes this emphasis as requested by reviewer 4. This issue is described in several sections of the manuscript, including this paragraph: *"Therefore, the antenna mechanism contains two complementary elements. The first element is to act as a trap that retains EngHD molecules in myriads of stochastic binding events to DCB sites. Trapping occurs because the exchange between the antenna and the cellular milieu (EngHD molecules either free or bound somewhere else in DNA) depends on the infinitesimally small probability of finding an unbound EngHD molecule within the antenna (square bracketed species in fig. 6c), and thus it is very slow. A slow diffusive exchange with the outside creates a dynamic microenvironment wherein the pool of EngHD molecules is still able to redistribute in response to chromatin dynamics[52, 53], or even larger scale cellular changes. The second key element is the fast exchange of EngHD molecules that takes place among antenna sites (including the higher affinity target site) either by dissociation —facilitated by the relatively high dissociation rates of the mid-affinity degenerate consensus sites within the antenna— and quick rebinding (1 in fig. 6c), or sliding (2 in fig. 6c), and which is consistent with recent theoretical work[33]. It is therefore apparent that such antenna guarantees a local supply of EngHD molecules, a quick turnover over the target SB site, and fine control of SB site occupancy via binding competition with the antenna's DCB sites."*

Reviewers' comments:

Reviewer #2 (Remarks to the Author):

This is a resubmission of the manuscript by Castellanos et al, where they combine high-quality biophysical measurements of TF binding to diverse DNA contexts and statistical analysis to propose that specific TF binding sites are surrounded by antennae regions that attract TFs.

In this revised manuscript the authors have addressed several questions and comments that have been raised. Most importantly, they have revised their model, to only include 4 parameters, which are now associated with statistical errors and have been tested for importance. They have further included a more complete discussion of model design in the main text of the paper. Moreover, they have compared their model to ChIP-derived data, as a further control. Finally, they have reworded several sections to make the paper more understandable. With these changes the paper has clearly improved. I have still some questions however:

1. In my earlier comments, I wondered if the author's model could recapitulate ChIP-seq derived binding profiles. The authors show that DNA segments that bind En are also identify as high-affinity targets by the statistical model. However, the authors do not evaluate other DNA segments which are not supposed to bind En. How does the model perform in RRs that are no target for En?

2. Related to point 1: The profiles in shown in Fig. 6b and 7b show a clear decrease in predicted En binding in coding regions (orange) but there is no "funnel" structure visible around the specific binding sites, but rather the average binding energy profile seems flat. As I understand the antenna concept would predict an increase in DCBs around specific sites. This is however not clear. Thus, it is important to analyze the larger genomic regions, to demonstrate the increase in DCBs at En RRs.

3. The formulation of the model is still not clear to me. It would be helpful to give the complete formula in the SI (how are DG_consensus,core, DG_degenerate,AT etc. converted into DG_k). Also, it would be useful to use the same description/symbols in Fig. 3b and the SI.

minor comments:

- page 5: The reference should be to Fig. 2c.

- page 11: "via myriads of DCB sites localized in the...", I think "myriads" should be changed to a more accurate term.

- The Si contains a lot of text on the model description that is copy-paste from the main text. This should be cleared up.

Reviewer #3 (Remarks to the Author):

The authors have done an outstanding job in this revision, taking all of the previously raised concerns seriously and responding to them with clarifications in text, reorganization of text, and several new analyses and more experimental evidence. I find this revised manuscript to be a solid and convincing piece of work on a very significant topic.

Reviewer #4 (Remarks to the Author):

The paper is significantly modified. Authors fully responded to all my questions. I also that they fully

answered to other reviewers too. I am supporting the publication in the current form.

Point by point response to Reviewer's comments:

Reviewer 2

"This is a resubmission of the manuscript by Castellanos et al, where they combine high-quality biophysical measurements of TF binding to diverse DNA contexts and statistical analysis to propose that specific TF binding sites are surrounded by antennae regions that attract TFs.

In this revised manuscript the authors have addressed several questions and comments that have been raised. Most importantly, they have revised their model, to only include 4 parameters, which are now associated with statistical errors and have been tested for importance. They have further included a more complete discussion of model design in the main text of the paper. Moreover, they have compared their model to ChIP-derived data, as a further control. Finally, they have reworded several sections to make the paper more understandable. With these changes the paper has clearly improved."

We thank reviewer 2 for appreciating our effort to comply with everything that was suggested/requested by the four reviewers of our original submission. The other reviewers are now entirely satisfied with the extensive revision of our manuscript, but reviewer 2 still has a couple of requests that we now address in this second revision.

"1. In my earlier comments, I wondered if the author's model could recapitulate ChIP-seq derived binding profiles. The authors show that DNA segments that bind En are also identify as high-affinity targets by the statistical model. However, the authors do not evaluate other DNA segments which are not supposed to bind En. How does the model perform in RRs that are no target for En?"

This is an interesting suggestion that we ourselves were very curious about. However, although it is quite reasonable in general terms, exploring this question is not straightforward in practical terms. The reason is that while one can readily find information of eukaryotic genes that a given TF controls, the opposite type of relation (genes that are demonstrably not controlled by a given TF) is just not available. And, this is even more so for a TF like engrailed, which controls early stages of development and morphogenesis, and thus controls genes that encoded for proteins of all functionalities, including signal proteins, receptors, protein kinases, protein phosphatases, transcription factors and cell adhesion proteins. This difficulty notwithstanding, we agree with reviewer 2 that it is a very interesting question. So, we decided to look into the Interactive Fly database to pick a relatively good number of genes that, based on their function, were more unlikely to be controlled by engrailed. We identified 24 genes that we are reasonably convinced they are not controlled by engrailed, and found that their EngHD binding profiles do not have the transcription antenna patterns that those under engrailed control have. We have added a full paragraph in the manuscript describing this analysis, and a new supplementary figure (figure s3) with the binding profiles of 6 negative control genes. The new paragraph is as follows: *"As negative control, we looked into the predicted binding patterns for genes not expected to be under Engrailed control. Identifying such genes is not trivial because, as key transcription factor in development, Engrailed controls ubiquitous genes involved in many fundamental morphogenesis, communication and*

signaling processes, including genes that encode for signal proteins, receptors, protein kinases, protein phosphatases, transcription factors and cell adhesion proteins[36]. We thus looked into the Interactive Fly database (https://www.sdbonline.org/sites/fly/ aimain/3a-dtest.htm), which contains a large number of D. melanogaster genes with detailed functional annotations, and eliminated all genes that are involved in any of those processes and/or that are known to be controlled by transcription factors with a similar consensus binding sequence (e.g. most homeoboxes). After applying this filter, we identified a group of 24 genes unlikely to be controlled by Engrailed based on their known function, but still with AT contents not too far from the 57% of the overall Drosophila genome. Even though Engrailed control cannot be entirely ruled out, the binding landscapes for these genes showed significantly lower overall binding affinity for Engrailed than the 39 genes of table s4. In general, these putative negative control genes contained a very small number of high affinity (consensus) sites that were scattered through the sequence, and not surrounded by clusters of degenerate consensus sites. Likewise, no patterns of higher/lower affinity for non-coding versus coding regions are evident in these genes. Figure s3 shows the binding profiles for six of such genes. We thus conclude that whereas genes controlled by Engrailed contain the tracts of DCB around and/or near the SB target site(s) representative of transcription antennas, these patterns are not present in negative control genes. Altogether, our results provide compelling evidence of a systematic usage of transcription antennas by Engrailed."

"2. Related to point 1: The profiles in shown in Fig. 6b and 7b show a clear decrease in predicted En binding in coding regions (orange) but there is no "funnel" structure visible around the specific binding sites, but rather the average binding energy profile seems flat. As I understand the antenna concept would predict an increase in DCBs around specific sites. This is however not clear. Thus, it is important to analyze the larger genomic regions, to demonstrate the increase in DCBs at En RRs."

In this case, Reviewer 2 is getting confused between the structure of the binding landscape of the surroundings of a target specific site, and the global binding profile of a transcription antenna, which extends over many thousands of bps. The rugged funneled structure for binding is present at the local level (in the near vicinity of the target site) and thus is only apparent when binding is shown at single-site resolution. This is exactly the case for the binding profile of the 75 bp DNA sequence that flanks the beta3-tubulin intron 1 target site, which is clearly funneled (please see figure 4c). Figs. 6b and 7b are presented at much lower resolution because they show the binding profile for entire genes and their regulatory regions. In fact, in these plots each datapoint corresponds to the overall binding of a 75 bp segment (that is, the size of the entire binding landscape shown in fig. 4c). In other words, the plots are highly compressed so we can show the entire gene binding profile in a simple plot, and also so that the values of affinity are comparable to the K_D values we obtain from the 75 bp DNA molecules in our experiments of fig. 2. In each of these long gene sequences there are multiple specific binding sites (as is common in eukaryotes), and each of those high affinity specific sites is often surrounded by many DCBs forming a local binding funnel. For example, the high affinity datapoints of the beta3-tubulin gene (i.e. at roughly 4.5, 6.5 and 9 kbp, see fig. 6b) all have a local binding funnel when the free energy landscape is shown at single-site (6 bp) resolution. The high affinity site at 4.5 kpb is in fact the one that we study in depth here, and for which we show the landscape in fig. 4c. Moreover, Reviewer 2 can also note that each of these local funnels is surrounded by a few additional datapoints with relatively high affinity, thereby expanding the binding funnels over several hundreds of bp. In general, the binding funnels are also more apparent in the free energy profile (shown in teal) than in the affinity (or K_D) data (shown in purple).

“3. The formulation of the model is still not clear to me. It would be helpful to give the complete formula in the SI (how are DG_consensus,core, DG_degenerate,AT etc. converted into DG_k). Also, it would be useful to use the same description/symbols in Fig. 3b and the SI.”

Following reviewer 2’s recommendation we have included in the supplementary information exemplary calculations with the structure-based model of the binding free energy for the two binding sites featured in figure 3. We have also added the specific terms for each interaction in the figure 3 legend for easy comparison with the model formulae. The revised figure legend is: *“Black symbols correspond to consensus core tetrad interactions ($\Delta G_{consensus,core}$), gray asterisks correspond to degenerate consensus interactions with A or T ($\Delta G_{degenerate,AT}$). Orange circles correspond to electrostatic (non-specific) interactions, which extend over 8-bp (ΔG_{elec}) and cp is the cooperative interaction that takes place when the site includes the full consensus sequence ($2\Delta G_{cp}$; see model description).”*

And the added text in sup. inf. is: “*As guidance, the two examples of binding sites shown in fig. 3b lead to the following calculations for the binding free energy in the structured-based model:*

$$\Delta G_{TAATTA}^{Binding} = 8\Delta G_{elec} + 4\Delta G_{consensus,core} + 2\Delta G_{degenerate,AT} + 2\Delta G_{cp}$$

$$\Delta G_{TGACAT}^{Binding} = 8\Delta G_{elec} + 1\Delta G_{consensus,core} + 3\Delta G_{degenerate,AT}$$ ”

“minor comments:

- page 5: The reference should be to Fig. 2c.

- page 11: "via myriads of DCB sites localized in the...", I think "myriads" should be changed to a more accurate term.

- The SI contains a lot of text on the model description that is copy-paste from the main text. This should be cleared up.”

We have corrected the typo in page 5, have replaced myriads for hundreds in the two instances where we used this word. As for the repetition of the model description in SI, this was done purposefully. We intend the supplementary information to be a self-sufficient reference for anybody interested in using or reproducing our model. Therefore, we think it is important to repeat the full description of the model in the supplementary information together with the statistical analysis and final parameters.

Reviewer 3

The authors have done an outstanding job in this revision, taking all of the previously raised concerns seriously and responding to them with clarifications in text, reorganization of text, and

several new analyses and more experimental evidence. I find this revised manuscript to be a solid and convincing piece of work on a very significant topic.

We thank reviewer 3 for her/his support to publish our manuscript in Nature Communications.

Reviewer 4

The paper is significantly modified. Authors fully responded to all my questions. I also that they fully answered to other reviewers too. I am supporting the publication in the current form.

We thank reviewer 4 for her/his support to publish our manuscript in Nature Communications.
